# Ecological Risk Assessment of Pharmaceuticals in the Transboundary Vecht River (Germany and The Netherlands)

**DOI:** 10.1002/etc.5062

**Published:** 2021-05-28

**Authors:** Daniel J. Duarte, Gunnar Niebaum, Volker Lämmchen, Eri van Heijnsbergen, Rik Oldenkamp, Lucia Hernández‐Leal, Heike Schmitt, Ad M. J. Ragas, Jörg Klasmeier

**Affiliations:** ^1^ Institute for Water & Wetland Research, Department of Environmental Science Radboud University Nijmegen Nijmegen The Netherlands; ^2^ Institute of Environmental Systems Research Osnabrück University Osnabrück Germany; ^3^ Wetsus, European Centre of Excellence for Sustainable Water Technology Leeuwarden The Netherlands; ^4^ Department of Global Health, Amsterdam Institute for Global Health and Development, Amsterdam UMC University of Amsterdam Amsterdam The Netherlands; ^5^ Department of Infectious Diseases and Immunology Faculty of Veterinary Medicine Utrecht University Utrecht The Netherlands; ^6^ Institute for Risk Assessment Sciences Utrecht University Utrecht The Netherlands; ^7^ Department of Environmental Sciences, Faculty of Science Open University Heerlen The Netherlands

**Keywords:** Pharmaceuticals, Water quality, Ecological risk assessment, Geographic information systems, Environmental modeling, Surface water, Toxic effects, Geo‐referenced modeling

## Abstract

Millions of people rely on active pharmaceutical ingredients (APIs) to prevent and cure a wide variety of illnesses in humans and animals, which has led to a steadily increasing consumption of APIs across the globe and concurrent releases of APIs into the environment. In the environment, APIs can have a detrimental impact on wildlife, particularly aquatic wildlife. Therefore, it is essential to assess their potential adverse effects to aquatic ecosystems. The European Water Framework Directive sets out that risk assessment should be performed at the catchment level, crossing borders where needed. The present study defines ecological risk profiles for surface water concentrations of 8 APIs (carbamazepine, ciprofloxacin, cyclophosphamide, diclofenac, erythromycin, 17α‐ethinylestradiol, metformin, and metoprolol) in the Vecht River, a transboundary river that crosses several German and Dutch regions. Ultimately, 3 main goals were achieved: 1) the geo‐referenced estimation of API concentrations in surface water using the geography‐referenced regional exposure assessment tool for European rivers; 2) the derivation of new predicted‐no‐effect concentrations for 7 of the studied APIs, of which 3 were lower than previously derived values; and 3) the creation of detailed spatially explicit ecological risk profiles of APIs under 2 distinct water flow scenarios. Under average flow conditions, carbamazepine, diclofenac, and 17α‐ethinylestradiol were systematically estimated to surpass safe ecological concentration thresholds in at least 68% of the catchment's water volume. This increases to 98% under dry summer conditions. *Environ Toxicol Chem* 2022;41:648–662. © 2021 The Authors. *Environmental Toxicology and Chemistry* published by Wiley Periodicals LLC on behalf of SETAC

## Graphical abstract

Spatially explicit environmental pharmaceutical risk profiles in the Vecht River basin under 2 climate condition scenarios.

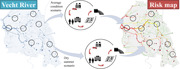

## INTRODUCTION

The discovery and manufacture of active pharmaceutical ingredients (APIs) have prompted human and veterinary medicine to a modern era. Many health care and agriculture food production systems around the globe rely on APIs to prevent and cure a wide variety of illnesses in humans and animals, which has led to a sustained consumption of them (Klein et al. 2018). Next to the benefits of APIs, their widespread use has also led to unintended consequences such as antimicrobial resistance (Young 1993; Hernando‐Amado et al. 2019) and environmental pollution (aus der Beek et al. 2016). The occurrence of APIs in the environment can have detrimental impacts on wildlife (Shultz et al. 2004; Jobling et al. 2006; Saaristo et al. 2018). To guarantee a good surface water quality, it is essential to assess potential adverse effects of APIs to aquatic ecosystems. The corresponding legal framework comprises the European Union's Water Framework Directive (European Commission 2000) and the Priority Substances Directive (European Commission 2008). These directives impose the protection of water resources on European Union member states, for example, by defining environmental quality standards (EQSs) for 45 priority substances. However, none of these substances is an API. Instead, a limited set of APIs is covered in a biennial watch list of water pollutants that should be carefully monitored because of insufficient monitoring data and concerns about their ecological impact. The Water Framework Directive calls for a basin approach, moving away from national risk assessments (Coppens et al. 2015; Vissers et al. 2017) and complementing it with more detailed, in some cases transboundary, catchment‐wide risk assessments. Determination of the chemical status of a surface water within the context of the Water Framework Directive relies on the quantification of risk by integrating exposure and effect assessments.

Exposure assessment can be based on measured environmental concentrations (MECs), predicted environmental concentrations (PECs) using chemical fate models or a combination of both. In the past 30 yr, a variety of models have been developed to derive PECs for chemicals, such as ePiE (Oldenkamp et al. 2018), iStream (Kapo et al. 2016), a contaminant fate model (Grill et al. 2016), PhATE™ (Anderson et al. 2004), STREAM‐EU (Lindim et al. 2016), GLOBAL‐FATE (Font et al. 2019), and the geography‐referenced regional exposure assessment tool for European rivers (GREAT‐ER; Feijtel et al. 1997; Kehrein et al. 2015; Lämmchen et al. 2021), varying in complexity and geographical and temporal resolution. The concentration gradient along a watercourse is highly dependent on local socioeconomic and environmental factors. Therefore, the degree of access to detailed local data (e.g., pharmaceutical consumption patterns) and spatiotemporal information (e.g., seasonal hydrological landscape) is an important driver for the accuracy of exposure models at the catchment level (Tiedeken et al. 2017; Oldenkamp et al. 2018; Font et al. 2019).

A comprehensive effect assessment requires extensive ecotoxicological information to derive safe concentration thresholds for aquatic ecosystems, for example, predicted‐no‐effect concentrations (PNECs) or EQSs. To optimize the accuracy of the assessment, it is common practice to gather all available toxic effect data on a substance and select an extrapolation method that matches the available data. Therefore, the estimation and accuracy of useful PNECs is highly dependent on up‐to‐date ecotoxicological data and requires continuous revision to accommodate new evidence.

Riverine ecological assessments conducted in Europe and elsewhere have recurrently found APIs and other emerging pollutants to pose a potential risk to freshwater biota (Gómez‐Canela et al. 2019). A main obstacle to modeling studies of API residues in transboundary catchments is the restricted access to detailed national and regional API‐specific consumption data (Tiedeken et al. 2017). Additional obstacles include different national and regional water management strategies, diverse wastewater treatment efficiencies, the heterogeneity of the landscape, seasonal variation in environmental conditions, and variable demographics (Popelka and Smith 2020).

The main aim of the present study was to construct ecological risk profiles for surface water concentrations of 8 environmental residues of APIs in the European transboundary Vecht River, a river that crosses several German and Dutch regions. Firstly, an exposure assessment was performed by the applying the geo‐referenced model GREAT‐ER, which has a good track record for predicting pharmaceutical PECs in river catchments (Schowanek and Webb 2002; Capdevielle et al. 2008; Cunningham 2008; Hannah et al. 2009; Alder et al. 2010; Aldekoa et al. 2013; Hanamoto et al. 2013; Zhang et al. 2015; Archundia et al. 2018; Caldwell et al. 2019). Secondly, an effect assessment was performed based on existing ecotoxicological information. This information was used to determine PNECs by incorporating recent test results. Finally, PECs and PNECs were coalesced into ecological risk quotients (RQs) throughout the Vecht River network under 2 distinct water flow condition scenarios. This helps improve our understanding of the risk posed by APIs to local freshwater communities and advances the ability to evaluate and prioritize potential (local) mitigation strategies before their implementation by competent authorities (Government of The Netherlands 2019).

## MATERIALS AND METHODS

### Pharmaceuticals

Ecological risks were assessed for 8 selected APIs (Table 1). These represent only a subset of APIs detected in the Vecht River catchment (data not shown). The selection covers a wide range of consumption patterns, therapeutic classes, chemical properties, and levels of data availability (Supplemental Data).

**Table 1:** Names, Chemical Abstracts Service numbers, Anatomical Therapeutic Chemical codes, and therapeutic classes of the 8 active pharmaceutical ingredients assessed in the present study

API	CAS no.	ATC code	Therapeutic class
17α‐Ethinylestradiol^a^	57‐63‐6	G03CA01	Sex hormones
Carbamazepine^c^	298‐46‐4	N03AF01	Antiepileptics
Ciprofloxacin^b^	85721‐33‐1	J01MA02	Antibacterials
Cyclophosphamide	50‐18‐0	L01AA01	Antineoplastics
Diclofenac^a^	15307‐86‐5	M01AB05	NSAID
Erythromycin^a^	114‐07‐8	J01FA01; QJ01FA01^d^	Antibacterials
Metformin^c^	657‐24‐9	A10BA02	Antidiabetics
Metoprolol	37350‐58‐6	C07AB02	Beta‐blockers

^a^ Substance excluded from the watch list under the Water Framework Directive (Gomez Cortes et al. 2020).

^b^ Substance included in the watch list under the Water Framework Directive (Gomez Cortes et al. 2020).

^c^ Candidate substance suggested by individual member for inclusion for the next watch list under the Water Framework Directive (Gomez Cortes et al. 2020).

^d^ Substance used in human and veterinary medicine.

API = active pharmaceutical ingredient; CAS = Chemical Abstracts Service; ATC = Anatomical Therapeutic Chemical.

### Case study area

The study area comprises the catchment area of the German and Dutch transboundary Vecht River, a tributary of the Dutch IJssel River. The area is under the influence of diverse anthropological stressors (e.g., treated wastewater emissions, water level control via pumps and locks; Lulofs and Coenen 2007; Wöhler et al. 2020; Lämmchen et al., 2021). The catchment extends over an area of approximately 6100 km². The total length of the Vecht River itself amounts to 167 km, of which approximately 107 km are located in Germany.

**Figure 1:**
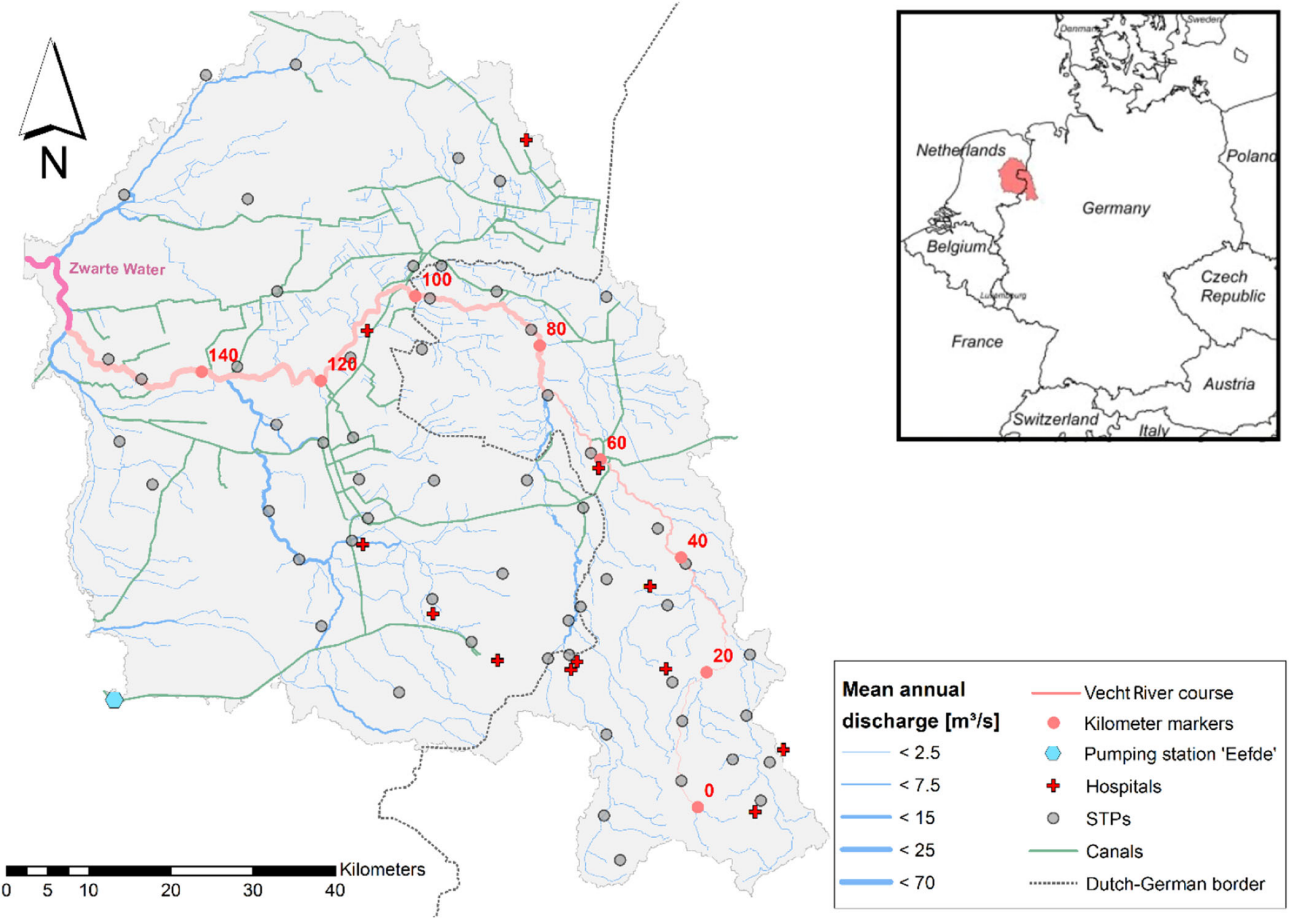
Vecht River basin. Kilometer markers start at the confluence of the Vecht tributaries Burloer Bach and Rockeler Mühlenbach. STPs = sewage treatment plants.

The German part of the catchment is located in the western part of Lower Saxony and in small sections of North Rhine‐Westphalia, comprising the smaller part of the total catchment area with a share of 1800 km² (Figure 1). In Germany, the Vecht is a medium‐sized river (long‐term annual average flow of approximately 18.5 m³/s at the German–Dutch border) with many small tributaries, for example, the Steinfurter Aa and the Dinkel. The river system is still in an almost natural state in the German regions (Lulofs and Coenen 2007), with a few canals (e.g., Ems‐Vecht Canal and the Nordhorn‐Almelo Canal) having negligible influence on river flow. The German part is less densely populated (160 inh/km^2^) than the Dutch part (260 inh/km^2^) because only small towns such as Nordhorn and Gronau (≈50 000 inhabitants) are located in this area. In total, emissions from approximately 400 000 inhabitants connected to 25 sewage treatment plants (STPs) enter the German Vecht. In addition, the wastewater of 6 hospitals with approximately 1200 beds in total is treated by the STPs.

Approximately 4300 km² of the transboundary catchment is located in The Netherlands, namely in the provinces of Overijssel and Drenthe. This part of the catchment is highly influenced by anthropogenic activities, which resulted in canals, sluices, pumps, and river straightening (Lulofs and Coenen 2007; Lämmchen et al., 2021). Larger cities with more than 100 000 inhabitants are Enschede, Zwolle, and Emmen. In total, more than 1 000 000 inhabitants are connected to 32 STPs, as are 7 hospitals with approximately 2000 beds in total. The Zwarte Water River, a short prolongation of the Vecht River and an inflow of the Zwarte Meer Lake, was integrated into the model representation.

### Environmental exposure assessment

The GREAT‐ER model was used to predict environmental concentrations of the 8 case study APIs. The GREAT‐ER model was originally developed to predict spatially explicit stationary exposure concentrations of “down‐the‐drain” chemicals in surface waters at the catchment level (Feijtel et al. 1997). The model has been successfully applied to various chemicals in different European catchments (Hüffmeyer et al. 2009; Alder et al. 2010; Aldekoa et al. 2013; Kehrein et al. 2015). A detailed description of the functions of the model and its latest extensions can be found in Kehrein et al. (2015; Lämmchen et al., 2021). The model mainly consists of 3 components: the hydrological network, the emission model, and the fate model. The hydrological network is the centerpiece of the GREAT‐ER model. The water network is discretized into river segments with a length of up to 2 km. Each segment carries a property vector that is used to calculate the chemical's fate and concentration.

#### Exposure scenarios

The steady‐state model GREAT‐ER represents a static hydrological situation over time. Two different scenarios were set up for the hydrological network, a low‐flow condition scenario (mostly dry periods in summer) and an average‐flow condition scenario (Table 2). This allows for considering the effect of the change of flow directions in some parts of the network during dry periods caused by pumping systems in the Dutch canals (Lämmchen et al., 2021).

**Table 2:** Characteristics of the simulated low‐flow and average‐flow condition scenarios

	Dry summer scenario	Average condition scenario
Applicability	Dry periods without rainfall between June and September	Humid periods throughout the year
Flow rate at the border (m³/s)	2.82	18.5
Flow rate at the Zwarte Water (m³/s)	11.31	63.45
Flow velocity at the border (m/s)	0.22	0.6
Flow velocity at the Zwarte Water (m/s)	0.33	0.85
Pumping activity	Yes	No
Pumping description	120 d/yr between March and October (Netherlands)	—
Pump power “Eefde” (Twente Canal; m³/s)	1.6 (mean), 14 (maximum)	—
Changes in flow direction	Yes	No
	Twente Canal, Zijkanaal Almelo, Canal Almelo‐De Haandrik, and several emerging smaller canals	—

#### Model parameterization

A key input parameter is the consumption of APIs in the investigated area. It is well known that consumption patterns sometimes vary between countries and regions, which holds true for some of the investigated compounds in The Netherlands and Germany (Table 3). Regional sales data for the Vecht catchment from 2017 were acquired for the regions in Germany and The Netherlands from IQVIA Commercial GmbH & Co. OHG (IQVIA, Frankfurt am Main, Germany, unpublished data) and the Dutch Foundation for Pharmaceutical Statistics (SFK, The Hague, Netherlands, unpublished data) at the postcode level (Supplemental Data, Table S1). Data include pharmacy sales but not the amount dispensed in hospitals, nursing homes, or by general practitioners. Drugs sold over the counter are included in the German data set but not in the Dutch data set. Annual prescription data were divided by the population number in the respective area, resulting in average per‐capita consumption values (Supplemental Data, Table S1).

**Table 3:** Relative percentage differences of prescribed per‐capita pharmaceutical masses in the Vecht River basin regional area, Germany and The Netherlands

	Regional‐to‐national (%)		Germany‐to‐Netherlands (%)	
	Germany	Netherlands	Within region	Between countries
17α‐Ethinylestradiol	12	–2	–75	–78
Carbamazepine	–4	16	2	25
Ciprofloxacin	9	10	27	28
Cyclophosphamide^a^	33	n.a.	n.a.	n.a.
Diclofenac	–2	–2	183	183
Erythromycin	56	–13	1594	853
Metformin	–14	6	–26	–9
Metoprolol	–8	22	–10	20

^a^ Cyclophosphamide is restricted to clinical use. The Dutch Foundation for Pharmaceutical Statistics only collects domestic pharmaceutical consumption. Therefore, no cyclophosphamide is recorded for The Netherlands.

n.a. = not applicable.

The contribution of hospitals was considered in terms of a per‐bed application. This number was different for the 2 countries and was estimated from available prescription data of selected hospitals on both sides of the border (Supplemental Data, Table S1).

Emission loads into the sewer system of an STP were estimated by multiplying the per‐capita and per‐bed application rates with the number of connected inhabitants or hospital beds, respectively. Because most APIs are metabolized after uptake, only the excreted fraction was considered (Supplemental Data, Table S2). Metabolites such as glucuronides, which react back to the parent compound after release into the sewer, were also included (Heberer and Feldmann 2005).

A fraction of the excreted amount is removed during wastewater treatment in STPs. In the Vecht River catchment, all STPs are equipped with biological treatment with no additional stage for further elimination of micropollutants such as ozonation, ultrafiltration, or activated charcoal filtration. Although removal efficiencies may depend on the specific operating conditions (Verlicchi et al. 2012), equal removal efficiency for each API in all STPs was assumed.

From a comprehensive literature search, removal efficiencies determined in STPs equipped with biological treatment collected as composite samples (>24 h) were used to calculate median values for the model simulations (Supplemental Data, Table S4).

The estimated load in treated effluents is routed into the receiving rivers at the respective discharge points. Cumulated loads are propagated through the river network and used to estimate spatially resolved API concentrations (PECs) through division of the load by the respective river flow rate. In addition, the fate model accounts for physicochemical loss processes such as (bio)degradation, sedimentation, and photolysis. Degradation via hydrolysis and dissipation via volatilization were not accounted for because of their negligible influence on APIs (Patel et al. 2019). A detailed overview of the parametrization of in‐stream processes is provided in Supplemental Data, Table S5.

#### Model evaluation

**Figure 2:**
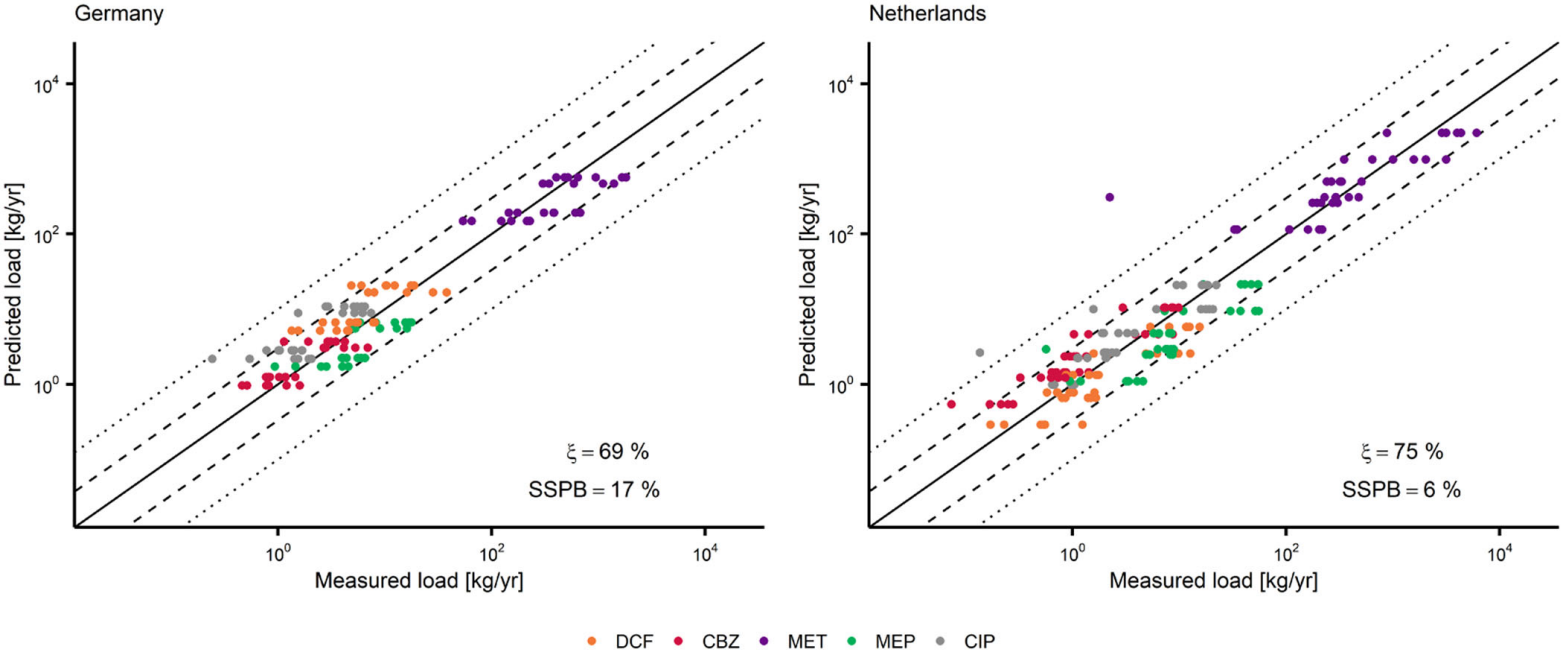
Predicted and measured sewage treatment plant (STP) influent loads of 5 pharmaceuticals (with quantification frequency >90%) in German STPs (*n* = 125) a*n*d Dutch STPs (*n* = 170). Dashed lines indicate the 1:3 and 3:1 ratios; dotted lines indicate the 1:10 and 10:1 ratios. SSPB = symmetric signed percentage bias; DCF = diclofenac; CBZ = carbamazepine; MET = metformin; MEP = metoprolol; CIP = ciprofloxacin.

**Figure 3:**
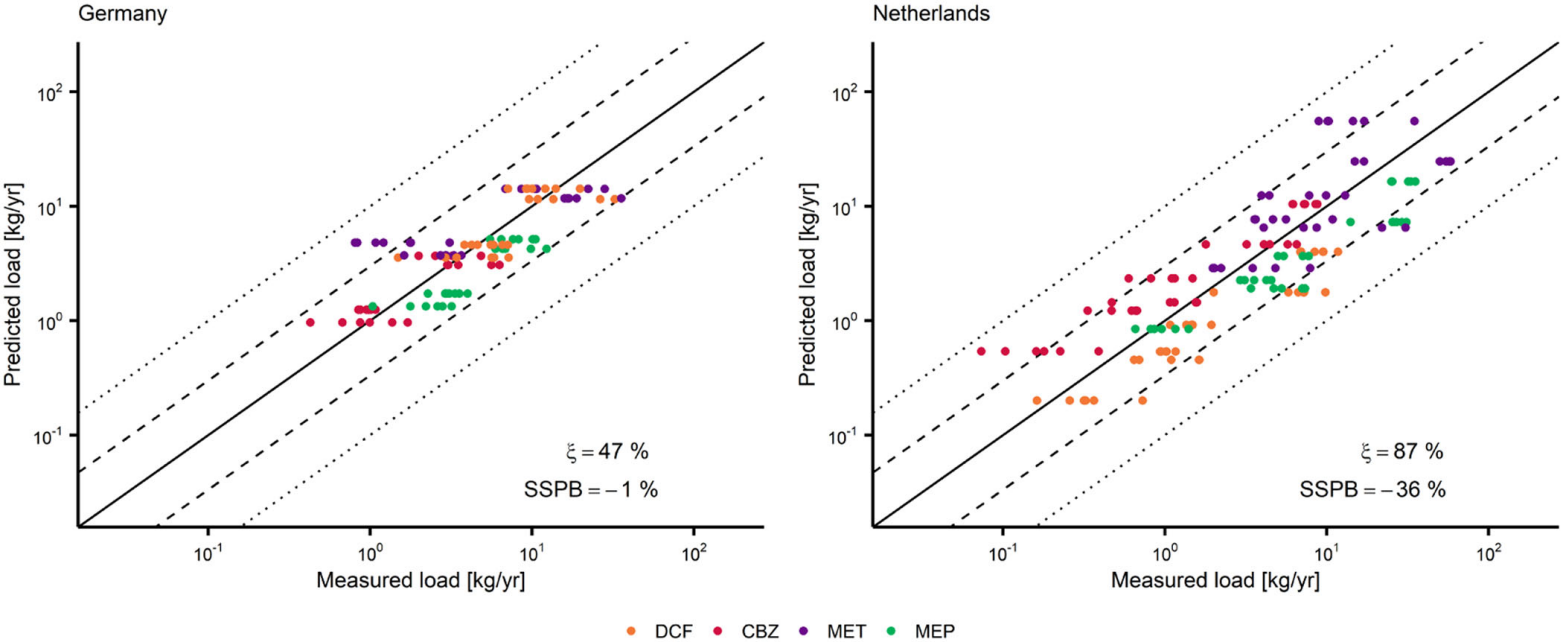
Predicted and measured sewage treatment plant (STP) effluent loads of 4 pharmaceuticals (with quantification frequency >90%) in German STPs (*n* = 100) a*n*d Dutch STPs (*n* = 132). Dashed lines indicate the 1:3 and 3:1 ratios; dotted lines indicate the 1:10 and 10:1 ratios. SSPB = symmetric signed percentage bias; DCF = diclofenac; CBZ = carbamazepine; MET = metformin; MEP = metoprolol.

The model performance was evaluated stepwise by comparison of simulation results with monitoring data for selected APIs in STP influents and effluents as well as at selected river sites (Figures 2 and 3). A comprehensive description of the sampling strategy is provided elsewhere (Heijnsbergen et al., unpublished manuscript). A brief overview and details for the chemical analysis are provided in Supplemental Data, S1.1 and S1.2.

Two model performance quantitative measures were applied: median symmetric accuracy (ξ) and the symmetric signed percentage bias (SSPB; Morley et al. 2018),

(1) $$r_{i}=\frac{x_{i,\mathrm{pred}}}{x_{i,\mathrm{meas}}}$$

(2) $$\xi \;(\%)=100\times (e^{M(|\;\ln\;r_{i}|)}-1)$$

(3) $$\text{SSPB}\;(\%)=100\times (e^{\left|(M(\ln\;r_{i})\right|}-1)\times sgn(M(\ln\;r_{i}))$$

where $r_{i}$ is the ratio of the predicted/measured pair (e.g., loads), $x_{i,\mathrm{pred}}$ is the predicted value, $x_{i,\mathrm{meas}}$ is the corresponding value from the measurement data, M is the median function, sgn is the sign function, and i is the index within a subgroup of all predicted/measured pairs for a single compound, scenario, country, sampling site, or a combination of these.

The median symmetric accuracy (Equation 2) is a measure of central tendency that is robust to the presence of outliers and resistant to data spanning several orders of magnitude. For the scope of the present study, we consider ξ values up to 100 and up to 200% as indicative of “good agreement” and “acceptable agreement” between measurements and predictions, respectively. Values of ξ > 200% indicate “poor agreement” between measurements and predictions. A ξ = 100% indicates that the median of the absolute ratios (|*r*_*i*_|) is 2 (i.e., 50% of predicted values deviate from measured values by less than a factor of 2). The symmetric signed percentage bias (Equation 3) can be interpreted similarly to a mean percentage error, but it penalizes underestimation and overestimation equally. Positive values indicate a tendency to overestimate predictions, whereas negative values indicate a tendency to underestimate predictions. In the present study, absolute values of SSPB up to 50, 100, and 200% were considered as an indication of “small,” “medium,” and “large” overestimations or underestimations, respectively. Absolute values >200% were considered “very large” overestimations/underestimations. An SSPB = –50% indicates that the median of relative ratios (*r*_*i*_) is 50% lower in the predictions compared to measured data. This implies that 50% of the predicted values underestimate the measurements by at least a factor of 1.5.

Predictions of STP emissions were evaluated on a load‐based approach. Measured concentrations in STP influent and effluent were multiplied with the annual discharge of the corresponding STP and compared to model predictions. The APIs with a quantification frequency <90% were evaluated semiquantitatively. Concentrations below the limits of quantification (LOQ) were processed as LOQ in the evaluation approach because they are expected to be close to the LOQ value as a result of the high quantification frequency.

Surface water PECs were evaluated using the “benchmark” concept, according to Kunkel and Radke (2012), with which concentrations of individual APIs are normalized to the concentration of a conservative tracer or reference. Thereby, river flow variations can be excluded from the evaluation process. Carbamazepine was selected as the conservative reference compound because of its persistence in the environment (Aminot et al. 2016). Benchmark ratios from the monitoring data could only be calculated if the concentration of the reference (carbamazepine) and that of the respective target API were above the LOQ. To provide a reliable baseline for this approach, predicted carbamazepine concentrations were evaluated by comparison with measured concentrations (Supplemental Data, S1.3).

### Environmental effect assessment

#### Search strategy

Aquatic ecotoxicity data were compiled without restrictions from the following databases: ECOTOX Knowledgebase (US Environmental Protection Agency 2019), e‐toxBase (Posthuma et al. 2019), Wikipharma (Molander et al. 2009), FASS (Trade Association for the Research‐Based Pharmaceutical Industry in Sweden 2019), iPiESum (Innovative Medicines Initiative 2019), and the EU WRC report (Johnson and Harvey 2002). To further supplement collected data, a literature review was performed by searching the Web of Science platform in March 2019 (Supplemental Data, Table S11). The search was restricted to publications from 2016 or later to capture information not covered by the other sources. The search returned 233 publications that were fully assessed.

#### Data extraction and harmonization

All relevant toxicological information referring to the 8 APIs of interest was extracted from the databases. Additional toxicity data were extracted from 40 publications identified in the public literature search. The following relevant information was extracted and compiled: substance name, Chemical Abstracts Service number, taxon, species, life stage and living compartment of the species tested, toxic effect, exposure type, exposure duration, endpoint type, and endpoint value. This process resulted in an initial database with a total of 11 029 entries (Table 4). The data were harmonized to guarantee their consistency and usability, which included harmonizing the names of species, toxic effects, exposure duration and types, end points, and concentration units (Supplemental Data, S2).

**Table 4:** Number of ecotoxicological data entries per source in the database compiled in the present study

Source	Entries
ECOTOXbase	6510
Wikipharma	2802
e‐toxBase	779
Literature	455
iPiESum	270
EU WRC report	140
FASS	74

#### Data selection

The information in the database was filtered to obtain only relevant data for analysis. Only aquatic or semiaquatic species were included. Entries referring to terrestrial species, communities, sediment tests with no reported water concentrations, or in vitro tests or with no single species name specified were excluded from the analysis. Only population‐relevant endpoints were selected, that is, those which can adversely affect an organism's survival, ability to maintain its population numbers, reproduction, development, growth, or behavior. Effect endpoints with right/left‐censored values (i.e., <, >, ≤, ≥) were excluded. Similarly, identical effect entries from the same original source were excluded. Toxicity values for the same species and endpoint but originating from different studies were aggregated by taking the geometric mean weighted by the number studies with identical endpoints. This resulted in a final database containing 169 effect values usable for further analysis.

#### Data reliability

To ensure that we only included reliable and relevant toxicity studies in our assessment, all studies were assigned a criteria for reporting and evaluating ecotoxicity data (CRED) score (Moermond et al. 2016a). Studies classified as unreliable (R3), unassignable reliability (R4), irrelevant (C3), or unassignable relevance (C4) were excluded from further analysis. We preferably used classification scores from official sources, such as the Dutch National Institute for Public Health and the Environment and the German Environment Agency. Alternatively, the authors (D.J. Duarte, R. Oldenkamp, and A.M.J. Ragas) independently assigned CRED scores to critical studies according to Moermond et al. (2016a) after evaluating and discussing any inconsistencies (Supplemental Data, Table S12). Exceptionally, experiments on 17α‐ethinylestradiol without classifications from official sources were not evaluated because of the extensive number of studies and additional complexity of assessing the quality of ecotoxicological studies testing endocrine‐disrupting effects; such an exhaustive assessment was considered beyond the scope of the present study.

#### PNECs

Two extrapolation methods for the derivation of chronic PNEC values are typically used in effect assessment: the species sensitivity distribution (SSD) and the assessment factor (European Commission 2000, 2006). According to European Union guidance, an SSD‐based PNEC requires a considerable amount of data covering at least 3 trophic levels (primary producers, plant‐eating animals, and predators), at least 8 taxonomic groups, and at least 10 effect values (one per species per substance). As for the assessment factor approach, at least one short‐term median effective concentration from each of the 3 trophic levels is the minimum requirement. Because the final database did not satisfy SSD data requirements for the derivation of PNECs, only the assessment factor approach was implemented (Supplemental Data, Table S15). The estimation of a PNEC using this deterministic approach was done by dividing the lowest effect concentration by an assessment factor, according to the European Union Water Framework Directive guidance for deriving aquatic EQSs (European Commission 2018). Depending on the available data, this factor varies between 10 and 1000. A collection of PNEC estimates from the literature and other sources was gathered for comparison (Supplemental Data, Table S16).

### Ecological risk

Predicted environmental concentrations and PNECs were used to calculate a site‐specific RQ associated with each API following the equation,

(4) $${\text{RQ}}_{s,p}=\frac{{\text{PEC}}_{s,p}}{{\text{PNEC}}_{p}}$$

where ${\text{RQ}}_{s,p}$ is the RQ at site s for pharmaceutical p, ${\text{PEC}}_{s,p}$ (µg/L) is the PEC at site s for pharmaceutical p, and ${\text{PNEC}}_{p}$ (µg/L) is the PNEC for pharmaceutical p.

Evaluation of PNEC exceedance was performed based on the total river volume in the Vecht catchment and for the cumulated flow length of the water bodies in the catchment. Because of the steady‐state assumption of the GREAT‐ER model, a constant water volume in the system is assumed for each of the scenarios.

Pharmaceutical mixture risk was calculated based on the conservative approach of concentration addition following the equation,

(5) $${\text{RI}}_{s}=\overset{n}{\underset{i=1}{\sum }}{\text{RQ}}_{s,p}$$

where ${\text{RI}}_{s}$ is the risk index of a pharmaceutical mixture at site s, ${\text{RQ}}_{s,p}$ is the risk quotient at site s for pharmaceutical p, i is the summation index, and n is the total number of APIs. The concentration addition approach tends to overestimate the mixture risk of dissimilarly acting substances because it assumes a similar noninteractive mode of action of all mixture components. However, there is growing consensus on the pragmatic and precautious utility of this approach in aggregating risks of mixture components (European Commission 2012; Backhaus 2016; Posthuma et al. 2018; Hernandez et al. 2019; Kienzler et al. 2019).

## RESULTS AND DISCUSSION

### Predicted surface water concentrations

**Figure 4:**
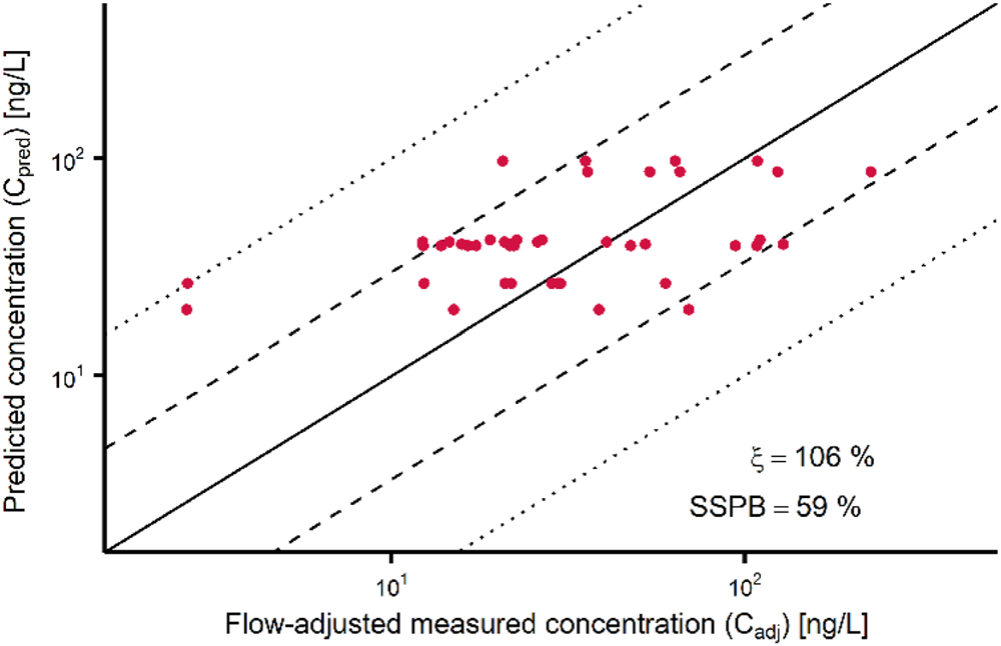
Comparison of predicted and measured carbamazepine concentrations in the Vecht catchment (*n* = 46) at monitoring sites where reliable gauging data of the corresponding sampling day were available (i.e., no change in flow direction, resulting in net flow rates of 0 m³/s). Measured concentrations were adjusted to the flow rate used in the simulations. Dashed lines indicate the 1:3 and 3:1 ratios; dotted lines indicate the 1:10 and 10:1 ratios. SSPB = symmetric signed percentage bias.

Predicted carbamazepine concentrations were evaluated to provide a reliable baseline for the benchmark approach (Supplemental Data, S3). Because carbamazepine is consumed equally throughout the year, evaluation can be performed using all data without differentiation into the 2 exposure scenarios (see above, *Exposure scenarios*). Figure 4 shows an acceptable overall agreement between PECs and MECs (ξ = 106%), with a tendency to being rather overestimated (SSPB = 59%). Approximately 80% of the PEC and MEC data differ by less than a factor of 3, so we conclude that carbamazepine provided a valid baseline for the application of the benchmark approach (Supplemental Data, Figure S3).

**Figure 5:**
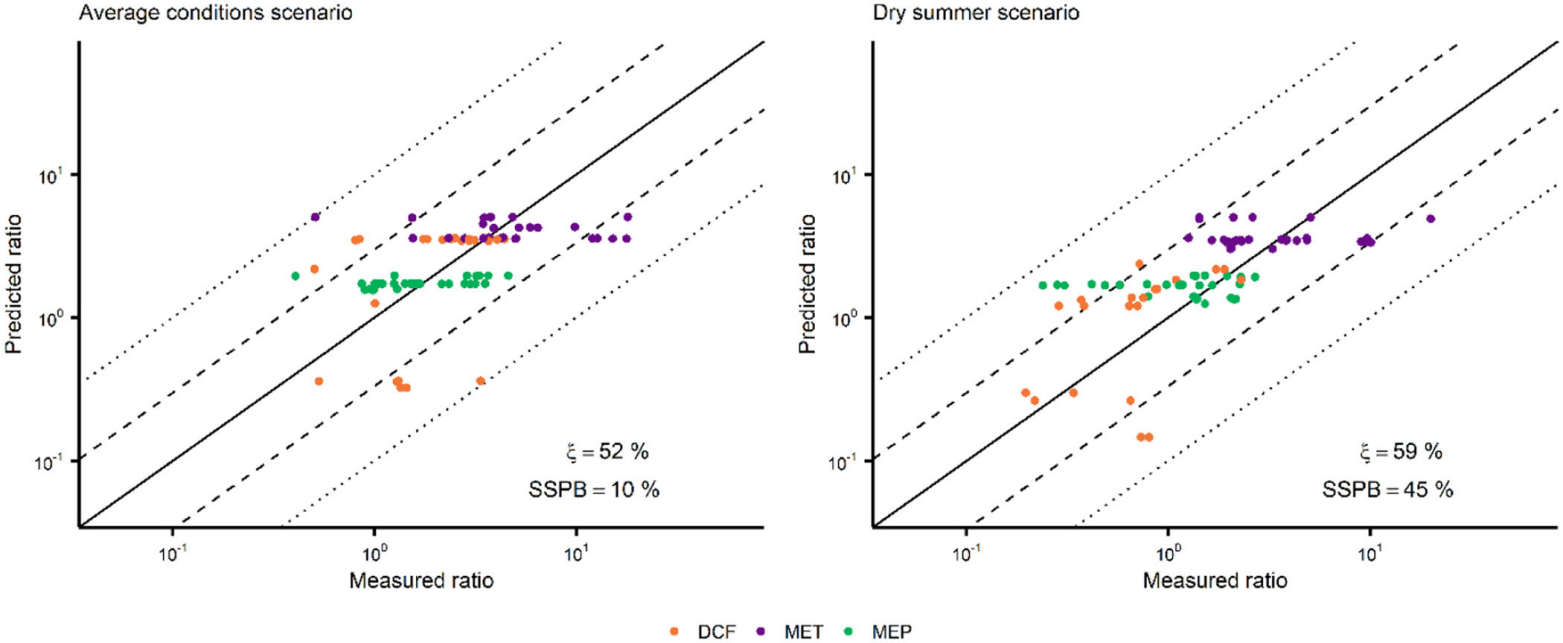
Predicted and measured benchmark ratios of 3 pharmaceuticals at monitoring sites in the whole Vecht River catchment (average condition scenario *n* = 80, dry summer scenario *n* = 81). Dashed lines indicate the 1:3 and 3:1 ratios; dotted lines indicate the 1:10 and 10:1 ratios. SSPB = symmetric signed percentage bias; DCF = diclofenac; MET = metformin; MEP = metoprolol.

The quantification frequency of erythromycin and ciprofloxacin in the river samples was <10%. Cyclophosphamide and 17α‐ethinylestradiol were not analyzed at all because of the expectation of very low concentrations far below the LOQ. Because all predicted concentrations of these compounds were below the LOQ, qualitative agreement is given. Diclofenac, metformin, and metoprolol concentrations were evaluated separately for the 2 exposure scenarios because of obvious seasonal differences (see above, *Exposure scenarios*). Predicted and measured benchmark ratios agreed well for both the average condition scenario (Scn_AC_; ξ = 52%, SSPB = 10%) and the dry summer scenario (Scn_DS_; ξ = 59%, SSPB = 45%), with approximately 80% within the range of a factor of 3 (Figure 5).

Based on the successful model evaluation of PECs, simulations for the entire Vecht River catchment were performed. In the Scn_AC_, metformin, metoprolol, and carbamazepine had the highest PECs at watercourses affected by upstream STPs, with median concentrations of 0.19 (0.01–3.03), 0.07 (2 × 10^–3^–1.44), and 0.043 (2 × 10^–3^–0.84) µg/L, respectively. Similarly, the highest median PECs in the Scn_DS_ were 0.57 (0.01–19.43), 0.25 (4 × 10^–3^–4.08), and 0.18 (0.01–2.36) µg/L for metformin, metoprolol, and carbamazepine, respectively. The preceding median, minimum, and maximum PEC values exclude river segments with a PEC of zero. In previous studies, these APIs have been predicted or measured at similar concentration ranges in Dutch (Oosterhuis et al. 2013; Moermond et al. 2020) and German (Scheurer et al. 2009; Meyer et al. 2016; Dusi et al. 2019) surface waters. Although metformin is effectively transformed into guanylurea during wastewater treatment (Oosterhuis et al. 2013), it exhibited the highest PEC among the investigated APIs. This is a consequence of the high consumption of metformin (twelfth highest defined daily dosage [DDD] and seventeenth most frequently used in The Netherlands; Dutch National Health Care Institute 2020) and its relatively high excretion rate. The lowest PECs in watercourses affected by STP effluents were exhibited by 17α‐Ethinylestradiol and cyclophosphamide, with median concentrations in Scn_AC_ of 0.02 (3 × 10^–4^–0.82) and 0.37 (0.01–9.64) ng/L, respectively. As for Scn_DS_, the concentrations for 17α‐ethinylestradiol and cyclophosphamide were estimated at 0.05 (2 × 10^–4^–0.99) and 1.17 (2 × 10^–4^–756.98) ng/L, respectively. These results were in line with the low consumption volumes of these APIs, despite a considerable fraction being excreted.

**Figure 6:**
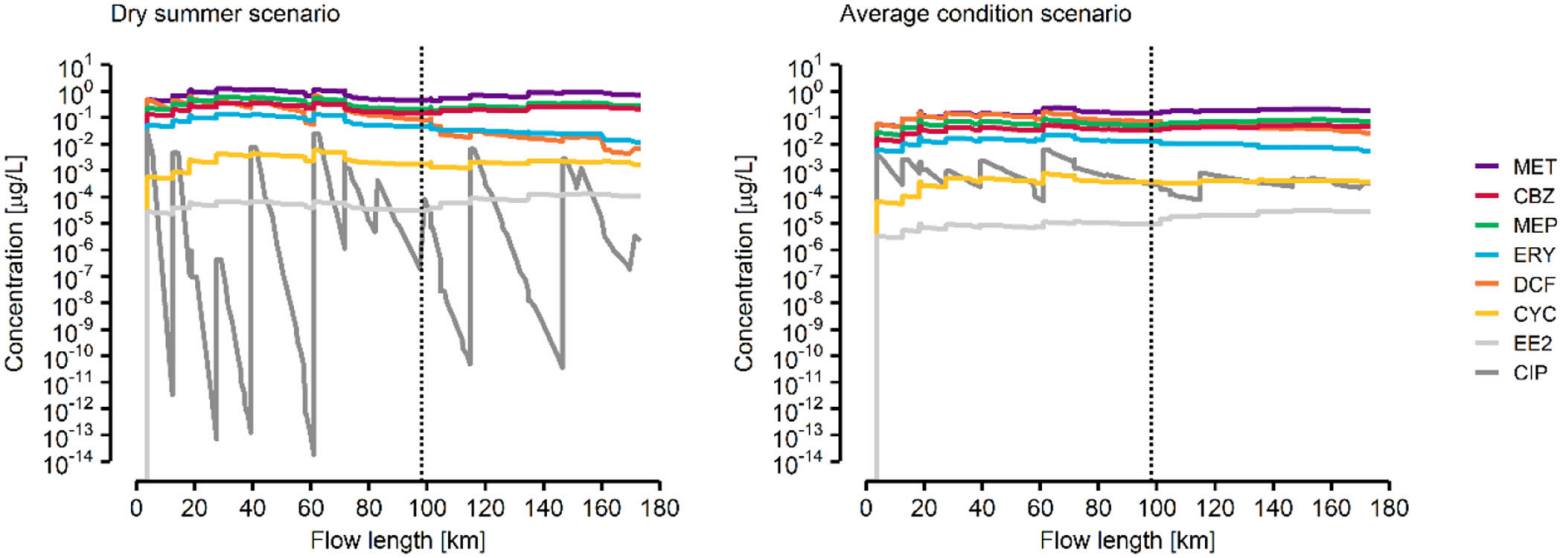
Predicted environmental concentrations of pharmaceuticals in the Vecht River main stream. The vertical black dashed line indicates the Dutch–German border. MET = metformin; CBZ = carbamazepine; MEP = metoprolol; ERY = erythromycin; DCF = diclofenac; CYC = cyclophosphamide; EE2 = 17α‐ethinylestradiol; CIP = ciprofloxacin.

Concentration profiles of the Vecht River main stream are displayed in Figure 6 for the 8 APIs in the 2 exposure scenarios. The factors that cause differences in the PEC profiles observed along the main stream can be manifold and API‐dependent. Erythromycin's low PECs in the Dutch regions coincide with the Dutch population's lower consumption patterns compared with their German counterparts. Persistent substances which are equally consumed on both sites of the border, such as carbamazepine, show higher PECs in Dutch regions because of the higher population density. Dilution ratios of treated effluent after entering the river system are lower if more people are connected to rivers with comparable flow rates. The effect of dilution is also clearly visible in the PEC profiles of the 2 scenarios: dilution in Scn_DS_ is approximately 10 times lower than in Scn_AC_. Lower flow rates lead to higher residence times and lower water levels in the river system, resulting in a larger influence of dissipation processes in Scn_DS_ than in Scn_AC_. As a result, predicted summer concentrations of most APIs (17α‐ethinylestradiol, carbamazepine, cyclophosphamide, erythromycin, metformin, and metoprolol) were on average a factor of 4 to 6 times higher than in Scn_AC_. Among the APIs studied, ciprofloxacin was the compound most susceptible to dissipation processes, namely via direct photolysis, resulting in drastically lower PECs in Scn_DS_ than in Scn_AC_. Diclofenac is also prone to direct photolysis. This in combination with lower consumption rates in The Netherlands helps explain the low PECs downstream of the border in the Scn_DS_ compared with Scn_AC_.

### PNECs

**Figure 7:**
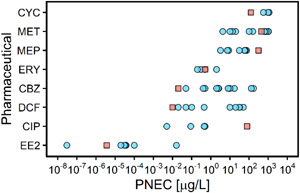
Predicted‐no‐effect concentrations (PNECs) from the literature and derived in the present study. Salmon‐colored squares indicate the PNEC values derived in the present study. Light blue points indicate unique PNEC values found in the literature. CYC = cyclophosphamide; MET = metformin; MEP = metoprolol; ERY = erythromycin; CBZ = carbamazepine; DCF = diclofenac; CIP = ciprofloxacin; EE2 = 17α‐ethinylestradiol.

In the environmental effect assessment, there was a clear disparity in data availability for different substances. The lowest chronic PNEC was exhibited by 17α‐Ethinylestradiol (3.6 × 10^–6^ µg/L) and metformin the highest (440 µg/L). We revised existing chronic PNECs of the 8 APIs, including for diclofenac (0.01 µg/L), carbamazepine (0.02 µg/L), and cyclophosphamide (125 µg/L; Figure 7; Supplemental Data, Table S15), which were 2, 2.5, and 4.5 times lower than the lowest PNECs reported previously in the literature or regulatory documents (Supplemental Data, Table S16). These lower PNECs give cause for concern regarding the environmental impact of these APIs and indicate the need to revise proposed EQSs for these APIs. For metoprolol and ciprofloxacin, the PNECs estimated in the present study were 310 and 78 µg/L, which are 5 and 156 times the highest PNECs found in the literature, respectively. It should be stressed that any PNEC can be strongly affected by the accessibility of effect data, the thoroughness of the search, and the quality assessment procedure (Henning‐de Jong et al. 2009; Oelkers 2020). This is illustrated by a suggestion we received from one of the anonymous reviewers, that is, to include the study of Ebert et al. (2011) in the derivation of the PNEC for ciprofloxacin. This is a critical study underlying the low ciprofloxacin PNEC of 0.089 µg/L listed in Supplemental Data, Table S16, yet it was not retrieved from any of the sources used in the present study. It explains the large difference in derived PNECs for ciprofloxacin observable in Figure 7 and illustrates more generally that PNECs and risk assessment outcomes based on the assessment factor approach are very sensitive to the effect data included in the assessment. Indeed, the differences in PNECs for the same API derived by different agencies and assessors range from a factor of 10 to almost 10^6^ (Figure 7). Keeping this range in mind, it is defendable to use an RQ of 0.1, or even smaller, as a potential indicator of risk and as a trigger to critically review and potentially improve the assessment procedure. To account for uncertainty in the derivation of PNEC values, an assessment factor of 50 was applied to diclofenac and 17α‐ethinylestradiol, whereas an assessment factor of 10 was applied to carbamazepine, ciprofloxacin, cyclophosphamide, erythromycin, metformin, and metoprolol. The use of a relatively low assessment factor (instead of 100 or 1000) suggests that the PNECs derived in the present study are not overly conservative.

### Aquatic ecological risk

#### Single‐substance assessment

In the present study, RQ < 0.1 indicates a reason for no concern in terms of chemical pollution, 0.1 < RQ  ≤  10 indicates a potential reason for concern, and RQ > 10 suggests a reason for serious environmental concern. The specific boundary value(s) that qualifies as a “reason for concern” is malleable, depending on the empirical data that support it and personal values. In the present study, we chose to acknowledge the uncertainties that blur the meaning of this threshold (RQ = 1). Values of RQ > 1 can trigger follow‐up measures, via either additional ecotoxicity testing or the implementation of risk management measures (Posthuma et al. 2019; Zhou et al. 2019).

**Figure 8:**
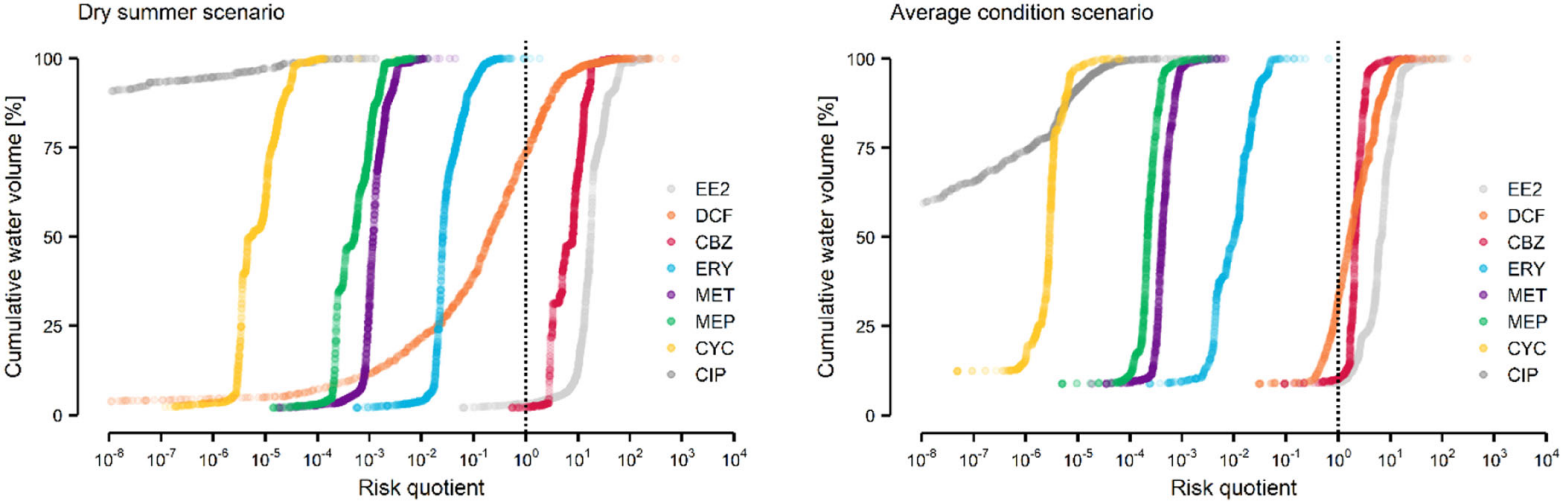
Percentage of the Vecht River catchment water volume at risk of environmental pharmaceutical pollution. Vertical black dashed line indicates the safe threshold, risk quotient = 1 (i.e., predicted environmental concentrations equal to the predicted‐no–chronic effect concentration). In the average scenario, ciprofloxacin's risk quotients are <10^–8^; thus, they are not depicted. Each point depicts the relative water volume of a segment of ≤2 km. In the dry summer scenario, concentrations of ciprofloxacin <10^–8^ are also not depicted. EE2 = 17α‐ethinylestradiol; DCF = diclofenac; CBZ = carbamazepine; ERY = erythromycin; MET = metformin; MEP = metoprolol; CYC = cyclophosphamide; CIP = ciprofloxacin.

In the present study, the PECs of 5 APIs were below their safe thresholds (PNECs). However, the PECs systematically exceeded PNECs in ascending order for diclofenac, carbamazepine, and 17α‐ethinylestradiol (Figure 8). This observation holds for the average and dry summer scenarios, although risks were considerably higher in summer because of reduced dilution under dry weather conditions. Diclofenac, carbamazepine, and 17α‐ethinylestradiol exceeded the safe PNEC threshold in at least 68 to 91% and 26 to 98% of the Vecht River catchment surface water volume during average conditions and dry summer conditions, respectively. In terms of the total flow length of all water bodies, the same APIs exceeded their PNECs in 31 to 38% and 24 to 53% during average conditions and dry summer conditions, respectively (Supplemental Data, Figure S4). In the average condition scenario, ciprofloxacin, cyclophosphamide, erythromycin, metformin, and metoprolol do not pose a concerning risk to the aquatic life (i.e., 93 to 100% of the water volume had RQ ≤ 0.1). In the dry summer scenario erythromycin showed concerning risk levels (RQ > 0.1) in 17% of the catchment's water volume.

17α‐Ethinylestradiol exhibits the highest RQs despite showing the lowest PECs overall, with 25 and 87% of the catchment water volume showing concerning risk levels (RQ > 10) in the average and summer scenarios, respectively (Supplemental Data, Table S17). In the Dutch municipality of Hengelo, 17α‐ethinylestradiol showed a local risk of serious concern under average conditions in a small brook (RQ_ScnAC_ = 144), whereas under dry summer conditions the risks were highest at local canals (<2 km) routing STP effluents into larger streams and canals, for example, Bornse Beek (RQ_ScnDS_ ≤ 274). This synthetic hormone has been shown to particularly interfere with the endocrine system of fish and amphibian species, affecting their development, reproduction, growth, and, ultimately, ability to sustain a healthy population (Supplemental Data, Table S15). Eight of the 10 most sensitive species to ethinylestradiol identified in the present study are fish. Notably, *Gobiocypris rarus* (commonly known as rare minnow), a fish species endemic to China, is the most sensitive species (Zha et al. 2008). However, *Rutilus rutilus* (commonly known as roach) is a fish native to most European freshwaters including the Vecht River and is similarly sensitive (Lange et al. 2009). One study assessed the effect of wastewater estrogen exposure on roach population density in 2 English rivers over the span of a decade, finding no noticeable declines (Johnson and Chen 2017). Another study analyzed the results of fish samples over a period of 2 decades in German rivers and found a decrease in fish population density, although it could not attribute it to chemical pollution (Teubner et al. 2019). To our knowledge, there are currently no indications that the roach is subject to adverse effects in the Vecht River basin. Nonetheless, the results of the present study support the use of more sensitive analytical techniques combined with accurately modeled hotspots of estrogen pollution and fish species in the Vecht River basin, including the roach. Furthermore, considering that the majority of the catchment was predicted to be liable to serious environmental risk, chronic effects could be triggered because continuous exceedance of an RQ of 1 is very likely under the simulated scenarios. At catchment locations, these exceedances can vary substantially, which can provide an opportunity for motile organisms to avoid unfavorable conditions or endure them for shorter exposure periods.

Carbamazepine exhibited the second highest RQs, with 90% of the catchment water volume showing concerning risk levels (RQ_ScnAC_ > 0.1; Supplemental Data, Table S17). Throughout the catchment, carbamazepine showed its highest risk (RQ_ScnDS_ = 118, RQ_ScnAC_ = 42) in a 7‐km tributary segment under high‐effluent influence, located in the German municipality of Bad Bentheim. Carbamazepine causes a variety of toxicological effects at different taxonomic levels. The most sensitive species include the insect *Stenomena* sp. (Jarvis et al. 2014), the crustacean *Daphnia similis* (Chen et al. 2019), the algae *Chaetophora* sp. (Jarvis et al. 2014), and the fish *Pimephales promelas* (Thomas et al. 2012), for which carbamazepine affects behavior, reproduction ability, or population survival. It is unclear whether these species are present in the Vecht River, but given carbamazepine's diverse ecotoxicological potential, targeted monitoring of its concentration levels and the sensitive *Stenomena* sp. could help determine whether adverse effects occur under field conditions.

Diclofenac exhibited the third highest RQs, with 90% of the catchment water showing concerning risk levels (RQ_ScnAC_ > 0.1; Supplemental Data, Table S17). At the same location in the German municipality of Bad Bentheim, diclofenac showed the highest risk quotient (RQ_ScnDS_ = 754, RQ_ScnAC_ = 302). Provided the high risk at this and other locations along the Vecht River basin, toxicological effects on growth and development could be expected on fish and algae. The most sensitive species to diclofenac is the widespread invasive bivalve *Dreissena polymorpha*, which may be indicative of the vulnerability of this taxonomic rank (mollusks) and the trophic level it represents (primary consumers). These freshwater mollusks provide essential ecosystem services, are key elements of the food chain, and play a major role in removing contaminants from high volumes of water. At the regional and local scales, pharmaceutical pollution could exacerbate the impact on what is already the most threatened animal group in Europe (Cuttelod et al. 2011).

In a Dutch governmental report, carbamazepine and diclofenac have previously been identified as contaminants of environmental concern to aquatic organism in The Netherlands (Moermond et al. 2016b); and, in a revised iteration, 17α‐ethinylestradiol has also been identified as such, whereas carbamazepine was no longer of concern (Moermond et al. 2020). The revised PNECs in the present study suggest that the RQs of diclofenac and carbamazepine may be higher than anticipated (underestimated RQ).

Exceptionally, erythromycin was also marginally predicted to occur at concentrations above the PNEC in the Vecht River catchment freshwater in a typical summer season (RQ = 1.8). In the river's main stream, RQs were low (RQ < 0.1), particularly in Dutch territory because of water dilution and lower consumption. Furthermore, erythromycin's degradation in the water column is not expected to be substantial because of the limited residence time of APIs in the Vecht River main stream of 4 to 12 d for average and low‐flow conditions, respectively (Liu et al. 2019; Li and Cui 2020). However, the unaccounted veterinary use of erythromycin in the present study could elevate the risks.

Metformin does not stand out from our risk profiling. However, metformin's main metabolite, guanylurea, is found in surface waters in quantities of up to 50% of the administered parent compound (Oosterhuis et al. 2013). Because guanylurea has a lower PNEC (0.16 µg/L) than metformin itself (Caldwell et al. 2019), risk assessment of metformin should include the metabolite because it could pose a risk related to widespread metformin application. The need to consider transformation products in aquatic risk assessment has been stated by other authors (Celiz et al. 2009; Han and Lee 2017).

Overall, 17α‐ethinylestradiol, carbamazepine, and diclofenac may pose unacceptable environmental risks in at least 31% of the Vecht catchment flow length for average conditions. This risk aggravates up to 53% during summer, affecting 1483 out of 2772 km of total flow length (Supplemental Data, Figure S4). The average RQ increased consistently across APIs by approximately 10‐fold between the average and dry summer scenarios. However, the most striking changes in PEC were observed at the confluence of polluted streams, effluent‐dominated waters, or segments receiving STP effluents, with a few instances in which treated effluent discharge contributed up to 90% of the stream's volume. Other studies have also observed that proximity to STPs can more heavily influence pharmaceutical PEC than seasonality (Musolff et al. 2009; Balaam et al. 2010; Vieno and Sillanpää 2014). Because of human activity near the river source, API emissions result in residue concentrations exceeding the PNEC as early as 20 km downstream the Vecht River. In agreement with the present study, diclofenac and carbamazepine have also been predicted to display a high environmental risk in other European and international rivers (Chaves et al. 2020; Palma et al. 2020). The APIs with the highest RQs in the present study (17α‐ethinylestradiol, carbamazepine, diclofenac, erythromycin) have recently been removed from the Water Framework Directive watch list, which may lead to losing sight of their ecological impact despite their potential risk. This is also emphasized by Burns et al. (2018), who identify these substances as common top‐priority APIs. In addition, a review on the development in the field of substances of emerging concern over the previous 20 yr emphasizes the exceedance of EQSs and the need for spatially explicit risk modeling approaches (Tiedeken et al. 2017). This review further supports the usefulness of generating spatially explicit risk profiles as conducted in the present study. Similar efforts open up the possibility for stakeholders to comply with the Water Framework Directive, starting with prioritizing APIs so that more refined and locally relevant targeted risk‐management measures can be applied successfully.

#### Substance mixture assessment

In the Vecht catchment, a noticeable difference between the risk index in the average scenario and the dry summer scenario was observed (Supplemental Data, Figures S5 and S6). In the dry summer scenario, the mean risk index was estimated to be 3.4 times higher than in the average condition scenario. Likewise, the maximum risk indices were found in river segments of the Dutch municipalities of Hengelo and Coevorden under average and dry summer condition scenarios, respectively. This suggests that periods of dry, warm weather conditions in the Vecht River catchment may lead to risks to freshwater wildlife communities above the risks estimated for average weather conditions.

**Figure 9:**
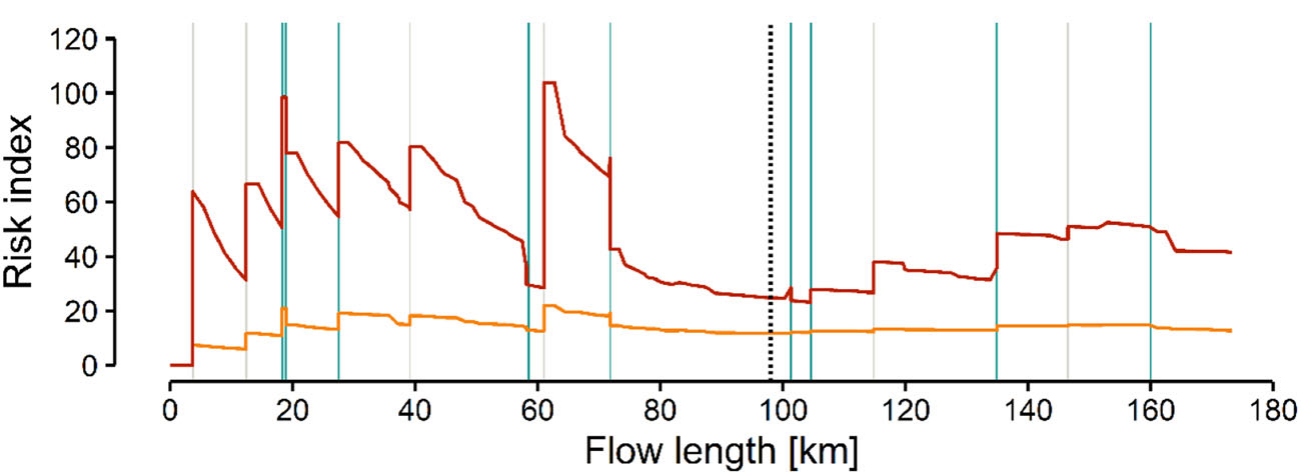
Risk index along the Vecht River main stream under typical dry summer (orange) and average weather (red) conditions. Eight pharmaceutical active ingredients are integrated in the risk indices depicted. Dashed vertical line demarks the German–Dutch border. Solid vertical lines depict sewage treatment plants (gray) and tributary confluences (turquoise).

In the Vecht River main stream (Figure 9), the predicted cumulative risk in the polluted segments (i.e., risk index > 0) ranges between 6 to 22 and 23 to 104 in the average scenario and dry summer scenario, respectively. These risk index values in the main stream are lower than observed elsewhere in the catchment (Supplemental Data, Figures S5 and S6). However, this emphasizes the sustained cumulative risk in the Vecht River's main stream, particularly driven by diclofenac in the German region and 17α‐ethinylestradiol in the Dutch region (Figure 8).

### Limitations

The present study embodies the ongoing attempt to predict API concentrations in freshwater and the associated risk of biological functional disturbance in regional ecosystems. Despite the advancements achieved, data scarcity, knowledge gaps, and procedural limitations often hamper the accuracy and significance of exposure and effect assessments. The sources of variability and uncertainty that can affect PECs and PNECs are manifold. The PEC can be affected by the excretion rate, sampling method, analytical chemistry technique, unaccounted point and diffuse emission sources, in‐sewer (bio)transformation, disposal of unused medicine in the toilet, or household wastewater (van Nuijs et al. 2015). For example, there are uncertainties linked to the German consumption rate of erythromycin, which seems to have been overestimated. Furthermore, erythromycin and ciprofloxacin PECs are associated with higher uncertainties because these were not sufficiently detected in the Vecht water system to allow for a corroboration with measurements. Similarly, the accuracy of model predictions for cyclophosphamide and 17α‐ethinylestradiol could not be firmly determined because of analytical limitations. Indeed, concentrations of these APIs in surface water were often below their limits of detection and quantification. This is particularly important for assessing the risks associated with substances like 17α‐ethinylestradiol because of its very low safe PNEC. Therefore, under such analytical limitations, the crucial contribution of predictive models is self‐evident. The sensitivity of derived PNECs to data availability (e.g., effect studies that are missed, differently quality‐assessed, or newly performed) is a typical feature of the assessment factor method. The alternative SSD method is less affected by this phenomenon because it uses the 5th percentile of the cumulative distribution function. As such, the sensitivity of PNECs to data availability also partly relates to the strict criteria on data availability that the European Union set for applying SSDs.

## CONCLUSION

The present study achieved 3 main goals: 1) estimation of API surface water concentrations using the GREAT‐ER model in the Vecht catchment; 2) derivation of new safe ecological threshold concentrations for 8 APIs, of which 3 were the lower than found in the literature; and 3) the creation of detailed, spatially explicit ecological risk profiles of APIs in a transboundary (sub‐)catchment under 2 different seasonal scenarios. The exceedance of the acceptable ecological risk threshold in the Vecht River was found to be mainly driven by 17α‐ethinylestradiol, diclofenac, and carbamazepine. These substances are among the most consumed APIs in The Netherlands. 17α‐Ethinylestradiol predominantly contributed to the aggregated risk profile and systematically exceeded the PNEC by at least one order of magnitude. This substance is the API with the twenty‐third highest DDD and has seen a 4% increase from 2018 to 2019 (Dutch National Health Care Institute 2020). This prospect emphasizes the need for better pharmaceutical emission reduction strategies (e.g., wastewater treatment technology, hotspot analysis, and preventive health care) and continue to monitor its use and presence in surface waters (Government of The Netherlands 2019), including the Vecht River. The present study suggests that the Vecht River catchment is vulnerable to pharmaceutical pollution, with 26 to 98% of its surface waters and 24 to 53% of its length under potentially unacceptable ecological risk (RQ > 1), particularly during a dry summer season. European regulation demands that national and regional authorities take action in securing water bodies' good status. To this end, the present study demonstrated the value of tailor‐made regional models and the continuous revision of ecotoxicological information. Furthermore, it highlighted the importance of assessing off‐site risks of pharmaceutical emissions using (sub‐)catchment modeling across national borders, therefore emphasizing the imperative for international cooperation. Ultimately, these results should encourage further cross‐boundary action and initiative from local authorities to comply with environmental standards via feasible and locally relevant risk‐management strategies. Otherwise, risk reduction implementations in international river networks may not be sufficiently effective.

## Supplemental Data

The Supplemental Data are available on the Wiley Online Library at https://doi.org/10.1002/etc.5062.

## Disclaimer

The authors have no conflicts of interest to declare.

## Author Contributions Statement

D. Duarte was responsible for conceptualization, methodology, formal analysis, investigation, writing–original draft, writing–review and editing, visualization; G. Niebaum and V. Lämmchen were responsible for conceptualization, methodology, validation, formal analysis, investigation, writing–original draft, writing–review and editing, visualization; E. van Heijnsbergen was responsible for methodology, validation, investigation, writing–review and editing; R. Oldenkamp was responsible for conceptualization, writing–review and editing, supervision; L. Hernandez‐Leal was responsible for resources, writing–review and editing, project administration; H. Schmitt, A. Ragas, and J. Klasmeier were responsible for conceptualization, writing–review and editing, supervision, project administration, funding acquisition.

## Supporting information

This article includes online‐only Supplemental Data.

Supporting information.

## Data Availability

Data, associated metadata, and calculation tools are available from the corresponding author (daniel.duarte@ru.nl). For data requests concerning the environmental exposure assessment, please contact J. Klasmeier (jklasmei@uni-osnabrueck.de). For data requests concerning the environmental effect assessment and aquatic ecological risk, please contact A. Ragas (a.ragas@fnwi.ru.nl).

## References

[etc5062-bib-0001] Aldekoa J , Medici C , Osorio V , Pérez S , Marcé R , Barceló D , Francés F . 2013. Modelling the emerging pollutant diclofenac with the GREAT‐ER model: Application to the Llobregat River basin. J Hazard Mater 263:207–213. 24035509 10.1016/j.jhazmat.2013.08.057

[etc5062-bib-0002] Alder AC , Schaffner C , Majewsky M , Klasmeier J , Fenner K . 2010. Fate of beta‐blocker human pharmaceuticals in surface water: Comparison of measured and simulated concentrations in the Glatt Valley watershed, Switzerland. Water Res 44:936–948. 19889439 10.1016/j.watres.2009.10.002

[etc5062-bib-0003] Aminot Y , Le , Menach K , Pardon P , Etcheber H , Budzinski H . 2016. Inputs and seasonal removal of pharmaceuticals in the estuarine Garonne River. Mar Chem 185:3–11.

[etc5062-bib-0004] Anderson PD , D'Aco VJ , Shanahan P , Chapra SC , Buzby ME , Cunningham VL , DuPlessie BM , Hayes EP , Mastrocco FJ , Parke NJ , Rader JC , Samuelian JH , Schwab BW . 2004. Screening analysis of human pharmaceutical compounds in U.S. surface waters. Environ Sci Technol 38:838–849. 14968872 10.1021/es034430b

[etc5062-bib-0005] Archundia D , Boithias L , Duwig C , Morel M‐C , Flores Aviles G , Martins JMF . 2018. Environmental fate and ecotoxicological risk of the antibiotic sulfamethoxazole across the Katari catchment (Bolivian Altiplano): Application of the GREAT‐ER model. Sci Total Environ 622–623:1046–1055. 10.1016/j.scitotenv.2017.12.026 29890574

[etc5062-bib-0006] aus der Beek T , Weber F‐A , Bergmann A , Hickmann S , Ebert I , Hein A , Küster A . 2016. Pharmaceuticals in the environment—Global occurrences and perspectives. Environ Toxicol Chem 35:823–835. 26666847 10.1002/etc.3339

[etc5062-bib-0007] Backhaus T . 2016. Environmental risk assessment of pharmaceutical mixtures: Demands, gaps, and possible bridges. AAPS J 18:804–813. 27044369 10.1208/s12248-016-9907-0

[etc5062-bib-0008] Balaam JL , Grover D , Johnson AC , Jürgens M , Readman J , Smith AJ , White S , Williams R , Zhou JL . 2010. The use of modelling to predict levels of estrogens in a river catchment: How does modelled data compare with chemical analysis and in vitro yeast assay results? Sci Total Environ 408:4826–4832. 20673965 10.1016/j.scitotenv.2010.07.019

[etc5062-bib-0009] Burns EE , Carter LJ , Snape J , Thomas‐Oates J , Boxall ABA . 2018. Application of prioritization approaches to optimize environmental monitoring and testing of pharmaceuticals. J Toxicol Environ Health B Crit Rev 21:115–141. 29714645 10.1080/10937404.2018.1465873

[etc5062-bib-0010] Caldwell DJ , D'Aco V , Davidson T , Kappler K , Murray‐Smith RJ , Owen SF , Robinson PF , Simon‐Hettich B , Straub JO , Tell J . 2019. Environmental risk assessment of metformin and its transformation product guanylurea: II. Occurrence in surface waters of Europe and the United States and derivation of predicted no‐effect concentrations. Chemosphere 216:855–865. 30385066 10.1016/j.chemosphere.2018.10.038

[etc5062-bib-0011] Capdevielle M , van Egmond R , Whelan M , Versteeg D , Hofmann‐Kamensky M , Inauen J , Cunningham V , Woltering D . 2008. Consideration of exposure and species sensitivity of triclosan in the freshwater environment. Integr Environ Assess Manag 4:15–23. 18260205 10.1897/ieam_2007-022.1

[etc5062-bib-0012] Celiz MD , Tso J , Aga DS . 2009. Pharmaceutical metabolites in the environment: Analytical challenges and ecological risks. Environ Toxicol Chem 28:2473–2484. 19663539 10.1897/09-173.1

[etc5062-bib-0013] Chaves MdJS , Barbosa SC , Malinowski MdM , Volpato D , Castro ÍB , Franco TCRDS , Primel EG . 2020. Pharmaceuticals and personal care products in a Brazilian wetland of international importance: Occurrence and environmental risk assessment. Sci Total Environ 734:139374. 32460076 10.1016/j.scitotenv.2020.139374

[etc5062-bib-0014] Chen H , Gu X , Zeng Q , Mao Z . 2019. Acute and chronic toxicity of carbamazepine on the release of chitobiase, molting, and reproduction in *Daphnia similis* . Int J Environ Res Public Health 16:209. 10.3390/ijerph16020209 PMC6351914 30642120

[etc5062-bib-0015] Coppens LJC , van Gils JAG , ter Laak TL , Raterman BW , van Wezel AP . 2015. Towards spatially smart abatement of human pharmaceuticals in surface waters: Defining impact of sewage treatment plants on susceptible functions. Water Res 81:356–365. 26102555 10.1016/j.watres.2015.05.061

[etc5062-bib-0016] Cunningham VL . 2008. Environmental exposure modeling: Application of PhATE™ and Great‐ER to human pharmaceuticals in the environment. In Kümmerer K , ed, Pharmaceuticals in the Environment. Springer, Berlin, Germany, pp 133–146.

[etc5062-bib-0017] Cuttelod A , Seddon M , Neubert E 2011. European Red List of Non‐marine Molluscs. Publications Office of the European Union, Luxembourg.

[etc5062-bib-0018] Dusi E , Rybicki M , Jungmann D. 2019. The database “Pharmaceuticlas in the Environment”—Update and new analysis. Umweltbundesamt, Dessau‐Roßlau, Germany.

[etc5062-bib-0020] Dutch National Health Care Institute . 2020. GIPdatabank: Medicines and AIDS information project. Diemen, The Netherlands.

[etc5062-bib-0021] Ebert I , Bachmann J , Kühnen U , Küster A , Kussatz C , Maletzki D , Schlüter C . 2011. Toxicity of the fluoroquinolone antibiotics enrofloxacin and ciprofloxacin to photoautotrophic aquatic organisms. Environ Toxicol Chem 30:2786–2792. 21919043 10.1002/etc.678

[etc5062-bib-0022] European Commission . 2000. Directive 2000/60/EC of the European Parliament and of the Council of 23 October 2000 establishing a framework for Community action in the field of water policy. Official Journal of the European Communities L327:1–73.

[etc5062-bib-0023] European Commission . 2006. Directive 2006/121/EC of the European Parliament and of the Council of 18 December 2006 amending Council Directive 67/548/EEC on the approximation of laws, regulations and administrative provisions relating to the classification, packaging and labelling of dangerous substances in order to adapt it to Regulation (EC) No 1907/2006 concerning the Registration, Evaluation, Authorisation and Restriction of Chemicals (REACH) and establishing a European Chemicals Agency. Official J Eur Union L396:850–856.

[etc5062-bib-0024] European Commission . 2008. Directive 2008/105/EC of the European Parliament and of the Council of 16 December 2008 on environmental quality standards in the field of water policy, amending and subsequently repealing Council Directives 82/176/EEC, 83/513/EEC, 84/156/EEC, 84/491/EEC, 86/280/EEC and amending Directive 2000/60/EC of the European Parliament and of the Council. Official J Eur Union L348:84–97.

[etc5062-bib-0025] European Commission . 2012. Directorate‐General for Health and Consumers—Opinion on the toxicity and assessment of chemical mixtures. Brussels, Belgium.

[etc5062-bib-0026] European Commission . 2018. Technical guidance for deriving environmental quality standards. Brussels, Belgium.

[etc5062-bib-0027] Feijtel T , Boeije G , Matthies M , Young A , Morris G , Gandolfi C , Hansen B , Fox K , Holt M , Koch V , Schroder R , Cassani G , Schowanek D , Rosenblom J , Holt M . 1997. Development of a geography‐referenced regional exposure assessment tool for European rivers—GREAT‐ER. Chemosphere 34:2351–2373.

[etc5062-bib-0028] Font C , Bregoli F , Acuña V , Sabater S , Marcé R . 2019. GLOBAL‐FATE (version 1.0.0): A geographical information system (GIS)‐based model for assessing contaminants fate in the global river network. Geosci Model Dev 12:5213–5228.

[etc5062-bib-0029] Gómez‐Canela C , Pueyo V , Barata C , Lacorte S , Marcé RM . 2019. Development of predicted environmental concentrations to prioritize the occurrence of pharmaceuticals in rivers from Catalonia. Sci Total Environ 666:57–67. 30784823 10.1016/j.scitotenv.2019.02.078

[etc5062-bib-0101] Gomez Cortes L , Marinov D , Sanseverino I , Navarro Cuenca A , Niegowska M , Porcel Rodriguez E , Lettieri T . 2020. Selection of substances for the 3rd Watch List under the Water Framework Directive. EUR 30297 EN. Publications Office of the European Union, Luxembourg.

[etc5062-bib-0030] Government of The Netherlands . 2019. Reducing pharmaceutical residues in water: A chain approach. Amsterdam, The Netherlands.

[etc5062-bib-0031] Grill G , Khan U , Lehner B , Nicell J , Ariwi J . 2016. Risk assessment of down‐the‐drain chemicals at large spatial scales: Model development and application to contaminants originating from urban areas in the Saint Lawrence River basin. Sci Total Environ 541:825–838. 26437353 10.1016/j.scitotenv.2015.09.100

[etc5062-bib-0032] Han EJ , Lee DS . 2017. Significance of metabolites in the environmental risk assessment of pharmaceuticals consumed by human. Sci Total Environ 592:600–607. 28318699 10.1016/j.scitotenv.2017.03.044

[etc5062-bib-0033] Hanamoto S , Nakada N , Yamashita N , Tanaka H . 2013. Modeling the photochemical attenuation of down‐the‐drain chemicals during river transport by stochastic methods and field measurements of pharmaceuticals and personal care products. Environ Sci Technol 47:13571–13577. 24199688 10.1021/es4035478

[etc5062-bib-0034] Hannah R , D'Aco VJ , Anderson PD , Buzby ME , Caldwell DJ , Cunningham VL , Ericson JF , Johnson AC , Parke NJ , Samuelian JH , Sumpter JP . 2009. Exposure assessment of 17alpha‐ethinylestradiol in surface waters of the United States and Europe. Environ Toxicol Chem 28:2725–2732. 19645524 10.1897/08-622.1

[etc5062-bib-0035] Heberer T , Feldmann D . 2005. Contribution of effluents from hospitals and private households to the total loads of diclofenac and carbamazepine in municipal sewage effluents—Modeling versus measurements. J Hazard Mater 122:211–218. 15967276 10.1016/j.jhazmat.2005.03.007

[etc5062-bib-0036] Henning‐de Jong I , Ragas AMJ , Hendriks HWM , Huijbregts MAJ , Posthuma L , Wintersen A , Jan Hendriks A . 2009. The impact of an additional ecotoxicity test on ecological quality standards. Ecotoxicol Environ Saf 72:2037–2045. 19748120 10.1016/j.ecoenv.2009.08.009

[etc5062-bib-0037] Hernandez AF , Buha A , Constantin C , Wallace DR , Sarigiannis D , Neagu M , Antonijevic B , Hayes AW , Wilks MF , Tsatsakis A . 2019. Critical assessment and integration of separate lines of evidence for risk assessment of chemical mixtures. Arch Toxicol 93:2741–2757. 31520250 10.1007/s00204-019-02547-x

[etc5062-bib-0038] Hernando‐Amado S , Coque TM , Baquero F , Martínez JL . 2019. Defining and combating antibiotic resistance from One Health and global health perspectives. Nat Microbiol 4:1432–1442. 31439928 10.1038/s41564-019-0503-9

[etc5062-bib-0039] Hüffmeyer N , Klasmeier J , Matthies M . 2009. Geo‐referenced modeling of zinc concentrations in the Ruhr River basin (Germany) using the model GREAT‐ER. Sci Total Environ 407:2296–2305. 19150732 10.1016/j.scitotenv.2008.11.055

[etc5062-bib-0040] Innovative Medicines Initiative . 2019. iPiE Summary Database Search (iPiE‐Sum). Brussels, Belgium.

[etc5062-bib-0042] Jarvis AL , Bernot MJ , Bernot RJ . 2014. Relationships between the psychiatric drug carbamazepine and freshwater macroinvertebrate community structure. Sci Total Environ 496:499–509. 25108252 10.1016/j.scitotenv.2014.07.086

[etc5062-bib-0043] Jobling S , Williams R , Johnson A , Taylor A , Gross‐Sorokin M , Nolan M , Tyler CR , van Aerle R , Santos E , Brighty G . 2006. Predicted exposures to steroid estrogens in U.K. rivers correlate with widespread sexual disruption in wild fish populations. Environ Health Perspect 114(Suppl. 1):32–39. 16818244 10.1289/ehp.8050 PMC1874167

[etc5062-bib-0044] Johnson AC , Chen Y . 2017. Does exposure to domestic wastewater effluent (including steroid estrogens) harm fish populations in the UK? Sci Total Environ 589:89–96. 28273597 10.1016/j.scitotenv.2017.02.142

[etc5062-bib-0045] Johnson I , Harvey P 2002. Study on the scientific evaluation of 12 substances in the context of endocrine disruptor priority list of actions. WRc‐NSF UC 6052. WRc‐NSF, Oakdale, UK.

[etc5062-bib-0047] Kapo KE , DeLeo PC , Vamshi R , Holmes CM , Ferrer D , Dyer SD , Wang X , White‐Hull C . 2016. iSTREEM®: An approach for broad‐scale in‐stream exposure assessment of “down‐the‐drain” chemicals. Integr Environ Assess Manag 12:782–792. 27156081 10.1002/ieam.1793

[etc5062-bib-0048] Kehrein N , Berlekamp J , Klasmeier J . 2015. Modeling the fate of down‐the‐drain chemicals in whole watersheds: New version of the GREAT‐ER software. Environ Model Softw 64:1–8.

[etc5062-bib-0049] Kienzler A , Connors KA , Bonnell M , Barron MG , Beasley A , Inglis CG , Norberg‐King TJ , Martin T , Sanderson H , Vallotton N , Wilson P , Embry MR . 2019. Mode of action classifications in the EnviroTox database: Development and implementation of a consensus MOA classification. Environ Toxicol Chem 38:2294–2304. 31269286 10.1002/etc.4531 PMC6851772

[etc5062-bib-0050] Klein EY , van Boeckel TP , Martinez EM , Pant S , Gandra S , Levin SA , Goossens H , Laxminarayan R . 2018. Global increase and geographic convergence in antibiotic consumption between 2000 and 2015. Proc Natl Acad Sci USA 115:E3463–E3470. 29581252 10.1073/pnas.1717295115 PMC5899442

[etc5062-bib-0051] Kunkel U , Radke M . 2012. Fate of pharmaceuticals in rivers: Deriving a benchmark dataset at favorable attenuation conditions. Water Res 46:5551–5565. 22898670 10.1016/j.watres.2012.07.033

[etc5062-bib-0052] Lämmchen V , Niebaum G , Berlekamp J , Klasmeier J . 2021. Geo‐referenced simulation of pharmaceuticals in whole watersheds: Application of GREAT‐ER 4.1 in Germany. Environ Sci Pollut Res in press. 10.1007/s11356-020-12189-7 PMC8106600 33411301

[etc5062-bib-0053] Lange A , Paull GC , Coe TS , Katsu Y , Urushitani H , Iguchi T , Tyler CR . 2009. Sexual reprogramming and estrogenic sensitization in wild fish exposed to ethinylestradiol. Environ Sci Technol 43:1219–1225. 19320183 10.1021/es802661p

[etc5062-bib-0054] Li J , Cui M . 2020. Kinetic study on the sorption and degradation of antibiotics in the estuarine water: An evaluation based on single and multiple reactions. Environ Sci Pollut Res Int 27:42104–42114. 32705565 10.1007/s11356-020-10194-4

[etc5062-bib-0055] Lindim C , van Gils J , Cousins IT . 2016. A large‐scale model for simulating the fate & transport of organic contaminants in river basins. Chemosphere 144:803–810. 26414740 10.1016/j.chemosphere.2015.09.051

[etc5062-bib-0056] Liu X , Lv K , Deng C , Yu Z , Shi J , Johnson AC . 2019. Persistence and migration of tetracycline, sulfonamide, fluoroquinolone, and macrolide antibiotics in streams using a simulated hydrodynamic system. Environ Pollut 252:1532–1538. 31277022 10.1016/j.envpol.2019.06.095

[etc5062-bib-0057] Lulofs KRD , Coenen FHJM . 2007. Cross border co‐operation on water quality in the Vecht River basin. In Verwijmeren J , Wiering MA , eds, Many Rivers to Cross: Cross Border Co‐operation in River Management. Eburon Uitgeverij, Delf, The Netherlands, pp 71–93.

[etc5062-bib-0058] Meyer W , Reich M , Beier S , Behrendt J , Gulyas H , Otterpohl R . 2016. Measured and predicted environmental concentrations of carbamazepine, diclofenac, and metoprolol in small and medium rivers in northern Germany. Environ Monit Assess 188:487. 27465046 10.1007/s10661-016-5481-2

[etc5062-bib-0059] Moermond CTA , Kase R , Korkaric M , Ågerstrand M . 2016a. CRED: Criteria for reporting and evaluating ecotoxicity data. Environ Toxicol Chem 35:1297–1309. 26399705 10.1002/etc.3259

[etc5062-bib-0060] Moermond CTA , Montforts MHMM , Roex EWM , Venhuis BJ 2020. Medicijnresten en waterkwaliteit: Een update. 2020‐0088. National Institute of Public Health and Environment (RIVM), Bilthoven, The Netherlands.

[etc5062-bib-0061] Moermond CTA , Smit CE , van Leerdam RC , van der Aa NGFM , Montforts MHMM . 2016b. Geneesmiddelen en waterkwaliteit, National Institute of Public Health and Environment (RIVM), Bilthoven, The Netherlands.

[etc5062-bib-0062] Molander L , Ågerstrand M , Rudén C . 2009. WikiPharma—A freely available, easily accessible, interactive and comprehensive database for environmental effect data for pharmaceuticals. Regul Toxicol Pharmacol 55:367–371. 19720105 10.1016/j.yrtph.2009.08.009

[etc5062-bib-0063] Morley SK , Brito TV , Welling DT . 2018. Measures of model performance based on the log accuracy ratio. Space Weather 16:69–88.

[etc5062-bib-0064] Musolff A , Leschik S , Möder M , Strauch G , Reinstorf F , Schirmer M . 2009. Temporal and spatial patterns of micropollutants in urban receiving waters. Environ Pollut 157:3069–3077. 19525045 10.1016/j.envpol.2009.05.037

[etc5062-bib-0065] Oelkers K . 2020. The accessibility of data on environmental risk assessment of pharmaceuticals—Are environmental risk assessments information on emissions with respect to international and European environmental information law? Regul Toxicol Pharmacol 111:104571. 31893528 10.1016/j.yrtph.2019.104571

[etc5062-bib-0066] Oldenkamp R , Hoeks S , Čengić M , Barbarossa V , Burns EE , Boxall ABA , Ragas AMJ . 2018. A high‐resolution spatial model to predict exposure to pharmaceuticals in European surface waters: ePiE. Environ Sci Technol 52:12494–12503. 30303372 10.1021/acs.est.8b03862 PMC6328286

[etc5062-bib-0067] Oosterhuis M , Sacher F , ter Laak TL . 2013. Prediction of concentration levels of metformin and other high consumption pharmaceuticals in wastewater and regional surface water based on sales data. Sci Total Environ 442:380–388. 23183121 10.1016/j.scitotenv.2012.10.046

[etc5062-bib-0068] Palma P , Fialho S , Lima A , Novais MH , Costa MJ , Montemurro N , Pérez S , de Alda ML . 2020. Pharmaceuticals in a Mediterranean basin: The influence of temporal and hydrological patterns in environmental risk assessment. Sci Total Environ 709:136205. 31905561 10.1016/j.scitotenv.2019.136205

[etc5062-bib-0069] Patel M , Kumar R , Kishor K , Mlsna T , Pittman CU Jr , Mohan D . 2019. Pharmaceuticals of emerging concern in aquatic systems: Chemistry, occurrence, effects, and removal methods. Chem Rev 119:3510–3673 30830758 10.1021/acs.chemrev.8b00299

[etc5062-bib-0070] Popelka SJ , Smith LC . 2020. Rivers as political borders: A new subnational geospatial dataset. Water Policy 22:293–312.

[etc5062-bib-0071] Posthuma L , Altenburger R , Backhaus T , Kortenkamp A , Müller C , Focks A , de Zwart D , Brack W . 2019. Improved component‐based methods for mixture risk assessment are key to characterize complex chemical pollution in surface waters. Environ Sci Eur 31:1204.

[etc5062-bib-0072] Posthuma L , Brown CD , de Zwart D , Diamond J , Dyer SD , Holmes CM , Marshall S , Burton GA Jr. 2018. Prospective mixture risk assessment and management prioritizations for river catchments with diverse land uses. Environ Toxicol Chem 37:715–728. 28845901 10.1002/etc.3960 PMC5873277

[etc5062-bib-0073] Saaristo M , Brodin T , Balshine S , Bertram MG , Brooks BW , Ehlman SM , McCallum ES , Sih A , Sundin J , Wong BBM , Arnold KE . 2018. Direct and indirect effects of chemical contaminants on the behaviour, ecology and evolution of wildlife. Proc Biol Sci 285:20181297. 30135169 10.1098/rspb.2018.1297 PMC6125903

[etc5062-bib-0074] Scheurer M , Sacher F , Brauch HJ . 2009. Occurrence of the antidiabetic drug metformin in sewage and surface waters in Germany. J Environ Monit 11:1608–1613. 19724829 10.1039/b909311g

[etc5062-bib-0075] Schowanek D , Webb S . 2002. Exposure simulation for pharmaceuticals in European surface waters with GREAT‐ER. Toxicol Lett 131:39–50. 11988357 10.1016/s0378-4274(02)00064-4

[etc5062-bib-0076] Shultz S , Baral HS , Charman S , Cunningham AA , Das D , Ghalsasi GR , Goudar MS , Green RE , Jones A , Nighot P , Pain DJ , Prakash V . 2004. Diclofenac poisoning is widespread in declining vulture populations across the Indian subcontinent. Proc Biol Sci 271(Suppl. 6):S458–S460. 15801603 10.1098/rsbl.2004.0223 PMC1810094

[etc5062-bib-0077] Teubner D , Klein R , Paulus M , Wesch C . 2019. Changes of fish growth in German rivers. Curr Opin Environ Sci Health 11:59–64.

[etc5062-bib-0078] Thomas MA , Joshi PP , Klaper RD . 2012. Gene‐class analysis of expression patterns induced by psychoactive pharmaceutical exposure in fathead minnow (*Pimephales promelas*) indicates induction of neuronal systems. Comp Biochem Physiol Toxicol Pharmacol 155:109–120. 10.1016/j.cbpc.2011.05.014 PMC3219835 21684349

[etc5062-bib-0079] Tiedeken EJ , Tahar A , McHugh B , Rowan NJ . 2017. Monitoring, sources, receptors, and control measures for three European Union watch list substances of emerging concern in receiving waters—A 20 year systematic review. Sci Total Environ 574:1140–1163. 27741430 10.1016/j.scitotenv.2016.09.084

[etc5062-bib-0080] Trade Association for the Research‐Based Pharmaceutical Industry in Sweden . 2019. FASS database. Stockholm, Sweden. [Cited March 2019]. Available from: https://www.fass.se/

[etc5062-bib-0081] US Environmental Protection Agency . 2019. ECOTOXicology Knowledgebase System User Guide, Ver 5.3. EPA/600/R‐20/087. Washington DC. [cited 2019 May 2]. Available from: https://cfpub.epa.gov/ecotox/

[etc5062-bib-0082] van Nuijs ALN , Covaci A , Beyers H , Bervoets L , Blust R , Verpooten G , Neels H , Jorens PG . 2015. Do concentrations of pharmaceuticals in sewage reflect prescription figures? Environ Sci Pollut Res Int 22:9110–9118. 25874419 10.1007/s11356-014-4066-2

[etc5062-bib-0083] Verlicchi P , Al Aukidy M , Zambello E . 2012. Occurrence of pharmaceutical compounds in urban wastewater: Removal, mass load and environmental risk after a secondary treatment—A review. Sci Total Environ 429:123–155. 22583809 10.1016/j.scitotenv.2012.04.028

[etc5062-bib-0084] Vieno N , Sillanpää M . 2014. Fate of diclofenac in municipal wastewater treatment plant—A review. Environ Int 69:28–39. 24791707 10.1016/j.envint.2014.03.021

[etc5062-bib-0085] Vissers M , Vergouwen L , Witteveen S 2017. Landelijke hotspotanalyse geneesmiddelen RWZI's. STOWA, Amersfoort, The Netherlands.

[etc5062-bib-0086] Wöhler L , Niebaum G , Krol M , Hoekstra AY . 2020. The grey water footprint of human and veterinary pharmaceuticals. Water Res X 7:100044. 32462135 10.1016/j.wroa.2020.100044 PMC7242788

[etc5062-bib-0087] Young HK . 1993. Antimicrobial resistance spread in aquatic environments. J Antimicrob Chemother 31:627–635. 8335494 10.1093/jac/31.5.627

[etc5062-bib-0088] Zha J , Sun L , Zhou Y , Spear PA , Ma M , Wang Z . 2008. Assessment of 17alpha‐ethinylestradiol effects and underlying mechanisms in a continuous, multigeneration exposure of the Chinese rare minnow (*Gobiocypris rarus*). Toxicol Appl Pharmacol 226:298–308. 18054974 10.1016/j.taap.2007.10.006

[etc5062-bib-0089] Zhang L , Cao Y , Hao X , Zhang Y , Liu J . 2015. Application of the GREAT‐ER model for environmental risk assessment of nonylphenol and nonylphenol ethoxylates in China. Environ Sci Pollut Res Int 22:18531–18540. 26358209 10.1007/s11356-015-5352-3

[etc5062-bib-0090] Zhou S , Di Paolo C , Wu X , Shao Y , Seiler T‐B , Hollert H . 2019. Optimization of screening‐level risk assessment and priority selection of emerging pollutants—The case of pharmaceuticals in European surface waters. Environ Int 128:1–10. 31029973 10.1016/j.envint.2019.04.034

