# Supplementary material for: Ecological Risk Assessment of Pharmaceuticals in the Transboundary Vecht River (Germany and The Netherlands)
Source: Environ Toxicol Chem. 2021 May 28;41(3):648–62. doi: 10.1002/etc.5062 (PMC9290585; doi:10.1002/etc.5062)
Supplement: Supplementary file 1 — Supporting information. [file ETC-41-648-s001.docx]

**TITLE**

Ecological risk assessment of pharmaceuticals in the transboundary Vecht River (Germany/Netherlands)

**S1. SUPPLEMENTARY**

**TABLE S1**: Pharmaceutical consumption rates.

| *Compound* | *German per capita consumption Vecht catchment [kg/(cap yr)] ^a^* | *Dutch per capita consumption Vecht catchment [kg/(cap yr)] ^b^* | *Ratio German to Dutch per bed consumption ^c^* |
| --- | --- | --- | --- |
| 17α-Ethinylestradiol | 1.50x10^-7^ | 6.39x10^-7^ | n.a.^d^ |
| Carbamazepine | 4.36x10^-4^ | 4.56x10^-4^ | 203% |
| Ciprofloxacin | 2.75 x10^-4^ | 2.32x10^-4^ | 131% |
| Cyclophosphamide ^e^ | 1.93 x10^-6^ | 0 | 46% |
| Diclofenac | 6.73x10^-4^ | 2.54x10^-4^ | 198% |
| Erythromycin | 3.14x10^-4^ | 1.98x10^-5^ | 117% |
| Metformin | 1.36x10^-2^ | 1.97x10^-2^ | 144% |
| Metoprolol | 1.47x10^-3^ | 1.74x10^-3^ | 123% |
| ^a^ IQVIA Commercial GmbH & Co. OHG, calculations based on IMS PharmaScope^®^ (2018).  ^b^ Dutch Foundation for Pharmaceutical Statistics (2018).  ^c^ Annual per bed consumption rates were calculated as the mass of prescribed pharmaceuticals in a hospital devided by the number of beds in the respective hospital. These values were averaged for German and Dutch hospitals, respectively. Due to the limited number of hospitals which provided data and data security issues only ratios of the average per bed consumption ratios can be displayed. Excluding ethinylestradiol and cyclophosphamide, the per-bed consumption rate is 1 to 40 times higher than the per capita consumption rates of the respective countries.  ^d^ In both countries, no hospital consumption data was reported; n.a., not applicable.  ^e^ Cyclophosphamide is restricted to clinical use in the Netherlands. SFK only collects domestic pharmaceutical consumption. Therefore, no domestic cyclophosphamide use is recorded in for the Netherlands. | | | |

**TABLE S2:** Pharmaceutical excretion data. Urinary and faecal excretion percentages. Glucuronide conjugates of the parent compound are shown in brackets ^a^. For the modelling exercise (sixth column), mean urinary excretion and 20% of mean faecal excretion were applied.

| *Compound* | *Urine*  *(+ conjugates) [%]* | *Faeces*  *(+ conjugates) [%]* | *Urine + faeces*  *(+ conjugates) [%]* | *Source* | *Modelled fraction entering STPs [%]* |
| --- | --- | --- | --- | --- | --- |
| 17α-Ethinylestradiol | 10.1 (17.2) | 23.1 |  | 12 | 32% |
| Carbamazepine | < 10 | < 30 |  | 1 | 15% |
|  | 0.8 (11) | 13 (?) |  | 2 |  |
|  | 2 | < 28 |  | 3 |  |
|  | 1.44 | 12.3 |  | 4 |  |
|  |  |  | 2.7 – 15% (0%) | 5 |  |
|  | 1 | 28 |  | 6 |  |
|  |  |  | 1 – 2% (~ 30%) | 7 |  |
|  |  |  | 31% | 8 |  |
| Ciprofloxacin | 40 – 50 | < 20 – 35 |  | 1 | 54% |
|  | < 70 | 15 |  | 1 |  |
|  | 44.7 | 25 |  | 3 |  |
|  | 61.5 | 15.2 |  | 3 |  |
| Cyclophosphamide | 25 | “small amounts” |  | 1 | 25% |
| Diclofenac | ? (< 15) | < 5 |  | 1 | 10% |
|  | 2 – 23 ^b^ | 1 – 4 ^b^ |  | 9 |  |
|  |  |  | 1 (10 – 15) | 5 |  |
|  |  |  | 0.05 – 0.1 (0.5 – 1.5) | 5 |  |
|  | 6 | < 35 |  | 6 |  |
|  |  |  | 2% (15) | 8 |  |
|  |  |  | 15 (< 1) | 7 |  |
|  |  |  | < 1 (5 – 10) | 10 |  |
| Erythromycin | 4 – 20 | 40 – 50 |  | 11 | 19% |
|  |  |  | 4 | 8 |  |
|  | 5 – 10 | “large amounts” |  | 1 |  |
|  | 5 | “mainly” |  | 3 |  |
|  | 12 – 15 | “mainly” |  | 3 |  |
| Metformin | 30 – 50 | 30 |  | 1 | 74% |
|  | 35 – 50 | 30 |  | 13 |  |
|  | 79 | 0 |  | 13 |  |
|  | 100 | 0 |  | 14 |  |
|  | 100 | 0 |  | 15 |  |
| Metoprolol | < 10 | - |  | 1 | 8% |
|  | < 5 | - |  | 3 |  |
|  | 3 – 10 |  |  | 16 |  |
|  | 9.4 | - |  | 17 |  |
|  | < 5.2 | - |  | 17 |  |
|  |  |  | 7 | 8 |  |
|  |  |  | 3 – 10 | 7 |  |
|  | 5 – 10 | - |  | 10 |  |
| 1. Moffat et al. (2011). 2. Bahlmann et al. (2014). 3. Swissmedic (2020). 4. Björlenius et al. (2018). 5. Heberer and Feldmann (2005). 6. Zhang et al. (2008). 7. Ternes and Joss (2008). 8. Khan and Ongerth (2004). 9. Johnson et al. (2007). 10. Kummerer et al. (2011). 11. Göbel et al. (2005). 12. Johnson and Williams (2004). 13. Tucker et al. (1981). 14. Robert et al. (2003). 15. Bristol-Myers Squibb Products & Medicines (2018). 16. Alder et al. (2010). 17. Regårdh et al. (1974).  ^a^ Glucuronide conjugates can react back to the parent compound in the sewer (Gao et al. 2017; Heberer and Feldmann 2005; Kumar et al. 2012). For this study, we assume that the entire fraction excreted as glucuronide associated parent compound will react back to the parent compound in the sewer. Therefore, we aggregate the excretion rates of the parent compound and the glucuronide conjugates of the parent compound in a single excretion rate.  ^b^ No distinction between parent compound and conjugates. | | | | | |

Except for diclofenac and erythromycin, all compounds were applied systematically solely. For the latter STP inflow loads ($L_{in}$ [kg/yr]) were calculated as

$$L_{in}=pCC\times Inh\times f_{ex}$$

where $pCC$ is the per capita consumption rate [kg/(cap yr)], $Inh$ [cap] is the number of inhabitants in the STP catchment and $f_{ex}$ is the fraction that is excreted in the unchanged or conjugated state.

Only the absorbed portion of topically applied erythromycin and diclofenac are thought to undergo metabolism. Sioufi et al. (1994) found relative proportions of parent compounds and metabolites after topical application compared to oral application for diclofenac. The portion that is not absorbed either goes into clothing, bandages or is wiped off with e.g. paper and then thrown in the trash (Heberer and Feldmann 2005). According to Heberer and Feldmann (2005), STP inflow loads of diclofenac can be estimated as

$$L_{in}=(f_{sys}\times f_{ex}+f_{top}\times f_{ab}\times f_{ex}+f_{top}\left( 1-f_{ab} \right))\times pCC\times Inh$$

where $f_{sys}$ is the systematically applied fraction, $f_{top}$ is the topically applied fraction and $f_{ab}$ the fraction that is absorbed after topical application. For the scope of this study we use worst case estimations and assume that the fraction which is not absorbed (1 – $f_{ab}$_)_ ends up in the wastewater, e.g. via washing of clothing or bandages. In the model of Heberer and Feldmann (2005) it is assumed that 100% of the parenterally or orally administered dose is absorbed leading to the same excretion rate regardless of the route of administration. This model is also used to calculate the influent loads of erythromycin. Parameters for Germany and the Netherlands are shown in Table S3. The result for diclofenac is that in Germany 52 % and in the Netherlands 15 % of the total prescribed mass ends up in wastewater. For erythromycin this results in 21 % and 31 % for Germany and the Netherlands, respectively.

**TABLE S3:** Diclofenac and erythromycin inflow model parameters.

|  | *Germany ^1^* | |  | *Netherlands ^2^* | |  |  |  |
| --- | --- | --- | --- | --- | --- | --- | --- | --- |
| *Compound* | $f_{sys}$ | $f_{top}$ |  | $f_{sys}$ | $f_{top}$ |  | $f_{ab}$ | $f_{ex}$*^5^* |
| Diclofenac | 0.51 | 0.49 |  | 0.97 | 0.03 |  | 0.07 ^3^ | 0.10 |
| Erythromycin | 0.99 | 0.01 |  | 0.86 | 0.14 |  | 0.00 ^4^ | 0.19 |
| ^1^ IQVIA Commercial GmbH & Co. OHG, calculations based on IMS PharmaScope® (2018). ^2^ Dutch Foundation for Pharmaceutical Statistics (2018). ^3^ Hui et al. (1998). ^4^ Systematically exposure of topically applied erythromycin is negligible (Carls et al. 2014). ^5^ Table S2. | | | | | | | | |

**TABLE S4:** Summary of removal efficiencies published in literature. Removal efficiencies have to be interpreted as percentage change of mass loading in effluent versus influent. Negative removal efficiencies may occur when the back-reaction of labile intermediates to the parent compound outweigh the actual removal or due to experimental and analytical uncertainty for compounds with low removal efficiencies (< 10 %). STP, sewage treatment plant; SD, standard deviation.

| *Compound* | *Number of STPs* | *Mean [%]* | *SD [%]* | *Median [%]* | *Sources* |
| --- | --- | --- | --- | --- | --- |
| 17α-Ethinylestradiol | 3 | 72.5 | 5.5 | 70.5 | 3, 18 |
| Carbamazepine | 33 | -5.8 | 27.5 | 0.0 | 1, 3, 4, 7, 8, 10, 11, 12, 13, 15, 18, 20, 21 |
| Ciprofloxacin | 22 | 71.1 | 20.1 | 78.0 | 1, 4, 6, 9, 21 |
| Cyclophosphamide | 1 | 59.0 | 0.0 | 59.0 | 2 |
| Diclofenac | 19 | 25.5 | 22.7 | 31.2 | 3, 4, 8, 11, 12, 13, 15, 17, 18, 19, 20 |
| Erythromycin | 21 | 14.0 | 29.7 | 14.6 | 1, 4, 5, 6, 8, 9, 10, 12, 13, 14, 16, 18 |
| Metformin | 6 | 97.4 | 1.2 | 97.5 | 4, 11, 15 |
| Metoprolol | 16 | 22.1 | 27.6 | 22.9 | 4, 7, 8, 11, 12, 13, 15, 18, 19, 21, 22 |
| 1. Castiglioni et al. (2006). 2. Česen et al. (2015). 3. Clara et al. (2005). 4. Jesus Gaffney et al. (2017). 5. Göbel et al. (2007). 6. Guerra et al. (2014). 7. Gurke et al. (2015). 8 Kasprzyk-Hordern et al. (2009). 9. Li and Zhang (2011). 10. Nakada et al. (2007). 11. Oosterhuis et al. (2013). 12. Radjenovic et al. (2007). 13 Radjenović et al. (2009). 14. Roberts and Thomas (2006). 15. Sacher (2014). 16. Senta et al. (2019). 17. Sui et al. (2011). 18. Ternes et al. (2007). 19. Thomas et al. (2007). 20. Vergeynst et al. (2015). 21. Vieno et al. (2006). 22. Wick et al. (2009). | | | | | |

**TABLE S5:** Parametrization of in-stream processes. CBZ, carbamazepine; CIP, ciprofloxacin; CYL, cyclophosphamide; DFC, diclofenac; ERY, erythromycin; EE2, 17α-ethinylestradiol; MET, metformin; MEP, metoprolol.

| *Compound* | *Surface photolysis rates ^a^ [1/h]* | *Source* | *First order degradation rate ^a^ [1/h]* | *Source* | *Bio degradation rate [1/h]* | *Source* | *K_d_ ^b^ [L/kg]* | *Source* |
| --- | --- | --- | --- | --- | --- | --- | --- | --- |
| *CBZ* | *ScnAC:*  *1.1x10^-4^*  *ScnDS:*  *2.2x10^-4^* | *Estimated with a quantum yield of 1.1x10^-5^ (Calisto et al. 2011)* |  |  | *< 1x10^-4^* | *Durán-Álvarez et al. (2015)* | *13.3 ^d^* | *Radović et al. (2016)* |
| *CIP* | *ScnAC:*  *0.647*  *ScnDS:*  *1.311* | *Estimated with an average quantum yield of 8.5x10^-3^ (at pH 7.5)* |  |  | *No degradation* | *Girardi et al. (2011)* | *250* | *Tolls (2001)* |
| *CYC* |  |  | *7x10^-4^* | *Buerge et al. (2006)* | *< 1x10^-4^* | *Lutterbeck et al. (2016)* | *4.4 ^e^* | *Azuma et al. (2017)* |
| *DFC* | *ScnAC:*  *0.018*  *ScnDS:*  *0.049* | *Estimated with a quantum yield of 0.038 (Andreozzi et al. 2003)* |  |  | *Recalcitrant* | *Lahti and Oikari (2011)* | *14.4 ^d^* | *Radović et al. (2016)* |
| *ERY* |  |  | *0.003* | *Batchu et al. (2014)* | *< 1x10^-4^* | *Alexy et al. (2004)* | *139.7 ^d^* | *Radović et al. (2016)* |
| *EE2* | *0.0029 ^c^* | *Jürgens et al. (2002)* |  |  | *Resistant to biodegradation* | *Zuo et al. (2013)* | *278* | *Ternes et al. (2004)* |
| *MET* |  |  | *0.0012* | *Neamţu et al. (2014)* | *Not readily biodegradable* | *Trautwein and Kümmerer (2011)* | *19* | *Scheurer et al. (2012)* |
| *MEP* |  |  | *0* | *Baena-Nogueras et al. (2017)* | *0.001* | *Baena-Nogueras et al. (2017)* | *18.1^d^* | *Radović et al. (2016)* |
| *^a^ Seasonal surface photolysis rates were estimated based on wavelength-dependent sunlight intensities at 50 degree north latitude (Apell and McNeill 2019) and available light absorption spectra of the substance. Quantum yields were taken from the literature. No quantum yields and no seasonal photolysis rates were available for ethinylestradiol. Therefore, we applied the same literature photolysis rate for both scenarios. Cyclophosphamide, erythromycin, metformin and metoprolol do not effectively absorb sunlight in the photochemically relevant wavelength range between 295 nm - 400 nm. For these compounds, lumped pseudo first order degradation rates reported in the literature were used without correction for seasonal influences due to a lack of more detailed information.*  *^b^ The distribution coefficient K_d_ is an input parameter of the GREAT-ER model to estimate the chemical fraction of a chemical prone to sedimentation in a river segment. Sedimentation is modelled using the equilibrium distribution assumption represented by an average K_d_ value between suspended matter and water. The model includes a basic assumption on the average suspended matter concentration which is used to estimate the adsorbed fraction. Spatial information about the composition and properties of suspended matter in the Vecht River catchment was not available. Furthermore, information about K_d_ values between suspended matter and water in natural rivers was not available for the investigated APIs. However, K_d_ values are reported in the literature for the sediment-water, soil-water and sludge-water equilibrium. Therefore, those values were used as a proxy to describe the suspended matter water distribution in the Vecht River catchment.*  *^c^ Based on assumed 12 hours of sunlight exposure per day.*  *^d^ Average value of four sediments.*  *^e^ Average value of two sediments.* | | | | | | | | |

*S1.1. Monitoring campaign*

As a part of a one-year sampling campaign of bacteria and bacteria resistance genes in the Vecht catchment (omitted author, in preparation) a subset of collected STP and in-stream samples was analysed for pharmaceuticals (Tables S6-8). The selection of STPs was the same as in the grand sampling campaign. Selection was based on the plant location (Germany/Netherlands), the plant scale (small to large) and, if the plant was or was not treating hospital wastewater. Approximately 50 % of STP influent and effluent samples were analysed for pharmaceuticals. This was thought to be sufficient to cover pharmaceutical variability in STP influent and effluent in Germany and the Netherlands. The sampling months of the STP measurements are displayed in Table S7. For two STPs a gradient measurement was performed, i.e. Hardenberg and Steinfurt-Burgsteinfurt. For these plants, one surface water sample upstream of each plant was taken (sampling sites H00 and B00 respectively), as well as several surface water samples downstream of the plants (sampling sites H01-H06 and B02-B06 respectively). Furthermore, several surface water locations were sampled for other interests. One sample was taken on the location where the river crosses the German-Dutch border (sampling site G11). The other sampling sites were distributed across the catchment (sampling sites G02, G04, G05, G07, G08, G09, G10). The in-stream sampling sites represent a subset of the sampling sites in the grand monitoring campaign and were taken on locations that were important for evaluation of the GREAT-ER model. At each of these sampling sites a fraction of samples was analysed for pharmaceuticals. These fractions were selected based on the date of sampling and the hydrological conditions on the respective day. At Dutch sampling sites pumping activities were also taken into account. The in-stream sampling sites and their allocation to the scenarios are summarized in Table S8.

**TABLE S6:** Number of the sampling sites and number of samples taken in the Vecht catchment. For a comprehensive overview see omitted author (unpublished manuscript). STP, sewage treatment plant.

|  | *STP (influent and effluent)* | *In-stream* |
| --- | --- | --- |
| Germany | Gronau, Nordhorn, Schüttorf, Steinfurt-Burgsteinfurt (n_influent_ = 25, n_effluent_ = 25) | B00, B02, B03, B04, B05, B06. G02, G04, G05 (n_ScnDS_ = 18, n_ScnAC_ = 28) |
| Netherlands | Almelo-Sumpel, Dalfsen, Enschede-West, Hardenberg, Ootmarsum, Vroomshoop (n_influent_ = 34, n_effluent_ = 33) | H00, H02, H03, H04, H06, G06, G07, G09, G10, G11(n_ScnDS_ = 19, n_ScnAC_ = 27) |

**TABLE S7:** Sampling dates of the subset of samples and allocation to scenarios.

| Sampling month and year | STP ^a^ |
| --- | --- |
| July 2018 | W01, W02, W04, W05, W07, W09, W10, W11 |
| August 2018 | W01, W02, W04, W05, W07, W09, W10, W11 |
| November 2018 | W01, W02, W04, W05, W07, W09, W10, W11 |
| December 2018 | W03, W06 |
| January 2019 | W03, W04, W06, W07 |
| February 2019 | W01, W02, W03, W04, W05, W07, W09, W10 |
| March 2019 | W03, W04, W05, W06, W07, W09 |
| April 2019 | W01, W02, W03, W04, W05, W06, W07, W10, W11 |
| May 2019 | W02, W03, W06, W09, W11 |
| ^a^ W01, Hardenberg. W02, Enschede. W03, Steinfurt-Burgsteinfurt. W04, Nordhorn. W05, Ootmarsum. W06, Gronau. W07, Schuettorf. W09, Almelo-Sumpel. W10, Dalfsen. W11, Vroomshoop. | |

**TABLE S8:** Sampling dates of the subset of samples and allocation to scenarios. ScnDS, Dry summer scenario; ScnAC, average condition scenario.

| Allocated scenario | Sampling month and year | Sampling sites |
| --- | --- | --- |
| ScnDS | June 2018 | B00, B02, B03, B04, B05, B06, G02, G04, G05, G07, G08, G09, G10, G11, H00, H02, H03, H04, H06 |
| ScnDS | August 2018 | B00, B03, B04, B05, B06, G02, G04, G05, G07, G08, G09, G10, G11, H00, H03, H04, H06 |
| ScnDS | September 2018 | B02 |
| ScnAC | October 2018 | B00, B02 |
| ScnAC | November 2018 | B00, B02, B03, B04, B05, B06, G02, G04, G05, G07, G08, G09, G10, G11, H00, H03, H04, H06 |
| ScnAC | December 2018 | B00, B01, B03, B04, B05, B06 |
| ScnAC | February 2019 | B00 |
| ScnAC | March 2019 | B00, G05, G07, G08, G09, G10, G11, H00, H03, H04, H06 |
| ScnAC | April 2019 | B00, B03, B04, B05, B06, G02, G04, G05, G07, G08, G09, G10, H00, H03, H04, H06 |
| ScnAC | May 2019 | B00, G11 |

*S1.2. Determination of micropollutants*

Water samples were taken and stored at -20°C within 6 hours of collection. For sample preparation 2000 µL of thawed sample was mixed with 200µL of methanol and 100 µL of modifier solution, shaken for 30 minutes at high speed using a Heidolph shaker. After centrifugation, 900 µL of supernatant was pipetted into LC-MS vials. 13C standard addition was carried out in all samples and results have been corrected accordingly. The analysis was conducted using a Agilent 6420 Triple Quadrupole LC-MS/MS system with an electrospray ion source. A thorough description of the analysis has been reported elsewhere (omitted author unpublished manuscript). For this study, four compounds have been added to the method later. The mass/charge per compound and recovery rates are listed in Table S9 and Table S10, respectively.

**TABLE S9:** Mass/charge per compound.

| Compound | Precursor ion | Product ion | Retention time (min) | Fragmentor voltage (V) | Collision energy (V) | Polarity |
| --- | --- | --- | --- | --- | --- | --- |
| Metoprolol | 268.2 | 191 | 4.75 | 125 | 15 | Positive |
| Metoprolol | 268.2 | 116 | 4.75 | 125 | 16 | Positive |
| Carbamazepine | 237.2 | 194.2 | 5.83 | 155 | 16 | Positive |
| Carbamazepine | 237.2 | 179.1 | 5.83 | 155 | 40 | Positive |
| Naproxen | 231 | 185 | 6.48 | 90 | 10 | Positive |
| Naproxen | 231 | 170 | 6.48 | 90 | 28 | Positive |
| Diclofenac | 296 | 215 | 7.22 | 95 | 17 | Positive |
| Diclofenac | 296 | 214 | 7.22 | 85 | 32 | Positive |

**TABLE S10:** Compound recovery rates. Recoveries for the measured compounds varied between 70-136 %. Recoveries were determined individually for each samples to cancel any variations do to the water matrix.

| Compound | Mean recovery (standard deviation) [%] |
| --- | --- |
| Carbamazepine | 91.50 (27.19) |
| Ciprofloxacin | 77.52 (32.60) |
| Diclofenac | 136.00 (34.04) |
| Erythromycin | 85.93 (30.55) |
| Metformin | 77.60 (29.35) |
| Metoprolol | 90.88 (27.04) |

*S1.3. Baseline for ‘benchmarking’*

To provide a reliable baseline for the ‘benchmarking’ approach, predicted carbamazepine concentrations (C_pred_ [ng/L]) were first evaluated by comparison with measured concentrations (C_meas_ [ng/L]). To make predicted and measured concentrations comparable, concentration data from monitoring sites where daily flow rates (Q_meas_ [m³/d]) were available were adjusted (C_adj_ [ng/L]) to the flow rate used in the model simulation (Q_model_ [m³/d]),

|  |  | $C_{adj}= C_{meas}\times\frac{Q_{meas}}{Q_{model}}$ |  |  |
| --- | --- | --- | --- | --- |

**S2. SUPPLEMENTARY**

**TABLE S11:** Literature studies retrieved from Web of Science Core Collection (‘Topic’ search mode).

| *Data*  *extracted?* | *Reference* |
| --- | --- |
| NO | Aaen, S. M., & Horsberg, T. E. (2016). A screening of multiple classes of pharmaceutical compounds for effect on preadult salmon lice Lepeophtheirus salmonis. Journal of Fish Diseases, 39(10), 1213-1223. doi:10.1111/jfd.12463 |
| YES | Aderemi, A. O., Novais, S. C., Lemos, M. F. L., Alves, L. M., Hunter, C., & Pahl, O. (2018). Oxidative stress responses and cellular energy allocation changes in microalgae following exposure to widely used human antibiotics. Aquatic Toxicology, 203, 130-139. doi:10.1016/j.aquatox.2018.08.008 |
| NO | Affek, K., Zaleska-Radziwill, M., Doskocz, N., & Debek, K. (2018). Mixture toxicity of pharmaceuticals present in wastewater to aquatic organisms. Desalination and Water Treatment, 117, 15-20. doi:10.5004/dwt.2018.21964 |
| NO | Ajima, M. N. O., Pandey, P. K., Kumar, K., & Poojary, N. (2017). Neurotoxic effects, molecular responses and oxidative stress biomarkers in Nile tilapia, Oreochromis niloticus (Linnaeus, 1758) exposed to verapamil. Comparative Biochemistry and Physiology C-Toxicology & Pharmacology, 196, 44-52. doi:10.1016/j.cbpc.2017.03.009 |
| NO | Alfei, S., Catena, S., Ponassi, M., Rosano, C., Zoppi, V., & Spallarossa, A. (2018). Hydrophilic and amphiphilic water-soluble dendrimer prodrugs suitable for parenteral administration of a non-soluble non-nucleoside HIV-1 reverse transcriptase inhibitor thiocarbamate derivative. European Journal of Pharmaceutical Sciences, 124, 153-164. doi:10.1016/j.ejps.2018.08.036 |
| NO | Alimba, C. G., Adekoya, K. O., & Soyinka, O. O. (2019). Exposure to effluent from pharmaceutical industry induced cytogenotoxicity, hematological and histo-pathological alterations in clarias gariepinus (Burchell, 1822). Excli Journal, 18, 63-78. doi:10.17179/excli2018-1916 |
| NO | Almeida, A. R., Jesus, F., Henriques, J. F., Andrade, T. S., Barreto, A., Koba, O., . . . Domingues, I. (2019). The role of humic acids on gemfibrozil toxicity to zebrafish embryos. Chemosphere, 220, 556-564. doi:10.1016/j.chemosphere.2018.12.133 |
| NO | Al-Saeedi, A. H., Al-Ghafri, M. T. H., & Hossain, M. A. (2017). Brine shrimp toxicity of various polarities leaves and fruits crude fractions Ziziphus jujuba native to Oman and their antimicrobial potency. Sustainable Chemistry and Pharmacy, 5, 122-126. doi:10.1016/j.scp.2017.03.003 |
| NO | Alyahya, S. A., Govindarajan, M., Alharbi, N. S., Kadaikunnan, S., Khaled, J. M., Mothana, R. A., . . . Benelli, G. (2018). Swift fabrication of Ag nanostructures using a colloidal solution of Holostemma ada-kodien (Apocynaceae) - Antibiofilm potential, insecticidal activity against mosquitoes and non-target impact on water bugs. Journal of Photochemistry and Photobiology B-Biology, 181, 70-79. doi:10.1016/j.jphotobiol.2018.02.019 |
| NO | Ashajyothi, C., Handral, H. K., & Kelmani, R. C. (2018). A Comparative In Vivo Scrutiny of Biosynthesized Copper and Zinc Oxide Nanoparticles by Intraperitoneal and Intravenous Administration Routes in Rats. Nanoscale Research Letters, 13. doi:10.1186/s11671-018-2497-2 |
| NO | Backhaus, T. (2016). Environmental Risk Assessment of Pharmaceutical Mixtures: Demands, Gaps, and Possible Bridges. Aaps Journal, 18(4), 804-813. doi:10.1208/s12248-016-9907-0 |
| YES | Baek, I. H., Kim, Y., Baik, S., & Kim, J. (2019). Investigation of the Synergistic Toxicity of Binary Mixtures of Pesticides and Pharmaceuticals on Aliivibrio fischeri in Major River Basins in South Korea. International Journal of Environmental Research and Public Health, 16(2). doi:10.3390/ijerph16020208 |
| NO | Balkrishna, A., Sharma, N., Sharma, V. K., Mishra, N. D., & Joshi, C. S. (2018). Green synthesis, characterisation and biological studies of AgNPs prepared using Shivlingi (Bryonia laciniosa) seed extract. Iet Nanobiotechnology, 12(3), 371-375. doi:10.1049/iet-nbt.2017.0099 |
| NO | Bampidis, V., Azimonti, G., Bastos, M. D., Christensen, H., Dusemund, B., Kouba, M., . . . Subst, E. P. A. P. (2019). Safety and efficacy of Deccox((R)) (decoquinate) for chickens for fattening. Efsa Journal, 17(1). doi:10.2903/j.efsa.2019.5541 |
| NO | Bandeira, G., Pes, T. S., Saccol, E. M. H., Sutili, F. J., Rossi, W., Murari, A. L., . . . Baldisserotto, B. (2017). Potential uses of Ocimum gratissimum and Hesperozygis ringens essential oils in aquaculture. Industrial Crops and Products, 97, 484-491. doi:10.1016/j.indcrop.2016.12.040 |
| NO | Banumathi, B., Vaseeharan, B., Ishwarya, R., Govindarajan, M., Alharbi, N. S., Kadaikunnan, S., . . . Benelli, G. (2017). Toxicity of herbal extracts used in ethno-veterinary medicine and green-encapsulated ZnO nanoparticles against Aedes aegypti and microbial pathogens. Parasitology Research, 116(6), 1637-1651. doi:10.1007/s00436-017-5438-6 |
| NO | Benelli, G., Govindarajan, M., AlSalhi, M. S., Devanesan, S., & Maggi, F. (2018). High toxicity of camphene and gamma-elemene from Wedelia prostrata essential oil against larvae of Spodoptera litura (Lepidoptera: Noctuidae). Environmental Science and Pollution Research, 25(11), 10383-10391. doi:10.1007/s11356-017-9490-7 |
| NO | Benelli, G., Govindarajan, M., Senthilmurugan, S., Vijayan, P., Kadaikunnan, S., Alharbi, N. S., & Khaled, J. M. (2018). Fabrication of highly effective mosquito nanolarvicides using an Asian plant of ethno-pharmacological interest, Priyangu (Aglaia elaeagnoidea): toxicity on non-target mosquito natural enemies. Environmental Science and Pollution Research, 25(11), 10283-10293. doi:10.1007/s11356-017-8898-4 |
| NO | Benelli, G., Pavela, R., Drenaggi, E., & Maggi, F. (2019). Insecticidal efficacy of the essential oil of jambe (Acmella oleracea (L.) RK Jansen) cultivated in central Italy against filariasis mosquito vectors,.pdaus Chock for houseflies and moth pests. Journal of Ethnopharmacology, 229, 272-279. doi:10.1016/j.jep.2018.08.030 |
| YES | Bi, R., Zeng, X. F., Mu, L., Hou, L. P., Liu, W. H., Li, P., . . . Xie, L. T. (2018). Sensitivities of seven algal species to triclosan, fluoxetine and their mixtures. Scientific Reports, 8. doi:10.1038/s41598-018-33785-1 |
| YES | Bialk-Bielinska, A., Mulkiewicz, E., Stokowski, M., Stolte, S., & Stepnowski, P. (2017). Acute aquatic toxicity assessment of six anti-cancer drugs and one metabolite using biotest battery-Biological effects and stability under test conditions. Chemosphere, 189, 689-698. doi:10.1016/j.chemosphere.2017.08.174 |
| YES | Bittner, L., Teixido, E., Seiwert, B., Escher, B. I., & Kluver, N. (2018). Influence of pH on the uptake and toxicity of beta-blockers in embryos of zebrafish, Danio rerio. Aquatic Toxicology, 201, 129-137. doi:10.1016/j.aquatox.2018.05.020 |
| YES | Bohdziewicz, J., Dudziak, M., Kaminska, G., & Kudlek, E. (2016). Chromatographic determination and toxicological potential evaluation of selected micropollutants in aquatic environment-analytical problems. Desalination and Water Treatment, 57(3), 1361-1369. doi:10.1080/19443994.2015.1017325 |
| YES | Borecka, M., Bialk-Bielinska, A., Halinski, L. P., Pazdro, K., Stepnowski, P., & Stolte, S. (2016). The influence of salinity on the toxicity of selected sulfonamides and trimethoprim towards the green algae Chlorella vulgaris. Journal of Hazardous Materials, 308, 179-186. doi:10.1016/j.jhazmat.2016.01.041 |
| NO | Bosker, T., Santoro, G., & Melvin, S. D. (2017). Salinity and sensitivity to endocrine disrupting chemicals: A comparison of reproductive endpoints in small-bodied fish exposed under different salinities. Chemosphere, 183, 186-196. doi:10.1016/j.chemosphere.2017.05.063 |
| NO | Brienza, M., Ahmed, M. M., Escande, A., Plantard, G., Scrano, L., Chiron, S., . . . Goetz, V. (2016). Use of solar advanced oxidation processes for wastewater treatment: Follow-up on degradation products, acute toxicity, genotoxicity and estrogenicity. Chemosphere, 148, 473-480. doi:10.1016/j.chemosphere.2016.01.070 |
| NO | Bueno, F., Borba, F. H., Pellenz, L., Schmitz, M., Godoi, B., Espinoza-Quinones, F. R., . . . Modenes, A. N. (2018). Degradation of ciprofloxacin by the Electrochemical Peroxidation process using stainless steel electrodes. Journal of Environmental Chemical Engineering, 6(2), 2855-2864. doi:10.1016/j.jece.2018.04.033 |
| YES | Bundschuh, M., Hahn, T., Ehrlich, B., Holtge, S., Kreuzig, R., & Schulz, R. (2016). Acute Toxicity and Environmental Risks of Five Veterinary Pharmaceuticals for Aquatic Macroinvertebrates. Bulletin of Environmental Contamination and Toxicology, 96(2), 139-143. doi:10.1007/s00128-015-1656-8 |
| YES | Caldwell, D. J., D'Aco, V., Davidson, T., Kappler, K., Murray-Smith, R. J., Owen, S. F., . . . Tell, J. (2019). Environmental risk assessment of metformin and its transformation product guanylurea: II. Occurrence in surface waters of Europe and the United States and derivation of predicted no-effect concentrations. Chemosphere, 216, 855-865. doi:10.1016/j.chemosphere.2018.10.038 |
| YES | Capolupo, M., Diaz-Garduno, B., & Martin-Diaz, M. L. (2018). The impact of propranolol, 17-ethinylestradiol, and gemfibrozil on early life stages of marine organisms: effects and risk assessment. Environmental Science and Pollution Research, 25(32), 32196-32209. doi:10.1007/s11356-018-3185-6 |
| NO | Cartagena, A. F., Esmerino, L. A., Polak, R., Parreiras, S. O., Michel, M. D., Farago, P. V., & Campanha, N. H. (2017). New denture adhesive containing miconazole nitrate polymeric microparticles: Antifungal, adhesive force and toxicity properties. Dental Materials, 33(2), E53-E61. doi:10.1016/j.dental.2016.09.039 |
| NO | Carty, D. R., Thornton, C., Gledhill, J. H., & Willett, K. L. (2018). Developmental Effects of Cannabidiol and Delta(9)-Tetrahydrocannabinol in Zebrafish. Toxicological Sciences, 162(1), 137-145. doi:10.1093/toxsci/kfx232 |
| YES | Cesen, M., Elersek, T., Novak, M., Zegura, B., Kosjek, T., Filipic, M., & Heath, E. (2016). Ecotoxicity and genotoxicity of cyclophosphamide, ifosfamide, their metabolites/transformation products and their mixtures. Environmental Pollution, 210, 192-201. doi:10.1016/j.envpol.2015.12.017 |
| YES | Chen, H. H., Gu, X. H., Zeng, Q. F., & Mao, Z. G. (2019). Acute and Chronic Toxicity of Carbamazepine on the Release of Chitobiase, Molting, and Reproduction in Daphnia similis. International Journal of Environmental Research and Public Health, 16(2). doi:10.3390/ijerph16020209 |
| YES | Chiffre, A., Clerandeau, C., Dwoinikoff, C., Le Bihanic, F., Budzinski, H., Geret, F., & Cachot, J. (2016). Psychotropic drugs in mixture alter swimming behaviour of Japanese medaka (Oryzias latipes) larvae above environmental concentrations. Environmental Science and Pollution Research, 23(6), 4964-4977. doi:10.1007/s11356-014-3477-4 |
| NO | Chunduri, L. A. A., Kurdekar, A., Patnaik, S., Dev, B. V., Rattan, T. M., & Kamisetti, V. (2016). Carbon Quantum Dots from Coconut Husk: Evaluation for Antioxidant and Cytotoxic Activity. Materials Focus, 5(1), 55-61. doi:10.1166/mat.2016.1289 |
| NO | Cil, O., Phuan, P. W., Lee, S., Tan, J., Haggie, P. M., Levin, M. H., . . . Verkman, A. S. (2016). CFTR Activator Increases Intestinal Fluid Secretion and Normalizes Stool Output in a Mouse Model of Constipation. Cellular and Molecular Gastroenterology and Hepatology, 2(3), 317-327. doi:10.1016/j.jcmgh.2015.12.010 |
| NO | Clausen, L. P. W., & Trapp, S. (2017). Toxicity of 56 substances to trees. Environmental Science and Pollution Research, 24(22), 18035-18047. doi:10.1007/s11356-017-9398-2 |
| NO | Cui, F., Chai, T. T., Qian, L., & Wang, C. J. (2017). Effects of three diamides (chlorantraniliprole, cyantraniliprole and flubendiamide) on life history, embryonic development and oxidative stress biomarkers of Daphnia magna. Chemosphere, 169, 107-116. doi:10.1016/j.chemosphere.2016.11.073 |
| NO | Cunha, D. L., Mendes, M. P., & Marques, M. (2019). Environmental risk assessment of psychoactive drugs in the aquatic environment. Environmental Science and Pollution Research, 26(1), 78-90. doi:10.1007/s11356-018-3556-z |
| YES | Czarny, K., Szczukocki, D., Krawczyk, B., Skrzypek, S., Miekos, E., & Gadzala-Kopciuch, R. (2019). Inhibition of growth of Anabaena variabilis population by single and mixed steroid hormones. Journal of Applied Phycology, 31(1), 389-398. doi:10.1007/s10811-018-1589-9 |
| NO | da Silva, L. D., Gozzi, F., Sires, I., Brillas, E., de Oliveira, S. C., & Machulek, A. (2018). Degradation of 4-aminoantipyrine by electro-oxidation with a boron-doped diamond anode: Optimization by central composite design, oxidation products and toxicity. Science of the Total Environment, 631-632, 1079-1088. doi:10.1016/j.scitotenv.2018.03.092 |
| NO | Dalhoff, K., Gottardi, M., Kretschmann, A., & Cedergreen, N. (2016). What causes the difference in synergistic potentials of propiconazole and prochloraz toward pyrethroids in Daphnia magna? Aquatic Toxicology, 172, 95-102. doi:10.1016/j.aquatox.2015.12.007 |
| NO | Dambal, V. Y., Selvan, K. P., Lite, C., Barathi, S., & Santosh, W. (2017). Developmental toxicity and induction of vitellogenin in embryo-larval stages of zebrafish (Danio rerio) exposed to methyl Paraben. Ecotoxicology and Environmental Safety, 141, 113-118. doi:10.1016/j.ecoenv.2017.02.048 |
| NO | Dar, A. A., Wang, X. H., Wang, S. Y., Ge, J. L., Shad, A., Ai, F. X., & Wang, Z. Y. (2019). Ozonation of pentabromophenol in aqueous basic medium: Kinetics, pathways, mechanism, dimerization and toxicity assessment. Chemosphere, 220, 546-555. doi:10.1016/j.chemosphere.2018.12.154 |
| NO | Dash, R., Bin Emran, T., Paul, A., Siddique, M. K. U., Khan, M. A., Rahman, M. G., . . . Uddin, M. M. N. (2016). Effects of five Bangladeshi plant extracts on In vitro thrombolysis and cytotoxicity. Pharmacognosy Research, 8(3), 176-180. doi:10.4103/0974-8490.181403 |
| NO | Dawson, D. A., & Poch, G. (2017). Evaluation of consistency for multiple experiments of a single combination in the time-dependence mixture toxicity assay. Toxicology Mechanisms and Methods, 27(9), 707-716. doi:10.1080/15376516.2017.1351019 |
| NO | de Farias, N. O., Oliveira, R., Sousa-Moura, D., de Oliveira, R. C. S., Rodrigues, M. A. C., Andrade, T. S., . . . Grisolia, C. K. (2019). Exposure to low concentration of fluoxetine affects development, behaviour and acetylcholinesterase activity of zebrafish embryos. Comparative Biochemistry and Physiology C-Toxicology & Pharmacology, 215, 1-8. doi:10.1016/j.cbpc.2018.08.009 |
| YES | de Garcia, S. O., Garcia-Encina, P. A., & Irusta-Mata, R. (2016). Dose-response behavior of the bacterium Vibrio fischeri exposed to pharmaceuticals and personal care products. Ecotoxicology, 25(1), 141-162. doi:10.1007/s10646-015-1576-8 |
| YES | de Oliveira, L. L. D., Nunes, B., Antunes, S. C., Campitelli-Ramos, R., & Rocha, O. (2018). Acute and Chronic Effects of Three Pharmaceutical Drugs on the Tropical Freshwater Cladoceran Ceriodaphnia silvestrii. Water Air and Soil Pollution, 229(4). doi:10.1007/s11270-018-3765-6 |
| NO | Dechayont, B., Limpichai, C., Kornwisitwathin, K., Nuengchamnong, N., & Itharat, A. (2017). In vitro cytotoxic and antioxidant activities of Pikut Trichinthalamaga remedy. Oriental Pharmacy and Experimental Medicine, 17(3), 233-238. doi:10.1007/s13596-017-0278-6 |
| NO | Destrieux, D., Laurent, F., Budzinski, H., Pedelucq, J., Vervier, P., & Gerino, M. (2017). Drug residues in urban water: A database for ecotoxicological risk management. Science of the Total Environment, 609, 927-941. doi:10.1016/j.scitotenv.2017.07.043 |
| NO | Dharmaratne, M. P. J., Manoraj, A., Thevanesam, V., Ekanayake, A., Kumar, N. S., Liyanapathirana, V., . . . Bandara, B. M. R. (2018). Terminalia bellirica fruit extracts: in-vitro antibacterial activity against selected multidrug-resistant bacteria, radical scavenging activity and cytotoxicity study on BHK-21 cells. Bmc Complementary and Alternative Medicine, 18. doi:10.1186/s12906-018-2382-7 |
| NO | Di Nica, V., Villa, S., & Finizio, A. (2017). Toxicity of individual pharmaceuticals and their mixtures to aliivibrio fischeri: evidence of toxicological interactions in binary combinations. Environmental Toxicology and Chemistry, 36(3), 815-822. doi:10.1002/etc.3686 |
| YES | Di Nica, V., Villa, S., & Finizio, A. (2017). Toxicity of individual pharmaceuticals and their mixtures to aliivibrio fischeri: experimental results for single compounds and considerations of their mechanisms of action and potential acute effects on aquatic organisms. Environmental Toxicology and Chemistry, 36(3), 807-814. doi:10.1002/etc.3568 |
| YES | Di Paolo, C., Ottermanns, R., Keiter, S., Ait-Aissa, S., Bluhm, K., Brack, W., . . . Hollert, H. (2016). Bioassay battery interlaboratory investigation of emerging contaminants in spiked water extracts - Towards the implementation of bioanalytical monitoring tools in water quality assessment and monitoring. Water Research, 104, 473-484. doi:10.1016/j.watres.2016.08.018 |
| YES | Di Poi, C., Costil, K., Bouchart, V., & Halm-Lemeille, M. P. (2018). Toxicity assessment of five emerging pollutants, alone and in binary or ternary mixtures, towards three aquatic organisms. Environmental Science and Pollution Research, 25(7), 6122-6134. doi:10.1007/s11356-017-9306-9 |
| NO | Diamond, J., Altenburger, R., Coors, A., Dyer, S. D., Focazio, M., Kidd, K., . . . Zhang, X. W. (2018). Use of prospective and retrospective risk assessment methods that simplify chemical mixtures associated with treated domestic wastewater discharges. Environmental Toxicology and Chemistry, 37(3), 690-702. doi:10.1002/etc.4013 |
| NO | Ding, T. D., Lin, K. D., Chen, J., Hu, Q., Yang, B., Li, J. Y., & Gan, J. (2018). Causes and mechanisms on the toxicity of layered double hydroxide (LDH) to green algae Scenedesmus quadricauda. Science of the Total Environment, 635, 1004-1011. doi:10.1016/j.scitotenv.2018.04.222 |
| YES | Ding, T. D., Lin, K. D., Yang, M. T., Bao, L. J., Li, J. Y., Yang, B., & Gan, J. (2018). Biodegradation of triclosan in diatom Navicula sp.: Kinetics, transformation products, toxicity evaluation and the effects of pH and potassium permanganate. Journal of Hazardous Materials, 344, 200-209. doi:10.1016/j.jhazmat.2017.09.033 |
| NO | Dogan, S., & Kidak, R. (2016). A Plug flow reactor model for UV-based oxidation of amoxicillin. Desalination and Water Treatment, 57(29), 13586-13599. doi:10.1080/19443994.2015.1058728 |
| NO | Donnachie, R. L., Johnson, A. C., & Sumpter, J. P. (2016). A rational approach to selecting and ranking some pharmaceuticals of concern for the aquatic environment and their relative importance compared with other chemicals. Environmental Toxicology and Chemistry, 35(4), 1021-1027. doi:10.1002/etc.3165 |
| NO | Drobniewska, A., Wojcik, D., Kapan, M., Adomas, B., Piotrowicz-Cieslak, A., & Nalecz-Jawecki, G. (2017). Recovery of Lemna minor after exposure to sulfadimethoxine irradiated and non-irradiated in a solar simulator. Environmental Science and Pollution Research, 24(36), 27642-27652. doi:10.1007/s11356-016-7174-3 |
| YES | Du, J., Mei, C. F., Ying, G. G., & Xu, M. Y. (2016). Toxicity Thresholds for Diclofenac, Acetaminophen and Ibuprofen in the Water Flea Daphnia magna. Bulletin of Environmental Contamination and Toxicology, 97(1), 84-90. doi:10.1007/s00128-016-1806-7 |
| NO | Elersek, T., Milavec, S., Korosec, M., Brezovsek, P., Negreira, N., Zonja, B., . . . Filipic, M. (2016). Toxicity of the mixture of selected antineoplastic drugs against aquatic primary producers. Environmental Science and Pollution Research, 23(15), 14780-14790. doi:10.1007/s11356-015-6005-2 |
| NO | Eltahan, R., Guo, F. G., Zhang, H. L., Xiang, L. X., & Zhu, G. (2018). Discovery of ebselen as an inhibitor of Cryptosporidium parvum glucose-6-phosphate isomerase (CpGPI) by high-throughput screening of existing drugs. International Journal for Parasitology-Drugs and Drug Resistance, 8(1), 43-49. doi:10.1016/j.ijpddr.2018.01.003 |
| NO | Estevez-Calvar, N., Canesi, L., Montagna, M., Faimali, M., Piazza, V., & Garaventa, F. (2017). Adverse effects of the SSRI antidepressant sertraline on early life stages of marine invertebrates. Marine Environmental Research, 128, 88-97. doi:10.1016/j.marenvres.2016.05.021 |
| YES | Fekete-Kertesz, I., Ullmann, O., Csizmar, P., & Molnar, M. (2018). Tetrahymena pyriformis Phagocytic Activity Test for Rapid Toxicity Assessment of Aquatic Micropollutants. Periodica Polytechnica-Chemical Engineering, 62(2), 167-174. doi:10.3311/PPch.10667 |
| NO | Felix, L. M., Serafim, C., Martins, M. J., Valentim, A. M., Antunes, L. M., Matos, M., & Coimbra, A. M. (2017). Morphological and behavioral responses of zebrafish after 24 h of ketamine embryonic exposure. Toxicology and Applied Pharmacology, 321, 27-36. doi:10.1016/j.taap.2017.02.013 |
| NO | Fonte, E., Ferreira, P., & Guilhermino, L. (2016). Temperature rise and microplastics interact with the toxicity of the antibiotic cefalexin to juveniles of the common goby (Pomatoschistus microps): Post-exposure predatory behaviour, acetylcholinesterase activity and lipid peroxidation. Aquatic Toxicology, 180, 173-185. doi:10.1016/j.aquatox.2016.09.015 |
| NO | Forsatkar, M. N., HedayatiRad, M., & Luchiari, A. C. (2018). "Not tonight zebrafish": the effects of Ruta graveolens on reproduction. Pharmaceutical Biology, 56(1), 60-66. doi:10.1080/13880209.2017.1421234 |
| NO | Geetha, V., Sujata, R., Shreenidhi, K. S., & Sundararaman, T. R. (2018). Histopathological and HPLC Analysis in the Hepatic Tissue of Pangasius sp Exposed to Diclofenac. Polish Journal of Environmental Studies, 27(6), 2493-2498. doi:10.15244/pjoes/75829 |
| YES | Geiss, C., Ruppert, K., Heidelbach, T., & Oehlmann, J. (2016). The antimicrobial agents triclocarban and triclosan as potent modulators of reproduction in Potamopyrgus antipodarum (Mollusca: Hydrobiidae). Journal of Environmental Science and Health Part a-Toxic/Hazardous Substances & Environmental Engineering, 51(13), 1173-1179. doi:10.1080/10934529.2016.1206388 |
| YES | Gheorghe, S., Petre, J., Lucaciu, I., Stoica, C., & Nita-Lazar, M. (2016). Risk screening of pharmaceutical compounds in Romanian aquatic environment. Environmental Monitoring and Assessment, 188(6). doi:10.1007/s10661-016-5375-3 |
| YES | Gilroy, E. A. M., Gillis, P. L., King, L. E., Bendo, N. A., Salerno, J., Giacomin, M., & de Sollaz, S. R. (2017). The effects of pharmaceuticals on a unionid mussel (Lampsilis siliquoidea): an examination of acute and chronic endpoints of toxicity across life stages. Environmental Toxicology and Chemistry, 36(6), 1572-1583. doi:10.1002/etc.3683 |
| YES | Godoy, A. A., Domingues, I., Nogueira, A. J. A., & Kummrow, F. (2018). Ecotoxicological effects, water quality standards and risk assessment for the anti-diabetic metformin. Environmental Pollution, 243, 534-542. doi:10.1016/j.envpol.2018.09.031 |
| YES | Gonzalez-Perez, B. K., Sarma, S. S. S., Castellanos-Paez, M. E., & Nandini, S. (2018). Multigenerational effects of triclosan on the demography of Plationus patulus and Brachionus havanaensis (ROTIFERA). Ecotoxicology and Environmental Safety, 147, 275-282. doi:10.1016/j.ecoenv.2017.08.049 |
| NO | Gosset, A., Durrieu, C., Orias, F., Bayard, R., & Perrodin, Y. (2017). Identification and assessment of ecotoxicological hazards attributable to pollutants in urban wet weather discharges. Environmental Science-Processes & Impacts, 19(9), 1150-1168. doi:10.1039/c7em00159b |
| NO | Graca, V. C., Barros, L., Calhelha, R. C., Dias, M. I., Ferreira, I., & Santos, P. F. (2017). Bio-guided fractionation of extracts of Geranium robertianum L.: Relationship between phenolic profile and biological activity. Industrial Crops and Products, 108, 543-552. doi:10.1016/j.indcrop.2017.07.016 |
| NO | Grill, G., Li, J., Khan, U., Zhong, Y., Lehner, B., Nicell, J., & Ariwi, J. (2018). Estimating the eco-toxicological risk of estrogens in China's rivers using a high-resolution contaminant fate model. Water Research, 145, 707-720. doi:10.1016/j.watres.2018.08.053 |
| NO | Grzesiuk, M., Wacker, A., & Spijkerman, E. (2016). Photosynthetic sensitivity of phytoplankton to commonly used pharmaceuticals and its dependence on cellular phosphorus status. Ecotoxicology, 25(4), 697-707. doi:10.1007/s10646-016-1628-8 |
| NO | Guo, J. H., Selby, K., & Boxall, A. B. A. (2016). Assessment of the Risks of Mixtures of Major Use Veterinary Antibiotics in European Surface Waters. Environmental Science & Technology, 50(15), 8282-8289. doi:10.1021/acs.est.6b01649 |
| YES | Guo, J. H., Selby, K., & Boxall, A. B. A. (2016). Comparing the sensitivity of chlorophytes, cyanobacteria, and diatoms to major-use antibiotics. Environmental Toxicology and Chemistry, 35(10), 2587-2596. doi:10.1002/etc.3430 |
| NO | Guo, J. H., Selby, K., & Boxall, A. B. A. (2016). Effects of Antibiotics on the Growth and Physiology of Chlorophytes, Cyanobacteria, and a Diatom. Archives of Environmental Contamination and Toxicology, 71(4), 589-602. doi:10.1007/s00244-016-0305-5 |
| NO | Hamilton, K. D., Brooks, P. R., Ogbourne, S. M., & Russell, F. D. (2017). Natural products isolated from Tetragonula carbonaria cerumen modulate free radical-scavenging and 5-lipoxygenase activities in vitro. Bmc Complementary and Alternative Medicine, 17. doi:10.1186/s12906-017-1748-6 |
| NO | Harbi, K., Makridis, P., Koukoumis, C., Papadionysiou, M., Vgenis, T., Kornaros, M., . . . Dailianis, S. (2017). Evaluation of a battery of marine species-based bioassays against raw and treated municipal wastewaters. Journal of Hazardous Materials, 321, 537-546. doi:10.1016/j.jhazmat.2016.09.036 |
| NO | Heidari-Kharaji, M., Fallah-Omrani, V., Badirzadeh, A., Mohammadi-Ghalehbin, B., Nilforoushzadeh, M. A., Masoori, L., . . . Zare, M. (2019). Sambucus ebulus extract stimulates cellular responses in cutaneous leishmaniasis. Parasite Immunology, 41(1). doi:10.1111/pim.12605 |
| NO | Henriques, J. F., Almeida, A. R., Andrade, T., Koba, O., Golovko, O., Soares, A., . . . Domingues, I. (2016). Effects of the lipid regulator drug gemfibrozil: A toxicological and behavioral perspective. Aquatic Toxicology, 170, 355-364. doi:10.1016/j.aquatox.2015.09.017 |
| YES | Heye, K., Becker, D., Eversloh, C. L., Durmaz, V., Ternes, T. A., Oetken, M., & Oehlmann, J. (2016). Effects of carbamazepine and two of its metabolites on the non-biting midge Chironomus riparius in a sediment full life cycle toxicity test. Water Research, 98, 19-27. doi:10.1016/j.watres.2016.03.071 |
| NO | Hok, L., Ulm, L., Tandaric, T., Krivohlavek, A., Sakic, D., & Vrcek, V. (2018). Chlorination of 5-fluorouracil: Reaction mechanism and ecotoxicity assessment of chlorinated products. Chemosphere, 207, 612-619. doi:10.1016/j.chemosphere.2018.05.140 |
| NO | Huang, B. S., Chen, W. M., Zhao, T., Li, Z. Y., Jiang, X. Y., Ginex, T., . . . Liu, X. Y. (2019). Exploiting the Tolerant Region I of the Non-Nucleoside Reverse Transcriptase Inhibitor (NNRTI) Binding Pocket: Discovery of Potent Diarylpyrimidine-Typed HIV-1 NNRTIs against Wild-Type and E138K Mutant Virus with Significantly Improved Water Solubility and Favorable Safety Profiles. Journal of Medicinal Chemistry, 62(4), 2083-2098. doi:10.1021/acs.jmedchem.8b01729 |
| NO | Huang, Q. S., Bu, Q. W., Zhong, W. J., Shi, K. C., Cao, Z. G., & Yu, G. (2018). Derivation of aquatic predicted no-effect concentration (PNEC) for ibuprofen and sulfamethoxazole based on various toxicity endpoints and the associated risks. Chemosphere, 193, 223-229. doi:10.1016/j.chemosphere.2017.11.029 |
| NO | Iesce, M. R., Lavorgna, M., Russo, C., Piscitelli, C., Passananti, M., Temussi, F., . . . Isidori, M. (2019). Ecotoxic effects of loratadine and its metabolic and light-induced derivatives. Ecotoxicology and Environmental Safety, 170, 664-672. doi:10.1016/j.ecoenv.2018.11.116 |
| NO | Ioele, G., De Luca, M., & Ragno, G. (2016). Acute Toxicity of Antibiotics in Surface Waters by Bioluminescence Test. Current Pharmaceutical Analysis, 12(3), 220-226. doi:10.2174/1573412912666151110204041 |
| NO | Jainab, N. H., & Raja, M. (2017). In vitro cytotoxic, antioxidant and gc-ms study of leaf extracts of clerodendrum phlomidis. International Journal of Pharmaceutical Sciences and Research, 8(10), 4433-4440. doi:10.13040/ijpsr.0975-8232.8(10).4433-40 |
| NO | Jamil, S., Khan, R. A., Afroz, S., & Ahmed, S. (2016). Phytochemistry, Brine shrimp lethality and mice acute oral toxicity studies on seed extracts of Vernonia anthelmintica. Pakistan Journal of Pharmaceutical Sciences, 29(6), 2053-2057. |
| YES | Jungmann, D., Berg, K., Dieterich, A., Frank, M., Graf, T., Scheurer, M., . . . Oetken, M. (2017). Health effects of metoprolol in epibenthic and endobenthic invertebratesA basis to validate future in vitro biotests for effect-based biomonitoring. Journal of Environmental Science and Health Part a-Toxic/Hazardous Substances & Environmental Engineering, 52(3), 189-200. doi:10.1080/10934529.2016.1246930 |
| NO | Jureczko, M., & Przystas, W. (2019). Ecotoxicity risk of presence of two cytostatic drugs: Bleomycin and vincristine and their binary mixture in aquatic environment. Ecotoxicology and Environmental Safety, 172, 210-215. doi:10.1016/j.ecoenv.2019.01.074 |
| NO | Kanwar, R., Kaur, G., & Mehta, S. K. (2016). Revealing the potential of Didodecyldimethylammonium bromide as efficient scaffold for fabrication of nano liquid crystalline structures. Chemistry and Physics of Lipids, 196, 61-68. doi:10.1016/j.chemphyslip.2016.02.006 |
| YES | Karaaslan, M. A., & Parlak, H. (2016). The embryotoxic and genotoxic effects of widely used beta blockers on sea urchin (Paracentrotus lividus) embryos. Fresenius Environmental Bulletin, 25(12A), 6100-6105. |
| NO | Kaska, A., Cicek, M., Deniz, N., & Mammadov, R. (2018). Investigation of Phenolic Content, Antioxidant Capacities, Anthelmintic and Cytotoxic Activities of Thymus zygioides Griseb. Journal of Pharmaceutical Research International, 21(1). doi:10.9734/jpri/2018/39688 |
| NO | Kilonzo, M., Ndakidemi, P. A., & Chacha, M. (2016). In vitro antifungal and cytotoxicity activities of selected Tanzanian medicinal plants. Tropical Journal of Pharmaceutical Research, 15(10), 2121-2130. doi:10.4314/tjpr.v15i10.10 |
| NO | Kostich, M. S., Flick, R. W., Batt, A. L., Mash, H. E., Boone, J. S., Furlong, E. T., . . . Glassmeyer, S. T. (2017). Aquatic concentrations of chemical analytes compared to ecotoxicity estimates. Science of the Total Environment, 579, 1649-1657. doi:10.1016/j.scitotenv.2016.06.234 |
| NO | Kovacs, R., Bakos, K., Urbanyi, B., Kovesi, J., Gazsi, G., Csepeli, A., . . . Horvath, A. (2016). Acute and sub-chronic toxicity of four cytostatic drugs in zebrafish. Environmental Science and Pollution Research, 23(15), 14718-14729. doi:10.1007/s11356-015-5036-z |
| YES | Kudlak, B., Wieczerzak, M., & Namiesnik, J. (2018). Determination of toxicological parameters of selected bioactive organic chemicals using the ostracodtoxkit f (tm). Chemistry-Didactics-Ecology-Metrology, 23(1-2), 113-126. doi:10.1515/cdem-2018-0007 |
| NO | Kumar, D., Kumar, G., Das, R., & Agrawal, V. (2018). Strong larvicidal potential of silver nanoparticles (AgNPs) synthesized using Holarrhena antidysenterica (L.) Wall. bark extract against malarial vector, Anopheles stephensi Liston. Process Safety and Environmental Protection, 116, 137-148. doi:10.1016/j.psep.2018.02.001 |
| NO | Kumar, V. A., Ammani, K., Jobina, R., Subhaswaraj, P., & Siddhardha, B. (2017). Photo-induced and phytomediated synthesis of silver nanoparticles using Derris trifoliata leaf extract and its larvicidal activity against Aedes aegypti. Journal of Photochemistry and Photobiology B-Biology, 171, 1-8. doi:10.1016/j.jphotobiol.2017.04.022 |
| NO | Kwak, K., Ji, K., Kho, Y., Kim, P., Lee, J., Ryu, J., & Choi, K. (2018). Chronic toxicity and endocrine disruption of naproxen in freshwater waterfleas and fish, and steroidogenic alteration using H295R cell assay. Chemosphere, 204, 156-162. doi:10.1016/j.chemosphere.2018.04.035 |
| NO | Lajmanovich, R. C., Peltzer, P. M., Martinuzzi, C. S., Attademo, A. M., Colussi, C. L., & Basso, A. (2018). Acute Toxicity of Colloidal Silicon Dioxide Nanoparticles on Amphibian Larvae: Emerging Environmental Concern. International Journal of Environmental Research, 12(3), 269-278. doi:10.1007/s41742-018-0089-8 |
| NO | Leng, K. M., Vijayarathna, S., Jothy, S. L., Sasidharan, S., & Kanwar, J. R. (2018). In vitro and in vivo toxicity assessment of alginate/eudragit S 100-enclosed chitosan-calcium phosphate-loaded iron saturated bovine lactoferrin nanocapsules (Fe-bLf NCs). Biomedicine & Pharmacotherapy, 97, 26-37. doi:10.1016/j.biopha.2017.10.121 |
| NO | Leporati, A., Novikov, M. S., Valuev-Elliston, V. T., Korolev, S. P., Khandazhinskaya, A. L., Kochetkov, S. N., . . . Bogdanov, A. A. (2016). Hydrophobic-core PEGylated graft copolymer-stabilized nanoparticles composed of insoluble non-nucleoside reverse transcriptase inhibitors exhibit strong anti-HIV activity. Nanomedicine-Nanotechnology Biology and Medicine, 12(8), 2405-2413. doi:10.1016/j.nano.2016.07.004 |
| NO | Li, Q., Wang, P. P., Chen, L., Gao, H. W., & Wu, L. L. (2016). Acute toxicity and histopathological effects of naproxen in zebrafish (Danio rerio) early life stages. Environmental Science and Pollution Research, 23(18), 18832-18841. doi:10.1007/s11356-016-7092-4 |
| NO | Li, S. W., Wang, Y. H., & Lin, A. Y. C. (2017). Ecotoxicological effect of ketamine: Evidence of acute, chronic and photolysis toxicity to Daphnia magna. Ecotoxicology and Environmental Safety, 143, 173-179. doi:10.1016/j.ecoenv.2017.05.040 |
| NO | Li, X. H., He, Q. H., Li, H. Y., Gao, X., Hu, M. C., Li, S. N., . . . Wang, X. T. (2017). Bioconversion of non-steroidal anti-inflammatory drugs diclofenac and naproxen by chloroperoxidase. Biochemical Engineering Journal, 120, 7-16. doi:10.1016/j.bej.2016.12.018 |
| NO | Li, X. W., Zhou, S. X., Qian, Y. T., Xu, Z. R., Yu, Y., Xu, Y. H., . . . Zhang, Y. J. (2018). The assessment of the eco-toxicological effect of gabapentin on early development of zebrafish and its antioxidant system. Rsc Advances, 8(40), 22777-22784. doi:10.1039/c8ra04250k |
| NO | Lindim, C., de Zwart, D., Cousins, I. T., Kutsarova, S., Kuhne, R., & Schuurmann, G. (2019). Exposure and ecotoxicological risk assessment of mixtures of top prescribed pharmaceuticals in Swedish freshwaters. Chemosphere, 220, 344-352. doi:10.1016/j.chemosphere.2018.12.118 |
| NO | Liu, Y. M., Zhang, Q. Z., Xu, D. H., Fu, Y. W., Lin, D. J., & Zhou, S. Y. (2017). Antiparasitic efficacy of commercial curcumin against Ichthyophthirius multifiliis in grass carp (Ctenopharyngodon idellus). Aquaculture, 480, 65-70. doi:10.1016/j.aquaculture.2017.07.041 |
| NO | Liu, Y., Junaid, M., Wang, Y., Tang, Y. M., Bian, W. P., Xiong, W. X., . . . Pei, D. S. (2018). New toxicogenetic insights and ranking of the selected pharmaceuticals belong to the three different classes: A toxicity estimation to confirmation approach. Aquatic Toxicology, 201, 151-161. doi:10.1016/j.aquatox.2018.06.008 |
| NO | Lopez-Luna, J., Camacho-Martinez, M. M., Solis-Dominguez, F. A., Gonzalez-Chavez, M. C., Carrillo-Gonzalez, R., Martinez-Vargas, S., . . . Cuevas-Diaz, M. C. (2018). Toxicity assessment of cobalt ferrite nanoparticles on wheat plants. Journal of Toxicology and Environmental Health-Part a-Current Issues, 81(14), 604-619. doi:10.1080/15287394.2018.1469060 |
| NO | Lv, L. Y., Li, W. G., Yu, Y., Meng, L. Q., Qin, W., & Wu, C. D. (2018). Predicting acute toxicity of traditional Chinese medicine wastewater using UV absorption and volatile fatty acids as surrogates. Chemosphere, 194, 211-219. doi:10.1016/j.chemosphere.2017.11.170 |
| NO | Machado, M. D., & Soares, E. V. (2019). Sensitivity of freshwater and marine green algae to three compounds of emerging concern. Journal of Applied Phycology, 31(1), 399-408. doi:10.1007/s10811-018-1511-5 |
| NO | Madikizela, B., & McGaw, L. J. (2018). Scientific rationale for traditional use of plants to treat tuberculosis in the eastern region of the OR Tambo district, South Africa. Journal of Ethnopharmacology, 224, 250-260. doi:10.1016/j.jep.2018.06.002 |
| YES | Magdaleno, A., Carusso, S., & Moretton, J. (2017). Toxicity and Genotoxicity of Three Antimicrobials Commonly Used in Veterinary Medicine. Bulletin of Environmental Contamination and Toxicology, 99(3), 315-320. doi:10.1007/s00128-017-2091-9 |
| YES | Majewska, M., Harshkova, D., Gusciora, M., & Aksmann, A. (2018). Phytotoxic activity of diclofenac: Evaluation using a model green alga Chlamydomonas reinhardtii with atrazine as a reference substance. Chemosphere, 209, 989-997. doi:10.1016/j.chemosphere.2018.06.156 |
| NO | Maranho, L. A., Fontes, M. K., Kamimura, A. S. S., Nobre, C. R., Moreno, B. B., Pusceddu, F. H., . . . Pereira, C. D. S. (2017). Exposure to crack cocaine causes adverse effects on marine mussels Perna perna. Marine Pollution Bulletin, 123(1-2), 410-414. doi:10.1016/j.marpolbul.2017.08.043 |
| NO | Marchiori, N. D., Silva, F. M., Martins, M. L., Amaral, H., & da Silva, B. C. (2017). Hydrogen peroxide and chlorine dioxide against parasite Ichthyophthirius multifiliis (Protozoa, Ciliophora) in jundia fingerlings. Ciencia Rural, 47(12). doi:10.1590/0103-8478cr20170257 |
| NO | Marchlewicz, A., Guzik, U., Hupert-Kocurek, K., Nowak, A., Wilczynska, S., & Wojcieszynska, D. (2017). Toxicity and biodegradation of ibuprofen by Bacillus thuringiensis B1(2015b). Environmental Science and Pollution Research, 24(8), 7572-7584. doi:10.1007/s11356-017-8372-3 |
| NO | Marchlewicz, A., Guzik, U., Smulek, W., & Wojcieszynska, D. (2017). Exploring the Degradation of Ibuprofen by Bacillus thuringiensis B1(2015b): The New Pathway and Factors Affecting Degradation. Molecules, 22(10). doi:10.3390/molecules22101676 |
| NO | Martinez, E., Velez, S. M., Mayo, M., & Sastre, M. P. (2016). Acute toxicity assessment of N,N-diethyl-m-toluamide (DEET) on the oxygen flux of the dinoflagellate Gymnodinium instriatum. Ecotoxicology, 25(1), 248-252. doi:10.1007/s10646-015-1564-z |
| NO | Martino, C., Bonaventura, R., Byrne, M., Roccheri, M., & Matranga, V. (2017). Effects of exposure to gadolinium on the development of geographically and phylogenetically distant sea urchins species. Marine Environmental Research, 128, 98-106. doi:10.1016/j.marenvres.2016.06.001 |
| NO | McKinley, K., McLellan, I., Gagne, F., & Quinn, B. (2019). The toxicity of potentially toxic elements (Cu, Fe, Mn, Zn and Ni) to the cnidarian Hydra attenuata at environmentally relevant concentrations. Science of the Total Environment, 665, 848-854. doi:10.1016/j.scitotenv.2019.02.193 |
| NO | Mennillo, E., Arukwe, A., Monni, G., Meucci, V., Intorre, L., & Pretti, C. (2018). Ecotoxicological Properties of Ketoprofen and the S(+)-Enantiomer (Dexketoprofen): Bioassays in Freshwater Model Species and Biomarkers in Fish PLHC-1 Cell Line. Environmental Toxicology and Chemistry, 37(1), 201-212. doi:10.1002/etc.3943 |
| NO | Menz, J., Baginska, E., Arrhenius, A., Haiss, A., Backhaus, T., & Kummerer, K. (2017). Antimicrobial activity of pharmaceutical cocktails in sewage treatment plant effluent - An experimental and predictive approach to mixture risk assessment. Environmental Pollution, 231, 1507-1517. doi:10.1016/j.envpol.2017.09.009 |
| NO | Menz, J., Muller, J., Olsson, O., & Kummerer, K. (2018). Bioavailability of Antibiotics at Soil-Water Interfaces: A Comparison of Measured Activities and Equilibrium Partitioning Estimates. Environmental Science & Technology, 52(11), 6555-6564. doi:10.1021/acs.est.7b06329 |
| NO | Mesquita, B., Lopes, I., Silva, S., Bessa, M. J., Starykevich, M., Carneiro, J., . . . Fraga, S. (2017). Gold nanorods induce early embryonic developmental delay and lethality in zebrafish (Danio rerio). Journal of Toxicology and Environmental Health-Part a-Current Issues, 80(13-15), 672-687. doi:10.1080/15287394.2017.1331597 |
| NO | Mihaich, E., Staples, C., Ortego, L., Klecka, G., Woelz, J., Dimond, S., & Hentges, S. (2018). Life-Cycle Studies with 2 Marine Species and Bisphenol A: The Mysid Shrimp (Americamysis bahia) and Sheepshead Minnow (Cyprinodon variegatus). Environmental Toxicology and Chemistry, 37(2), 398-410. doi:10.1002/etc.3957 |
| NO | Montalvao, M. F., Sampaio, L. L. G., Gomes, H. H. F., & Malafaia, G. (2019). An insight into the cytotoxicity, genotoxicity, and mutagenicity of smoked cigarette butt leachate by using Allium cepa as test system. Environmental Science and Pollution Research, 26(2), 2013-2021. doi:10.1007/s11356-018-3731-2 |
| NO | Morales-Serna, F. N., Chapa-Lopez, M., Martinez-Brown, J. M., Ibarra-Castro, L., Medina-Guerrero, R. M., & Fajer-Avila, E. J. (2018). Efficacy of praziquantel and a combination anthelmintic (Adecto((R))) in bath treatments against Tagia ecuadori and Neobenedenia melleni (Monogenea), parasites of bullseye puffer fish. Aquaculture, 492, 361-368. doi:10.1016/j.aquaculture.2018.04.043 |
| NO | Murugadas, A., Mahamuni, D., Nirmaladevi, S. D., Thamaraiselvi, K., Thirumurugan, R., & Akbarsha, M. A. (2019). Hydra as an alternative model organism for toxicity testing: Study using the endocrine disrupting chemical Bisphenol A. Biocatalysis and Agricultural Biotechnology, 17, 680-684. doi:10.1016/j.bcab.2019.01.009 |
| NO | Murugan, K., Nataraj, D., Jaganathan, A., Dinesh, D., Jayashanthini, S., Samidoss, C. M., . . . Benelli, G. (2017). Nanofabrication of Graphene Quantum Dots with High Toxicity Against Malaria Mosquitoes, Plasmodium falciparum and MCF-7 Cancer Cells: Impact on Predation of Non-target Tadpoles, Odonate Nymphs and Mosquito Fishes. Journal of Cluster Science, 28(1), 393-411. doi:10.1007/s10876-016-1107-7 |
| NO | Nasir, B., Ahmad, M., Zahra, S. S., Fatima, H., & Ur-Rehman, T. (2017). PHARMACOLOGICAL EVALUATION OF FUMARIA INDICA (HAUSSKN.) PUGSLEY; A TRADITIONALLY IMPORTANT MEDICINAL PLANT. Pakistan Journal of Botany, 49, 119-132. |
| NO | Nasrallah, G. K., Al-Asmakh, M., Rasool, K., & Mahmoud, K. A. (2018). Ecotoxicological assessment of Ti3C2Tx (MXene) using a zebrafish embryo model. Environmental Science-Nano, 5(4), 1002-1011. doi:10.1039/c7en01239j |
| NO | Ncube, S., Madikizela, L. M., Chimuka, L., & Nindi, M. M. (2018). Environmental fate and ecotoxicological effects of antiretrovirals: A current global status and future perspectives. Water Research, 145, 231-247. doi:10.1016/j.watres.2018.08.017 |
| NO | Neal, A. E., & Moore, P. A. (2017). Mimicking natural systems: Changes in behavior as a result of dynamic exposure to naproxen. Ecotoxicology and Environmental Safety, 135, 347-357. doi:10.1016/j.ecoenv.2016.10.015 |
| NO | Nguyen, P. Y., Carvalho, G., Reis, A. C., Nunes, O. C., Reis, M. A. M., & Oehmen, A. (2017). Impact of biogenic substrates on sulfamethoxazole biodegradation kinetics by Achromobacter denitrificans strain PR1. Biodegradation, 28(2-3), 205-217. doi:10.1007/s10532-017-9789-6 |
| NO | Nielsen, M. E., & Roslev, P. (2018). Behavioral responses and starvation survival of Daphnia magna exposed to fluoxetine and propranolol. Chemosphere, 211, 978-985. doi:10.1016/j.chemosphere.2018.08.027 |
| YES | Nieto, E., Hampel, M., Gonzalez-Ortegon, E., Drake, P., & Blasco, J. (2016). Influence of temperature on toxicity of single pharmaceuticals and mixtures, in the crustacean A. desmarestii. Journal of Hazardous Materials, 313, 159-169. doi:10.1016/j.jhazmat.2016.03.061 |
| NO | Njoya, E. M., Eloff, J. N., & McGaw, L. J. (2018). Croton gratissimus leaf extracts inhibit cancer cell growth by inducing caspase 3/7 activation with additional anti-inflammatory and antioxidant activities. Bmc Complementary and Alternative Medicine, 18. doi:10.1186/s12906-018-2372-9 |
| NO | Novoa-Luna, K. A., Mendoza-Zepeda, A., Natividad, R., Romero, R., Galar-Martinez, M., & Gomez-Olivan, L. M. (2016). Biological hazard evaluation of a pharmaceutical effluent before and after a photo-Fenton treatment. Science of the Total Environment, 569, 830-840. doi:10.1016/j.scitotenv.2016.06.086 |
| NO | Novoa-Luna, K. A., Romero-Romero, R., Natividad-Rangel, R., Galar-Martinez, M., SanJuan-Reyes, N., Garcia-Medina, S., . . . Gomez-Olivan, L. M. (2016). Oxidative stress induced in Hyalella azteca by an effluent from a NSAID-manufacturing plant in Mexico. Ecotoxicology, 25(7), 1288-1304. doi:10.1007/s10646-016-1682-2 |
| YES | Ofoegbu, P. U., Lourenco, J., Mendo, S., Soares, A., & Pestana, J. L. T. (2019). Effects of low concentrations of psychiatric drugs (carbamazepine and fluoxetine) on the freshwater planarian, Schmidtea mediterranea. Chemosphere, 217, 542-549. doi:10.1016/j.chemosphere.2018.10.198 |
| NO | Olvera-Nestor, C. G., Morales-Avila, E., Gomez-Olivan, L. M., Galar-Martinez, M., Garcia-Medina, S., & Neri-Cruz, N. (2016). Biomarkers of Cytotoxic, Genotoxic and Apoptotic Effects in Cyprinus carpio Exposed to Complex Mixture of Contaminants from Hospital Effluents. Bulletin of Environmental Contamination and Toxicology, 96(3), 326-332. doi:10.1007/s00128-015-1721-3 |
| NO | Olvera-Vargas, H., Leroy, S., Rivard, M., Oturan, N., Oturan, M., & Buisson, D. (2016). Microbial biotransformation of furosemide for environmental risk assessment: identification of metabolites and toxicological evaluation. Environmental Science and Pollution Research, 23(22), 22691-22700. doi:10.1007/s11356-016-7398-2 |
| NO | Oropesa, A. L., Floro, A. M., & Palma, P. (2017). Toxic potential of the emerging contaminant nicotine to the aquatic ecosystem. Environmental Science and Pollution Research, 24(20), 16605-16616. doi:10.1007/s11356-017-9084-4 |
| YES | Parente, C. E. T., Sierra, J., & Marti, E. (2018). Ecotoxicity and Biodegradability of Oxytetracycline and Ciprofloxacin on Terrestrial and Aquatic Media. Orbital-the Electronic Journal of Chemistry, 10(4), 262-271. doi:10.17807/orbital.v10i4.1063 |
| NO | Park, J. C., Yoon, D. S., Byeon, E., Seo, J. S., Hwang, U. K., Han, J., & Lee, J. S. (2018). Adverse effects of two pharmaceuticals acetaminophen and oxytetracycline on life cycle parameters, oxidative stress, and defensome system in the marine rotifer Brachionus rotundiformis. Aquatic Toxicology, 204, 70-79. doi:10.1016/j.aquatox.2018.08.018 |
| NO | Perez-Alvarez, I., Islas-Flores, H., Gomez-Olivan, L. M., Barcelo, D., De Alda, M. L., Solsona, S. P., . . . Galar-Martinez, M. (2018). Determination of metals and pharmaceutical compounds released in hospital wastewater from Toluca, Mexico, and evaluation of their toxic impact. Environmental Pollution, 240, 330-341. doi:10.1016/j.envpol.2018.04.116 |
| YES | Pinckney, J. L., Thompson, L., & Hylton, S. (2017). Triclosan alterations of estuarine phytoplankton community structure. Marine Pollution Bulletin, 119(1), 162-168. doi:10.1016/j.marpolbul.2017.03.056 |
| YES | Pino, M. R., Muniz, S., Val, J., & Navarro, E. (2016). Phytotoxicity of 15 common pharmaceuticals on the germination of Lactuca sativa and photosynthesis of Chlamydomonas reinhardtii. Environmental Science and Pollution Research, 23(22), 22530-22541. doi:10.1007/s11356-016-7446-y |
| NO | Prata, J. C., Lavorante, B., Montenegro, M., & Guilhermino, L. (2018). Influence of microplastics on the toxicity of the pharmaceuticals procainamide and doxycycline on the marine microalgae Tetraselmis chuii. Aquatic Toxicology, 197, 143-152. doi:10.1016/j.aquatox.2018.02.015 |
| NO | Ramesh, M., Anitha, S., Poopal, R. K., & Shobana, C. (2018). Evaluation of acute and sublethal effects of chloroquine (C18H26CIN3) on certain enzymological and histopathological biomarker responses of a freshwater fish Cyprinus carpio. Toxicology Reports, 5, 18-27. doi:10.1016/j.toxrep.2017.11.006 |
| NO | Ribeiro, A. R., Sures, B., & Schmidt, T. C. (2018). Ecotoxicity of the two veterinarian antibiotics ceftiofur and cefapirin before and after photo-transformation. Science of the Total Environment, 619, 866-873. doi:10.1016/j.scitotenv.2017.11.109 |
| NO | Ribeiro, W. L. C., Andre, W. P. P., Cavalcante, G. S., de Araujo, J. V., Santos, J. M. L., Macedo, I. T. F., . . . Bevilaqua, C. M. L. (2017). Effects of Spigelia anthelmia decoction on sheep gastrointestinal nematodes. Small Ruminant Research, 153, 146-152. doi:10.1016/j.smallrumres.2017.06.001 |
| NO | Rico, A., Zhao, W. K., Gillissen, F., Lurling, M., & Van den Brink, P. J. (2018). Effects of temperature, genetic variation and species competition on the sensitivity of algae populations to the antibiotic enrofloxacin. Ecotoxicology and Environmental Safety, 148, 228-236. doi:10.1016/j.ecoenv.2017.10.010 |
| NO | Ros, N., Lomba, L., Ribate, M. P., Zuriaga, E., Garcia, C. B., & Giner, B. (2018). Acute lethal and sublethal effects of diltiazem and doxepin for four aquatic environmental bioindicators covering the trophic chain. Aims Environmental Science, 5(4), 229-243. doi:10.3934/environsci.2018.4.229 |
| YES | Rowett, C. J., Hutchinson, T. H., & Comber, S. D. W. (2016). The impact of natural and anthropogenic Dissolved Organic Carbon (DOC), and pH on the toxicity of triclosan to the crustacean Gammarus pulex (L.). Science of the Total Environment, 565, 222-231. doi:10.1016/j.scitotenv.2016.04.170 |
| YES | Russo, C., Lavorgna, M., Cesen, M., Kosjek, T., Heath, E., & Isidori, M. (2018). Evaluation of acute and chronic ecotoxicity of cyclophosphamide, ifosfamide, their metabolites/transformation products and UV treated samples. Environmental Pollution, 233, 356-363. doi:10.1016/j.envpol.2017.10.066 |
| NO | Saari, G. N., Corrales, J., Haddad, S. P., Chambliss, C. K., & Brooks, B. W. (2018). Influence of Diltiazem on Fathead Minnows Across Dissolved Oxygen Gradients. Environmental Toxicology and Chemistry, 37(11), 2835-2850. doi:10.1002/etc.4242 |
| NO | Saleh-E-In, M. M., Sultana, N., Rahim, M. M., Ahsan, M. A., Bhuiyan, M. N. H., Hossain, M. N., . . . Islam, M. R. (2017). Chemical composition and pharmacological significance of Anethum Sowa L. Root. Bmc Complementary and Alternative Medicine, 17. doi:10.1186/s12906-017-1601-y |
| NO | Salesa, B., Ferrando, M. D., Villarroel, M. J., & Sancho, E. (2017). Effect of the lipid regulator Gemfibrozil in the Cladocera Daphnia magna at different temperatures. Journal of Environmental Science and Health Part a-Toxic/Hazardous Substances & Environmental Engineering, 52(3), 228-234. doi:10.1080/10934529.2016.1246937 |
| YES | Santos, N. D., Oliveira, R., Lisboa, C. A., Pinto, J. M. E., Sousa-Moura, D., Camargo, N. S., . . . Domingues, I. (2018). Chronic effects of carbamazepine on zebrafish: Behavioral, reproductive and biochemical endpoints. Ecotoxicology and Environmental Safety, 164, 297-304. doi:10.1016/j.ecoenv.2018.08.015 |
| NO | Santos, P. F. P., Gomes, L., Mazzei, J. L., Fontao, A. P. A., Sampaio, A. L. F., Siani, A. C., & Valente, L. M. M. (2018). POLYPHENOL AND TRITERPENOID CONSTITUENTS OF Eugenia orida DC. (MYRTACEAE) LEAVES AND THEIR ANTIOXIDANT AND CYTOTOXIC POTENTIAL. Quimica Nova, 41(10), 1140-1149. doi:10.21577/0100-4042.20170284 |
| NO | Satyro, S., Saggioro, E. M., Verissimo, F., Buss, D. F., Magalhaes, D. D., & Oliveira, A. (2017). Triclocarban: UV photolysis, wastewater disinfection, and ecotoxicity assessment using molecular biomarkers. Environmental Science and Pollution Research, 24(19), 16077-16085. doi:10.1007/s11356-017-9165-4 |
| NO | Savorelli, F., Manfra, L., Croppo, M., Tornambe, A., Palazzi, D., Canepa, S., . . . Faggio, C. (2017). Fitness Evaluation of Ruditapes philippinarum Exposed to Ni. Biological Trace Element Research, 177(2), 384-393. doi:10.1007/s12011-016-0885-y |
| NO | Schmidt, A. M., Sengupta, N., Saski, C. A., Noorai, R. E., & Baldwin, W. S. (2017). RNA sequencing indicates that atrazine induces multiple detoxification genes in Daphnia magna and this is a potential source of its mixture interactions with other chemicals. Chemosphere, 189, 699-708. doi:10.1016/j.chemosphere.2017.09.107 |
| NO | Schwaickhardt, R. D., Machado, E. L., & Lutterbeck, C. A. (2017). Combined use of VUV and UVC photoreactors for the treatment of hospital laundry wastewaters: Reduction of load parameters, detoxification and life cycle assessment of different configurations. Science of the Total Environment, 590, 233-241. doi:10.1016/j.scitotenv.2017.02.218 |
| NO | Scott, G. I., Porter, D. E., Norman, R. S., Scott, C. H., Uyaguari-Diaz, M. I., Maruya, K. A., . . . Denslow, N. D. (2016). Antibiotics as CECs: An Overview of the Hazards Posed by Antibiotics and Antibiotic Resistance. Frontiers in Marine Science, 3. doi:10.3389/fmars.2016.00024 |
| NO | Shadrick, W. R., Slavish, P. J., Chai, S. C., Waddell, B., Connelly, M., Low, J. A., . . . Potter, P. M. (2018). Exploiting a water network to achieve enthalpy-driven, bromodomain-selective BET inhibitors. Bioorganic & Medicinal Chemistry, 26(1), 25-36. doi:10.1016/j.bmc.2017.10.042 |
| NO | Shao, L., Li, J. Y., Zhang, Y. J., Song, Y. Y., Yu, K. F., He, P. M., & Shen, A. L. (2018). Herbicidal effects of Chinese herbal medicine Coptis chinensis Franch. extract on duckweed (Spirodela polyrhiza (L.) Schleid.). Ecological Engineering, 115, 9-14. doi:10.1016/j.ecoleng.2018.02.002 |
| NO | Sharaibi, O. J., & Afolayan, A. J. (2017). Phytochemical analysis and toxicity evaluation of acetone, aqueous and methanolic leaf extracts of agapanthus praecox willd. International Journal of Pharmaceutical Sciences and Research, 8(12), 5342-5348. doi:10.13040/ijpsr.0975-8232.8(12).5342-48 |
| NO | Sharma, S., Sharma, R. S., Sardesai, M. M., & Mishra, V. (2018). Anticancer potential of leafless mistletoe (viscum angulatum) from western ghats of india. International Journal of Pharmaceutical Sciences and Research, 9(5), 1902-1907. doi:10.13040/ijpsr.0975-8232.9(5).1902-07 |
| YES | Sidhu, H., O'Connor, G., & Kruse, J. (2019). Plant toxicity and accumulation of biosolids-borne ciprofloxacin and azithromycin. Science of the Total Environment, 648, 1219-1226. doi:10.1016/j.scitotenv.2018.08.218 |
| NO | Singh, P., & Nel, A. (2017). A comparison between Daphnia pulex and Hydra vulgaris as possible test organisms for agricultural run-off and acid mine drainage toxicity assessments. Water Sa, 43(2), 323-332. doi:10.4314/wsa.v43i2.15 |
| NO | Skibinski, R., Komsta, L., & Inglot, T. (2016). Characterization of paliperidone photodegradation products by LC-Q-TOF multistage mass spectrometry. Biomedical Chromatography, 30(6), 894-901. doi:10.1002/bmc.3625 |
| NO | Sobrino-Figueroa, A. (2016). Toxic effects of emerging pollutants in juveniles of the freshwater gastropod Physa acuta (Draparnaud, 1805). American Malacological Bulletin, 33(2), 337-342. doi:10.4003/006.033.0211 |
| NO | Soliman, S. M., Albering, J. H., Farooq, M., Wadaan, M. A. M., & El-Faham, A. (2017). Synthesis, structural and biological studies of two new Co(III) complexes with tridentate hydrazone ligand derived from the antihypertensive drug hydralazine. Inorganica Chimica Acta, 466, 16-29. doi:10.1016/j.ica.2017.05.045 |
| NO | Song, C. G., Song, K. G., Wu, X. H., Tu, X., Qi, X. Z., Wang, G. X., & Ling, F. (2018). Antiparasitic efficacy and safety assessment of magnolol against Ichthyophthirius multifiliis in goldfish. Aquaculture, 486, 9-17. doi:10.1016/j.aquaculture.2017.12.002 |
| NO | Sposito, J. C. V., Montagner, C. C., Casado, M., Navarro-Martin, L., Solorzano, J. C. J., Pina, B., & Grisolia, A. B. (2018). Emerging contaminants in Brazilian rivers: Occurrence and effects on gene expression in zebrafish (Danio rerio) embryos. Chemosphere, 209, 696-704. doi:10.1016/j.chemosphere.2018.06.046 |
| NO | Suely, A., Zabed, H., Ahmed, A. B. A., Mohamad, J., Nasiruddin, M., Sahu, J. N., & Ganesan, P. (2016). Toxicological and hematological effect of Terminalia arjuna bark extract on a freshwater catfish, Heteropneustes fossilis. Fish Physiology and Biochemistry, 42(2), 431-444. doi:10.1007/s10695-015-0149-3 |
| NO | Sumitha, S., Vasanthi, S., Shalini, S., Chinni, S. V., Gopinath, S. C. B., Kathiresan, S., . . . Ravichandran, V. (2019). Durio zibethinus rind extract mediated green synthesis of silver nanoparticles: Characterization and biomedical applications. Pharmacognosy Magazine, 15(60), 52-58. doi:10.4103/pm.pm_400_18 |
| NO | Sun, H. Q., Du, Y., Zhang, Z. Y., Jiang, W. J., Guo, Y. M., Lu, X. W., . . . Sun, L. W. (2016). Acute Toxicity and Ecological Risk Assessment of Benzophenone and N,N-Diethyl-3 Methylbenzamide in Personal Care Products. International Journal of Environmental Research and Public Health, 13(9). doi:10.3390/ijerph13090925 |
| NO | Tebby, C., Joachim, S., Van den Brink, P. J., Porcher, J. M., & Beaudouin, R. (2017). Analysis of community-level mesocosm data based on ecologically meaningful dissimilarity measures and data transformation. Environmental Toxicology and Chemistry, 36(6), 1667-1679. doi:10.1002/etc.3701 |
| NO | Telfer, T. J., Liddell, J. R., Duncan, C., White, A. R., & Codd, R. (2017). Adamantyl- and other polycyclic cage-based conjugates of desferrioxamine B (DFOB) for treating iron-mediated toxicity in cell models of Parkinson's disease. Bioorganic & Medicinal Chemistry Letters, 27(8), 1698-1704. doi:10.1016/j.bmcl.2017.03.001 |
| NO | Thomaidi, V. S., Matsoukas, C., & Stasinakis, A. S. (2017). Risk assessment of triclosan released from sewage treatment plants in European rivers using a combination of risk quotient methodology and Monte Carlo simulation. Science of the Total Environment, 603, 487-494. doi:10.1016/j.scitotenv.2017.06.113 |
| NO | Thomaidi, V. S., Stasinakis, A. S., Borova, V. L., & Thomaidis, N. S. (2016). Assessing the risk associated with the presence of emerging organic contaminants in sludge-amended soil: A country-level analysis. Science of the Total Environment, 548, 280-288. doi:10.1016/j.scitotenv.2016.01.043 |
| NO | Tobajas, M., Verdugo, V., Polo, A. M., Rodriguez, J. J., & Mohedano, A. F. (2016). Assessment of toxicity and biodegradability on activated sludge of priority and emerging pollutants. Environmental Technology, 37(6), 713-721. doi:10.1080/09593330.2015.1079264 |
| NO | Toolabi, A., Malakootian, M., Ghaneian, M. T., Esrafili, A., Ehrampoush, M. H., AskarShahi, M., & Tabatabaei, M. (2018). Modeling photocatalytic degradation of diazinon from aqueous solutions and effluent toxicity risk assessment using Escherichia coli LMG 15862. Amb Express, 8. doi:10.1186/s13568-018-0589-0 |
| NO | Torres, T., Cunha, I., Martins, R., & Santos, M. M. (2016). Screening the Toxicity of Selected Personal Care Products Using Embryo Bioassays: 4-MBC, Propylparaben and Triclocarban. International Journal of Molecular Sciences, 17(10). doi:10.3390/ijms17101762 |
| YES | Trombini, C., Hampel, M., & Blasco, J. (2016). Evaluation of acute effects of four pharmaceuticals and their mixtures on the copepod Tisbe battagliai. Chemosphere, 155, 319-328. doi:10.1016/j.chemosphere.2016.04.058 |
| NO | Turkay, O., Barisci, S., Ulusoy, E., Seker, M. G., & Dimoglo, A. (2018). Anodic oxidation of anti-cancer drug Imatinib on different electrodes: Kinetics, transformation by-products and toxicity assessment. Electrochimica Acta, 263, 400-408. doi:10.1016/j.electacta.2018.01.079 |
| NO | Tuvaanjav, S., Shuqin, H., Komata, M., Ma, C. J., Kanamoto, T., Nakashima, H., & Yoshida, T. (2016). Isolation and antiviral activity of water-soluble Cynomorium songaricum Rupr. polysaccharides. Journal of Asian Natural Products Research, 18(2), 159-171. doi:10.1080/10286020.2015.1082547 |
| NO | Vajargah, M. F., Yalsuyi, A. M., & Hedayati, A. (2017). Acute toxicity of povidone-iodine (Betadine) in common carp (Cyprinus carpio L. 1758). Pollution, 3(4), 589-593. doi:10.22059/poll.2017.62775 |
| NO | Varano, V., Fabbri, E., & Pasteris, A. (2017). Assessing the environmental hazard of individual and combined pharmaceuticals: acute and chronic toxicity of fluoxetine and propranolol in the crustacean Daphnia magna. Ecotoxicology, 26(6), 711-728. doi:10.1007/s10646-017-1803-6 |
| YES | Vestel, J., Caldwell, D. J., Constantine, L., D'Aco, V. J., Davidson, T., Dolan, D. G., . . . Wilson, P. (2016). Use of acute and chronic ecotoxicity data in environmental risk assessment of pharmaceuticals. Environmental Toxicology and Chemistry, 35(5), 1201-1212. doi:10.1002/etc.3260 |
| NO | Villa, S., Di Nica, V., Bellamoli, F., Pescatore, T., Ferrario, C., Finizio, A., & Lencioni, V. (2018). Effects of a treated sewage effluent on behavioural traits in Diamesa cinerella and Daphnia magna. Journal of Limnology, 77, 121-130. doi:10.4081/jlimnol.2018.1760 |
| YES | Villa, S., Di Nica, V., Pescatore, T., Bellamoli, F., Miari, F., Finizio, A., & Lencioni, V. (2018). Comparison of the behavioural effects of pharmaceuticals and pesticides on Diamesa zernyi larvae (Chironomidae). Environmental Pollution, 238, 130-139. doi:10.1016/j.envpol.2018.03.029 |
| NO | Wagner, N. D., Simpson, A. J., & Simpson, M. J. (2017). Metabolomic responses to sublethal contaminant exposure in neonate and adult Daphnia magna. Environmental Toxicology and Chemistry, 36(4), 938-946. doi:10.1002/etc.3604 |
| NO | Wagner, N. D., Simpson, A. J., & Simpson, M. J. (2018). Sublethal metabolic responses to contaminant mixture toxicity in Daphnia magna. Environmental Toxicology and Chemistry, 37(9), 2448-2457. doi:10.1002/etc.4208 |
| NO | Wang, M. C., Zhu, P. L., Zhao, S. W., Nie, C. Z. P., Wang, N. F., Du, X. F., & Zhou, Y. B. (2017). Characterization, antioxidant activity and immunomodulatory activity of polysaccharides from the swollen culms of Zizania latifolia. International Journal of Biological Macromolecules, 95, 809-817. doi:10.1016/j.ijbiomac.2016.12.010 |
| NO | Wang, Z., Kang, D. W., Chen, M., Wu, G. C., Feng, D., Zhao, T., . . . Liu, X. Y. (2018). Design, synthesis, and antiviral evaluation of novel hydrazone-substituted thiophene 3,2-d pyrimidine derivatives as potent human immunodeficiency virus-1 inhibitors. Chemical Biology & Drug Design, 92(6), 2009-2021. doi:10.1111/cbdd.13373 |
| YES | Watanabe, H., Tamura, I., Abe, R., Takanobu, H., Nakamura, A., Suzuki, T., . . . Tatarazako, N. (2016). Chronic toxicity of an environmentally relevant mixture of pharmaceuticals to three aquatic organisms (alga, daphnid, and fish). Environmental Toxicology and Chemistry, 35(4), 996-1006. doi:10.1002/etc.3285 |
| YES | Wei, S., Wang, F. H., Chen, Y. J., Lan, T., & Zhang, S. T. (2018). The joint toxicity effect of five antibiotics and dibutyl phthalate to luminescent bacteria (Vibrio fischeri). Environmental Science and Pollution Research, 25(26), 26504-26511. doi:10.1007/s11356-018-2720-9 |
| YES | Wieczerzak, M., Kudlak, B., & Namiesnik, J. (2016). Study of the effect of residues of pharmaceuticals on the environment on the example of bioassay Microtox (R). Monatshefte Fur Chemie, 147(8), 1455-1460. doi:10.1007/s00706-016-1782-y |
| YES | Wu, M. N. N., Wang, X. C. C., & Ma, X. Y. Y. (2016). Phytotoxicity comparison of organic contaminants and heavy metals using Chlorella vulgaris. Desalination and Water Treatment, 57(44), 20809-20816. doi:10.1080/19443994.2015.1110537 |
| NO | Xiong, J. Q., Govindwar, S., Kurade, M. B., Paeng, K. J., Roh, H. S., Khan, M. A., & Jeon, B. H. (2019). Toxicity of sulfamethazine and sulfamethoxazole and their removal by a green microalga, Scenedesmus obliquus. Chemosphere, 218, 551-558. doi:10.1016/j.chemosphere.2018.11.146 |
| YES | Xiong, J. Q., Kurade, M. B., Kim, J. R., Roh, H. S., & Jeon, B. H. (2017). Ciprofloxacin toxicity and its co-metabolic removal by a freshwater microalga Chlamydomonas mexicana. Journal of Hazardous Materials, 323, 212-219. doi:10.1016/j.jhazmat.2016.04.073 |
| YES | Xiong, J. Q., Miracle, M. B., & Jeon, B. H. (2017). Ecotoxicological effects of enrofloxacin and its removal by monoculture of microalgal species and their consortium. Environmental Pollution, 226, 486-493. doi:10.1016/j.envpol.2017.04.044 |
| NO | Yamindago, A., Lee, N., Woo, S., Choi, H., Mun, J. Y., Jang, S. W., . . . Yum, S. (2018). Acute toxic effects of zinc oxide nanoparticles on Hydra magnipapillata. Aquatic Toxicology, 205, 130-139. doi:10.1016/j.aquatox.2018.10.008 |
| YES | Ye, J., Du, Y. P., Wang, L. M., Qian, J. R., Chen, J. J., Wu, Q. W., & Hu, X. J. (2017). Toxin Release of Cyanobacterium Microcystis aeruginosa after Exposure to Typical Tetracycline Antibiotic Contaminants. Toxins, 9(2). doi:10.3390/toxins9020053 |
| NO | Yeo, C. R., Yong, J. J., & Popovich, D. G. (2017). Isolation and characterization of bioactive polyacetylenes Panax ginseng Meyer roots. Journal of Pharmaceutical and Biomedical Analysis, 139, 148-155. doi:10.1016/j.jpba.2017.02.054 |
| YES | Yokota, H., Taguchi, Y., Tanaka, Y., Uchiyama, M., Kondo, M., Tsuruda, Y., . . . Eguchi, S. (2018). Chronic exposure to diclofenac induces delayed mandibular defects in medaka (Oryzias latipes) in a sex-dependent manner. Chemosphere, 210, 139-146. doi:10.1016/j.chemosphere.2018.07.016 |
| NO | Zahra, K., Yadav, S., Tanya, Jyoti, Deeksha, Sandeep, & Deepti. (2016). Assessment of acute toxicity of cypermethrin and its mitigation by green tea extract in fresh water fishes, channa punctatus. Indo American Journal of Pharmaceutical Sciences, 3(4), 374-378. |
| NO | Zahra, S. S., Ahmed, M., Qasim, M., Gul, B., Zia, M., Mirza, B., & Ihsan-ul, H. (2017). Polarity based characterization of biologically active extracts of Ajuga bracteosa Wall. ex Benth. and RP-HPLC analysis. Bmc Complementary and Alternative Medicine, 17. doi:10.1186/s12906-017-1951-5 |
| NO | Zaleska-Radziwill, M., Affek, K., & Doskocz, N. (2017). Ecotoxicological risk assessment of chosen pharmaceuticals detected in surface waters. Journal of Environmental Science and Health Part a-Toxic/Hazardous Substances & Environmental Engineering, 52(13), 1233-1239. doi:10.1080/10934529.2017.1356199 |
| YES | Zanuri, N. B. M., Bentley, M. G., & Caldwell, G. S. (2017). Assessing the impact of diclofenac, ibuprofen and sildenafil citrate (Viagra (R)) on the fertilisation biology of broadcast spawning marine. Marine Environmental Research, 127, 126-136. doi:10.1016/j.marenvres.2017.04.005 |
| NO | Zhang, H., Tian, Y., Kang, D. W., Huo, Z. P., Zhou, Z. X., Liu, H. Q., . . . Liu, X. Y. (2017). Discovery of uracil-bearing DAPYs derivatives as novel HIV-1 NNRTIs via crystallographic overlay-based molecular hybridization. European Journal of Medicinal Chemistry, 130, 209-222. doi:10.1016/j.ejmech.2017.02.047 |
| YES | Zhang, L. L., Niu, J. F., & Wang, Y. J. (2016). Full life-cycle toxicity assessment on triclosan using rotifer Brachionus calyciflorus. Ecotoxicology and Environmental Safety, 127, 30-35. doi:10.1016/j.ecoenv.2015.12.043 |
| NO | Zhang, Y. N., Wang, X. D., Yin, X. H., Shi, M. R., Dahlgren, R. A., & Wang, H. L. (2016). Toxicity Assessment of Combined Fluoroquinolone and Tetracycline Exposure in Zebrafish (Danio rerio). Environmental Toxicology, 31(6), 736-750. doi:10.1002/tox.22087 |
| YES | Zhou, Z., Yang, J., & Chan, K. M. (2017). Toxic effects of triclosan on a zebrafish (Danio rerio) liver cell line, ZFL. Aquatic Toxicology, 191, 175-188. doi:10.1016/j.aquatox.2017.08.009 |
| YES | Zhu, L. Y., Santiago-Schubel, B., Xiao, H. X., Hollert, H., & Kueppers, S. (2016). Electrochemical oxidation of fluoroquinolone antibiotics: Mechanism, residual antibacterial activity and toxicity change. Water Research, 102, 52-62. doi:10.1016/j.watres.2016.06.005 |
| YES | Zivna, D., Plhalova, L., Chromcova, L., Blahova, J., Prokes, M., Skoric, M., . . . Svobodova, Z. (2016). The effects of ciprofloxacin on early life stages of common carp (Cyprinus carpio). Environmental Toxicology and Chemistry, 35(7), 1733-1740. doi:10.1002/etc.3317 |
| NO | Zortea, T., dos Reis, T. R., Serafini, S., de Sousa, J. P., da Silva, A. S., & Baretta, D. (2018). Ecotoxicological effect of fipronil and its metabolites on Folsomia candida in tropical soils. Environmental Toxicology and Pharmacology, 62, 203-209. doi:10.1016/j.etap.2018.07.011 |
| NO | Zuriaga, E., Lomba, L., German, B., Lanuza, P. M., Aldea, L., Ribate, M. P., . . . Giner, B. (2019). Ecotoxicity in Aliivibrio fischeri of Ibuprofen, Omeprazole and their Mixtures. Chemistry and Ecology, 35(2), 102-114. doi:10.1080/02757540.2018.1540608 |

**TABLE S12:** CRED scores evaluating the reliability and relevance of critical literature articles for their inclusion in the derivation of safe concentration in this study.

| *Articles* | *Reliability* | *Relevance* | *Sufficient quality? ^a^* |
| --- | --- | --- | --- |
| Aderemi et al. (2018) | R1 | C1 | Yes |
| Ando et al. (2007) | R4 | C2 | No |
| Bayer et al. (2014) | R4 | C4 | No |
| Chen et al. (2019) | R1 | C2 | Yes |
| De Liguoro et al. (2009) | R2 | C2 | Yes |
| Di Poi et al. (2018)di Poi *et al.* (2018) | R1 | C2 | Yes |
| Dordio et al. (2011) | R3 | C2 | No |
| Eguchi et al. (2004) | R4 | C1 | No |
| Fabbri et al. (2014) | R3 | C4 | No |
| Godoy et al. (2018) | R1 | C1 | Yes |
| González-Pleiter et al. (2013) | R2 | C2 | Yes |
| Han et al. (2006) | R3 | C1 | No |
| He et al. (2013) | R2 | C2 | Yes |
| Jarvis et al. (2014) | R2 | C3 | No |
| Ji et al. (2012) | R2 | C1 | Yes |
| Jungmann et al. (2017) | R2 | C2 | Yes |
| Li et al. (2010) | R2 | C2 | Yes |
| Majewska et al. (2018) | R2 | C1 | Yes |
| Martins et al. (2012) | R2 | C2 | Yes |
| Ofoegbu et al. (2019) | R3 | C3 | No |
| Russo et al. (2018) | R2 | C1 | Yes |
| Yang et al. (2008) | R3 | C1 | No |
| Yokota et al. (2018) | R1 | C1 | Yes |
| Monika et al. (2011) | R4 | C1 | No |
| Zhu et al. (2014) | R2 | C1 | Yes |
| Zounková et al. (2007) | R4 | C2 | No |
| ^a^ Studies deemed of sufficient quality had to be assign reliability scores of R1 or R2, and relevance scores of C1 or C2. | | | |

*S2.1 Literature search string*

The titles, abstracts, and keywords were screened using the following search string “(LC50* OR EC50* OR EC10* OR NOEC* OR "effect concentration") AND (aquatic* OR *water*) AND (*toxic*) AND (pharmaceutic* OR medicine* OR drug* OR ((amantadine OR *amant*) OR (carbamazepine OR carbamaz*) OR (ciprofloxacin OR ciproflox*) OR (cyclophosphamide OR c*clo*os*amid*) OR (diclofenac OR diclofenac*) OR (doxycycline OR dox*c*clin*) OR (erythromycin OR er*throm*cin*) OR (ethinylestradiol OR *ethinyl*estradiol) OR (iopamidol OR io*ami* OR "contrast agent") OR (metformin OR metformi* OR dimethylbiguanid* OR dimethylimidodicarbonimidic) OR (metoprolol OR "1-(Isopropylamino)-3-[4-(2-methoxyethyl)phenoxy]-2-propanol") OR (oxazepam OR "7-Chloro-3-hydroxy-5-phenyl-1,3-dihydro-2H-1,4-benzodiazepin-2-one") OR (phenazone OR phenazon* OR antipyrine OR "1,5-Dimethyl-2-phenyl-1,2-dihydro-3H-pyrazol-3-one") OR (sul*amethazine OR sul*adimidin* OR sul*adimethylpyrimidine) OR (valsartan))) NOT QSAR”. At the time of the search, additional compounds besides the eight pharmaceuticals of interest in this study were included, retrieving a total of 233 publications. All these publications were screened in detail but only the ones containing information on the eight pharmaceuticals of interest in this study were used.

*S2.2. Species names*

Harmonized according to most recent taxonomic nomenclature and corrected for misspellings.

*S2.3. Exposure type*

“Chronic” or “acute” classification was primarily assigned according to the authors. If not explicitly mentioned, a decision was made according to the corresponding original methods article referenced (if readily available), or (inter)national chemical testing guidelines (e.g. OECD Test No. 201). Alternatively, the life span of the organism and the exposure duration was considered. In this regard, a 10% lifespan coverage threshold was applied as to decide whether to classify an exposure as chronic or acute(Suter II 2007). For example, *Danio rerio* lives on average 1 year in the wild; bioassays with exposure times higher than 10% of 365 days where tagged “chronic”. Similarly, this threshold was applied in early development stage data under the assumption that exposure during this critical period can potentially exert long-term effects further in the lifecycle. If no exposure time, guideline or protocol were provided, the values were conservatively classified as “acute”.

*S2.4. Effect code*

If effects were not reported or unspecified, these were coded as “UND” (undetermined). Population effects reported as more than one effect like “Survival, reproduction and growth rate” were coded as “POP” (population). In the case of multiple effects in which one or more effects do not necessarily dictate the sustainability of a population, such as “Length, reproduction and survival”, were attributed the code “MUL” (multiple).

*S2.5. Endpoints*

When authors did not explicitly use LOEC or NOEC terminology, the publications’ graphs were inspected to assign the corresponding concentration values according to the results of the statistical tests. In studies where single concentrations were tested, if effects were determined significant, that concentration was classified as “LOEC”. If not significant, a “<” was assigned. Highest concentrations tested showing no effects tagged by the authors as “NOEC” and assigned with “>” were recorded. If not explicitly classified by the authors, these values were coercively assigned “>” to distinguish from studies where both NOEC and LOEC were derived empirically.

*S2.6. Exposure duration*

If several exposure times were given (e.g. interval, 176-301 days) associated with only one effect value, the highest time point is used (e.g. sampled at 8-60 days, only 60 days is accounted for).

*S2.7. Concentration units*

Given the intent of this assessment, only aquatic exposure measured in weight of test substance per volume (e.g. mg/L) were included. All concentrations were converted to μg/L. Unit conversion from molar to μg/L was done using the molecular weight (MW) provided by authors, chemical manufacture company, PubChem (https://pubchem.ncbi.nlm.nih.gov/) or other relevant source. The CAS numbers were used to extract MW. If CAS was not disclosed then the substance name and the corresponding best match result was used.

*S2.8. Substance aggregation*

Different forms or variations of a parent substance were aggregated (Table S13) to circumvent the scarcity of substance-specific effect data and pool compounds with analogous biological activity (e.g. metoprolol tartrate and metoprolol succinate) or metabolically related (e.g. carbamazepine and carbamazepine metabolite trans-10,11-dihydroxy-10,11-dihydrocarbazepine). Moreover, this aggregation prevents overly stringent data exclusion due to incomplete identification of the substance (e.g. missing CAS registry number).

**TABLE S13:** Grouping of pharmaceuticals.

| *Group* | *Compounds* |
| --- | --- |
| Amantadine | amantadine |
| Carbamazepine | carbamazepine  carbamazepine 10,11-epoxide  trans-10,11-dihydroxy-10,11-dihydrocarbazepine |
| Ciprofloxacin | ciprofloxacin  ciprofloxacin HCl |
| Cyclophosphamide | cyclophosphamide  carboxycyclophosphamide  keto-cyclophosphamide  N-dechloroethyl-cyclophosphamide |
| Diclofenac | diclofenac  diclofenac Na |
| Doxycycline | doxycycline |
| Erythromycin | erythromycin  erythromycin phosphate |
| Ethinylestradiol | ethinylestradiol  17α-ethinylestradiol |
| Iopamidol | iopamidol |
| Metformin | metformin  metformin HCl |
| Metoprolol | metoprolol  metoprolol tartrate  metoprolol succinate |
| Oxazepam | oxazepam |
| Phenazone | phenazone |
| Sulfamethazine | sulfamethazine  sulfadimidine |
| Valsartan | valsartan |

*S2.9. Endpoint Aggregation*

The aggregation of endpoints was done following established guidelines (ECHA, 2008) and according to their closest or equivalent toxicological effect response (Table S14). Aggregated endpoints are in the present study referred simply as ‘endpoints’.

**TABLE S14:** Grouping of available endpoints in the database into aggregated chronic NOEC, chronic EC_50_, acute NOEC and acute EC_50_ endpoints. MATC was reverse calculated to obtain NOEC.

| *Chronic exposure* | |  | *Acute exposure* | |
| --- | --- | --- | --- | --- |
| *NOEC* | *EC_50_* |  | *NOEC* | *EC_50_* |
| EC_10_ | EC_50_ |  | EC_5_ | L(E)C_50_ |
| EC_5_ | ET_50_ |  | EC_10_ | EC_20_ |
| IC_10_ | IC_50_ |  | LC_10_ | EC_25_ |
| IC_5_ | LC_50_ |  | MATC | EC_50_ |
| LC_01_ |  |  | NOEC | IC_50_ |
| LC_10_ |  |  | NOEL | LC_50_ |
| MATC |  |  |  | MTC |
| NOAEC |  |  |  |  |
| NOEC |  |  |  |  |
| NOEL |  |  |  |  |

*S2.10. Predicted no effect concentration*

**TABLE S15:** Chronic ecotoxicological effects on freshwater species. To derive predicted no effect concentrations (PNEC) for each substance a distinct assessment factor (AF) was applied to the most sensitive species and effect depending on the data available.

| *Substance* | *Taxa* | *Species* | *Effect* | *Concentration (µg/L)* | *AF* | *PNEC (µg/L)* |
| --- | --- | --- | --- | --- | --- | --- |
| Carbamazepine | insecta | *Stenonema sp.* | BEH | 0.2 | 10 | 0.02 |
|  | crustacea | *Daphnia similis* | REP | 0.3 |  |  |
|  | algae | *Chaetophora sp.* | POP | 2 |  |  |
|  | crustacea | *Daphnia pulex* | REP | 100 |  |  |
|  | fish | *Pimephales promelas* | BEH | 100 |  |  |
|  | insecta | *Chironomus riparius* | DEV | 164 |  |  |
|  | fish | *Oncorhynchus mykiss* | GRO | 180 |  |  |
|  | crustacea | *Ceriodaphnia dubia* | REP | 199 |  |  |
|  | rotifera | *Brachionus calyciflorus* | REP/MOR | 377 |  |  |
|  | crustacea | *Daphnia magna* | REP/GRO | 400 |  |  |
|  | crustacea | *Hyalella azteca* | MOR | 600 |  |  |
|  | algae | *Chlorella pyrenoidosa* | POP | 1000 |  |  |
|  | algae | *Scenedesmus acutus* | POP | 1000 |  |  |
|  | algae | *Raphidocelis subcapitata* | POP | 2046 |  |  |
|  | insecta | *Chironomus tentans* | GRO | 2600 |  |  |
|  | fish | *Oryzias latipes* | BEH | 6150 |  |  |
|  | algae | *Cyclotella meneghiniana* | POP | 10000 |  |  |
|  | algae | *Chlorella vulgaris* | POP | 11800 |  |  |
|  | fish | *Danio rerio* | REP | 12500 |  |  |
| Ciprofloxacin | fish | *Lebistes reticulatus* | GRO | 780 | 10 | 78 |
|  | fish | *Poecilia reticulata* | GRO | 780 |  |  |
|  | algae | *Raphidocelis subcapitata* | POP | 3006 |  |  |
|  | crustacea | *Daphnia magna* | REP | 3217 |  |  |
| Cyclophosphamide | crustacea | *Ceriodaphnia dubia* | POP | 1250 | 10 | 125 |
|  | rotifera | *Brachionus calyciflorus* | POP | 3394 |  |  |
|  | algae | *Raphidocelis subcapitata* | POP | 12500 |  |  |
|  | fish | *Danio rerio* | MOR | 13743785 |  |  |
| Diclofenac | mollusca | *Dreissena ploymorpha* | MOR | 0.5 | 50 | 0.01 |
|  | fish | *Oryzias latipes* | DEV | 7.29 |  |  |
|  | fish | *Danio rerio* | GRO | 10 |  |  |
|  | fish | *Oncorhynchus mykiss* | REP/DEV/MOR | 1084 |  |  |
|  | algae | *Raphidocelis subcapitata* | GRO | 25000 |  |  |
|  | algae | *Chlamydomonas reinhardtii* | POP | 32700 |  |  |
| Erythromycin | cyanobacteria | *Anabaena sp.* | POP | 5 | 10 | 0.5 |
|  | algae | *Raphidocelis subcapitata* | POP | 23 |  |  |
|  | crustacea | *Daphnia magna* | GRO | 11100 |  |  |
|  | crustacea | *Moina macrocopa* | MOR/REP | 50000 |  |  |
|  | fish | *Oryzias latipes* | MOR | 100000 |  |  |
| 17α-Ethinylestradiol | fish | *Gobiocypris rarus* | REP | 0.00018 | 50 | 3.6x10^-6^ |
|  | fish | *Danio rerio* | DEV | 0.00069 |  |  |
|  | fish | *Rutilus rutilus* | GRO | 0.00071 |  |  |
|  | fish | *Syngnathus scovelli* | DEV | 0.001 |  |  |
|  | fish | *Salmo trutta* | GRO | 0.00208 |  |  |
|  | fish | *Gasterosteus aculeatus* | DEV | 0.00418 |  |  |
|  | amphibia | *Lithobates septentrionalis* | GRO/DEV | 0.005 |  |  |
|  | amphibia | *Lithobates clamitans* | REP | 0.0058 |  |  |
|  | fish | *Salvelinus namaycush* | GRO | 0.0063 |  |  |
|  | fish | *Pimephales promelas* | GRO/REP | 0.008 |  |  |
|  | fish | *Oryzias latipes* | REP | 0.00669 |  |  |
|  | mollusca | *Bithynia tentaculata* | GRO | 0.009 |  |  |
|  | mollusca | *Radix balthica* | GRO | 0.009 |  |  |
|  | fish | *Cyprinodon variegatus* | REP | 0.009 |  |  |
|  | fish | *Alburnus tarichi* | REP | 0.01 |  |  |
|  | amphibia | *Xenopus tropicalis* | DEV | 0.0175 |  |  |
|  | fish | *Etheostoma caeruleum* | DEV | 0.02 |  |  |
|  | mollusca | *Potamopyrgus antipodarum* | REP | 0.025 |  |  |
|  | fish | *Syngnathus abaster* | MOR | 0.02655 |  |  |
|  | fish | *Poecilia reticulata* | DEV/POP | 0.05 |  |  |
|  | mollusca | *Lymnaea stagnalis* | DEV | 0.05 |  |  |
|  | fish | *Oncorhynchus mykiss* | REP | 0.05965 |  |  |
|  | fish | *Fundulus heteroclitus* | MOR | 0.1 |  |  |
|  | crustacea | *Gammarus pulex* | POP | 0.1 |  |  |
|  | mollusca | *Haitia pomilia* | POP | 0.1 |  |  |
|  | fish | *Tautogolabrus adspersus* | MOR | 0.1 |  |  |
|  | mollusca | *Marisa cornuarietis* | REP | 0.5 |  |  |
|  | crustacea | *Daphnia magna* | REP | 14 |  |  |
|  | crustacea | *Sida crystallina* | DEV | 32 |  |  |
|  | crustacea | *Acartia tonsa* | DEV | 46 |  |  |
|  | crustacea | *Hyalella azteca* | GRO/MOR | 70 |  |  |
|  | insecta | *Chironomus tentans* | POP | 88.32 |  |  |
|  | crustacea | *Ceriodaphnia reticulata* | MOR | 200 |  |  |
|  | crustacea | *Ceriodaphnia dubia* | REP | 500 |  |  |
| Metformin | crustacea | *Daphnia similis* | REP | 4400 | 10 | 440 |
|  | crustacea | *Ceriodaphnia dubia* | REP | 7900 |  |  |
|  | fish | *Pimephales promelas* | DEV | 10000 |  |  |
|  | fish | *Brachydanio rerio* | DEV | 11713 |  |  |
|  | crustacea | *Daphnia magna* | REP/MOR | 26593.859 |  |  |
|  | algae | *Raphidocelis subcapitata* | POP | 99749 |  |  |
|  | cnidarian | *Hydra attenuata* | REP | 701800 |  |  |
| Metoprolol | crustacea | *Daphnia magna* | REP | 3100 | 10 | 310 |
|  | algae | *Raphidocelis subcapitata* | POP | 6786 |  |  |
|  | crustacea | *Gammarus fossarum* | REP | 15000 |  |  |
|  | protozoa | *Tetrahymena pyriformis* | GRO | 21800 |  |  |
|  | fish | *Danio rerio* | GRO | 24000 |  |  |

**TABLE S16:** Predicted no effect concentration estimations from literature and this study. Bold numbers indicate values uniquely calculated in this study.

| *Substance* | *PNEC (ug/L)* | *References* |
| --- | --- | --- |
| 17α-Ethinylestradiol | 1.6x10^-2^, 1x10^-4^, 3.7x10^-5^, 3.5x10^-5^, 3.1x10^-5^, 2x10^-5^, **3.6x10^-6^**, 3x10^-8^ | 1-9, 22 |
| Carbamazepine | 170, 130, 17, 10, 8, 2.6, 2.5, 2, 0.5, 0.4, 0.05, **0.02** | 1, 3, 5, 8-20 |
| Ciprofloxacin | **78**, 0.5, 0.45, 0.089, 0.005 | 3, 5, 6, 9, 15, 21, 22 |
| Cyclophosphamide | 1120, 980, 560, **125** | 9, 13, 22 |
| Diclofenac | 50, 32, 31, 20, 10, 0.45, 0.1, 0.05, 0.02, **0.01** | 2, 3, 5, 6, 7, 8, 9, 13, 15, 18, 20, 22, 24, 23 |
| Erythromycin | 2, 0.5, 0.3, 0.2 | 3, 5, 6, 8, 9, 21 |
| Metformin | 1030, 1000, 780, 640, **440**, 156, 100, 20, 13.45, 10, 4.2 | 3, 5, 7, 8, 9, 12, 14, 16, 17, 25-27 |
| Metoprolol | **310**, 75, 64, 62, 58.3, 31, 8.6, 7.3, 3.2 | 3, 5, 8, 9, 12, 16, 17, 22, 28-30 |
| 1. van Vlaardingen et al. (2007). 2 van der Aa et al. (2011). 3. Oekotoxzentrum (2016a). 4. European Union (2011b). 5. NORMAN-network (2019). 6. Loos et al. (2018). 7. Vestel et al. (2016). 8. Agerstrand and Rudén (2010). 9. Perazzolo et al. (2010). 10. Triebskorn et al. (2007). 11. Heye et al. (2019). 12. Lif (2019). 13. Boxall et al. (2014). 14. Comber et al. (2018). 15. Frédéric and Yves (2014). 16. Moermond (2014). 17. Moermond and Smit (2016). 18. Ferrari et al. (2004). 19. Wenzel and Shemotyuk (2014). 20. Gheorghe et al. (2016). 21. AMR Industry Alliance (2018). 22. Grung et al. (2008). 23. Hoeger et al. (2005). 24. European Union (2011a). 25. Caldwell et al. (2019). 26. Oekotoxzentrum (2016b). 27. AstraZeneca (2017a). 28. Oekotoxzentrum (2016c). 29. AstraZeneca (2017b). 30. Murray-Smith et al. (2012). | | |

**S3. SUPPLEMENTARY**

*S3.1.Emission estimation*

Five APIs (carbamazepine, ciprofloxacin, diclofenac, metformin and metoprolol) had a quantification frequency above 90 % in STP influent and were included in the model evaluation exercise. Figure 2 shows that the majority of the predicted influent loads (> 85 %) agree within a factor of 3 with loads derived from measured concentrations. Except for two outliers in the Netherlands, all data points were within a factor of 10 indicating an acceptable overall model performance (Figure S1). Country-specific evaluation reveals differences; influent loads show a small overestimation in Germany and the Netherlands (SSPB_GER_ = 17 %, SSPB_NL_ = 6 %). Erythromycin showed a quantification frequency of less than 50 % in both, German and Dutch STPs. Even though, when erythromycin concentrations below the LoQ were replaced by the LoQ value, i.e. the highest possible quantifiable concentration, influent loads for erythromycin in German STPs are highly overestimated by the model (SSPB = 296 %). Since all other processes (excretion patterns, in-sewer processes) were assumed equal in German and Dutch Vecht regions, erroneous German consumption volumes were most likely responsible for this bias. To bring the overestimation to an acceptable level German erythromycin consumption was adjusted by a factor of 0.5 (SSPB = 99 %) as to account for unknown influencing factors.

In the next step, predicted effluent loads were compared to data (Figure 3 and Figure S2). After STP removal, four APIs (carbamazepine, diclofenac, metformin and metoprolol) had a quantification frequency above 90 %. Overall, predictions of STP effluent loads agreed well with empirical data, showing good accuracy (ξ_effluent_ = 64 %) and small underestimation (SSPB_effluent_ = -22 %). Ciprofloxacin loads were very largely overestimated (SSPB = 288 %) even when measured concentrations below the LoQ were replaced with the LoQ (Supplemental Data, S3). Adjusting ciprofloxacin emissions by a factor of 0.5 lead to an acceptable bias (SSPB = -94 %).


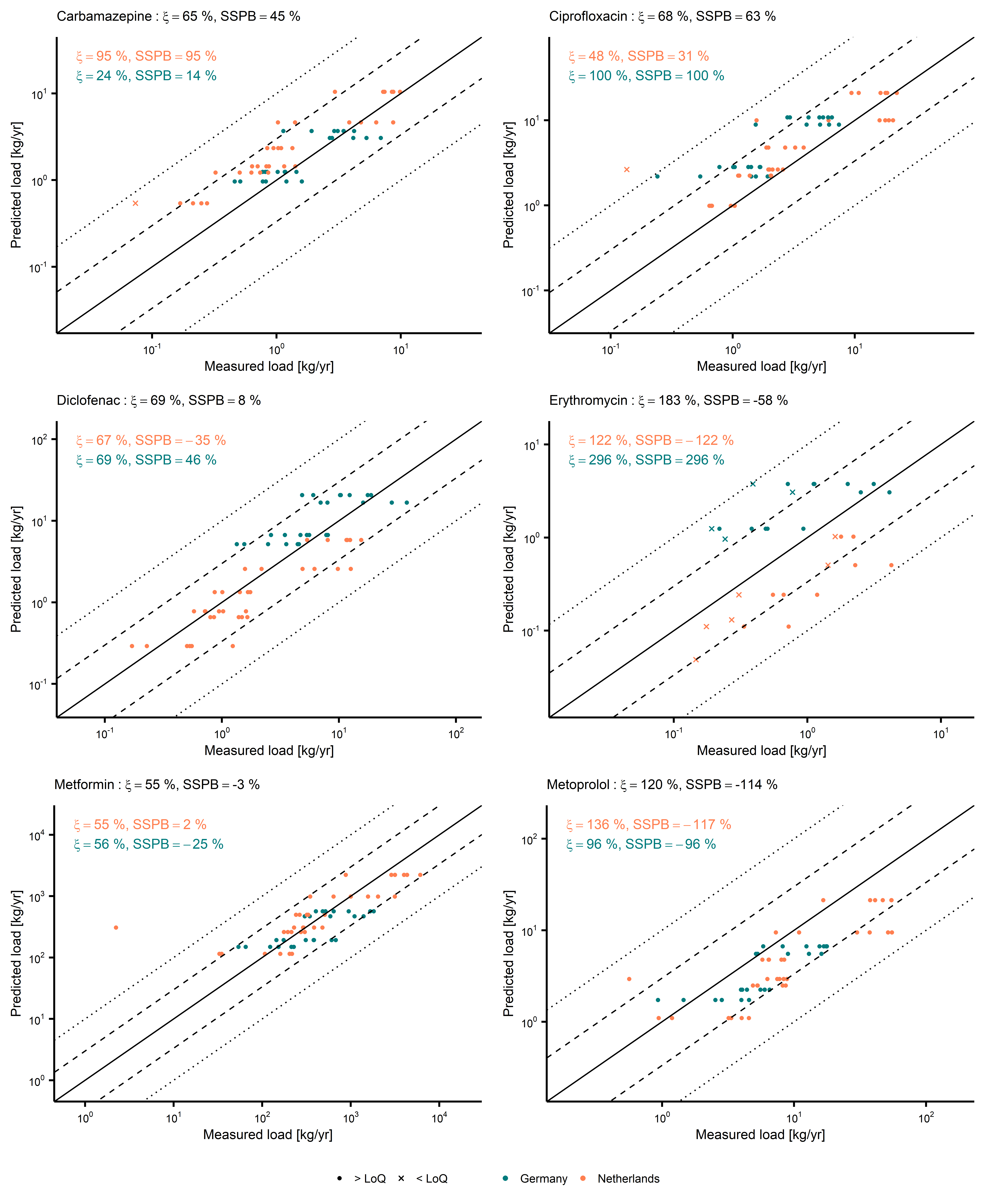


**FIGURE S1:** Predicted and measured STP influent loads of APIs with a detection frequency above 25 %. Dashed lines indicate the 1:3 and 3:1 ratios, dotted lines indicate the 1:10 and 10:1 ratios. All APIs were measured 25 times in German and 34 times in Dutch STPs. Concentrations below the LoQ are processed as LoQ. Actual concentrations are therefore lower and measures (ξ, SSPB) should be taken with care for substances with many concentrations below the LoQ, i.e. erythromycin.


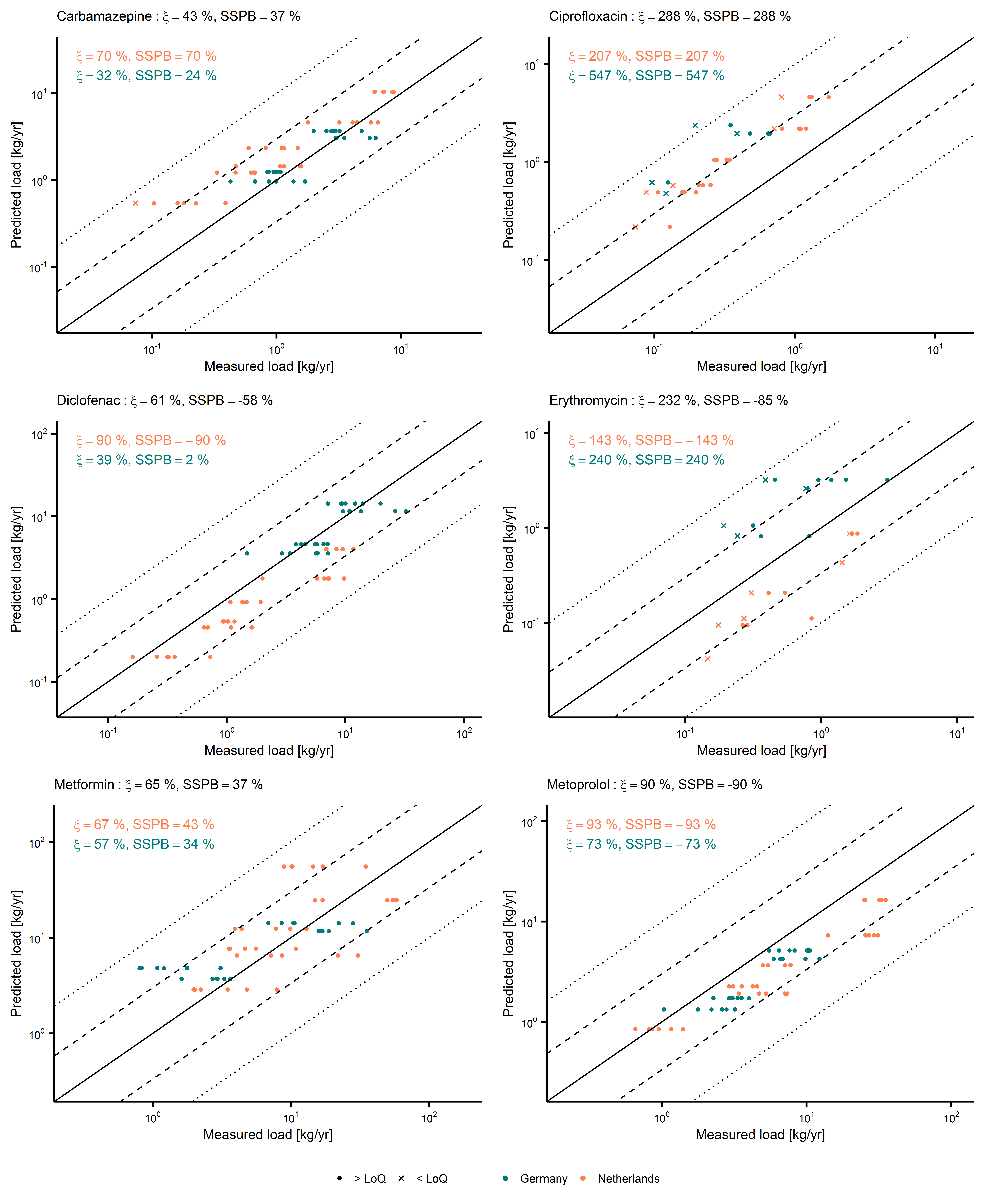
**FIGURE S2:** Predicted and measured STP effluent loads of APIs with a detection frequency above 25 %. Dashed lines indicate the 1:3 and 3:1 ratios, dotted lines indicate the 1:10 and 10:1 ratios. All APIs were measured 25 times in German and 33 times in Dutch STPs. Concentrations below the LoQ are processed as LoQ. Actual concentrations are therefore lower and measures (ξ, SSPB) should be taken with care for substances with many concentrations below the LoQ, i.e. ciprofloxacin and erythromycin.

**
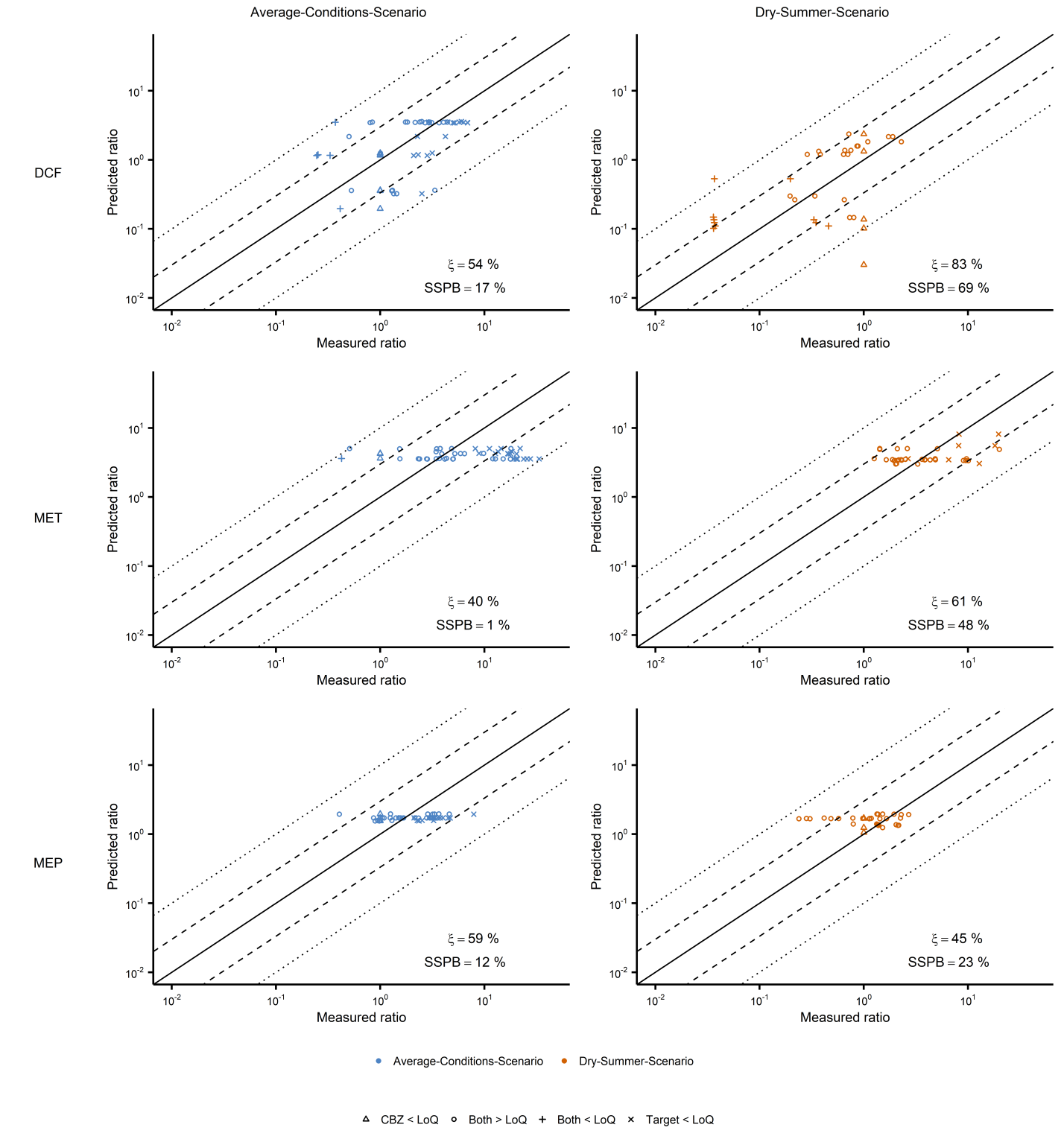
**

**FIGURE S3:** Predicted and measured benchmark ratios of diclofenac, metformin and metoprolol at monitoring sites in the whole Vecht catchment. Dashed lines indicate the 1:3 and 3:1 ratios, dotted lines indicate the 1:10 and 10:1 ratios. Measures were calculated including predicted-measured pairs where both, the target compound and carbamazepine concentrations, were above the LoQ.


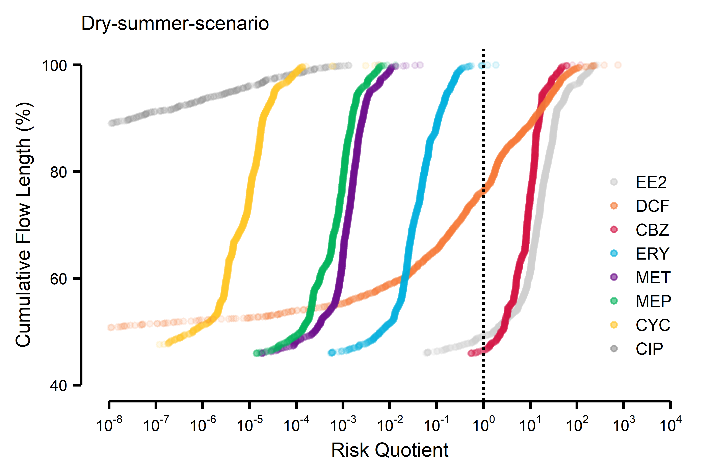

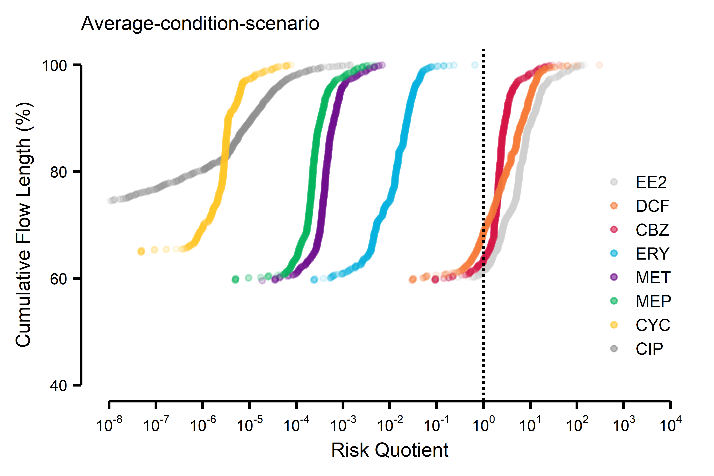


**FIGURE S4:** Percentage of the Vecht catchment flow length at risk of environmental pharmaceutical pollution. Vertical black dashed line indicates the safe threshold RQ = 1, i.e. predicted environmental concentrations equal to the predicted no chronic effect concentration. Risk quotients below 10^-8^ are not depicted in this figure. The Y-axis minimum value was set to 40% since more than 40% of the flow length have RQ = 0. Each point depicts a water stream segment of ≤ 2 km. CBZ, carbamazepine; CIP, ciprofloxacin; CYL, cyclophosphamide; DFC, diclofenac; ERY, erythromycin; EE2, 17α-ethinylestradiol; MET, metformin; MEP, metoprolol.

**TABLE S17:** Water volume percentage and flow length percentage of the Vecht River catchment vulnerable to different ranges of active pharmaceutical ingredients’ (API) risk quotients (RQ). CBZ, carbamazepine; CIP, ciprofloxacin; CYL, cyclophosphamide; DFC, diclofenac; ERY, erythromycin; EE2, 17α-ethinylestradiol; MET, metformin; MEP, metoprolol.

|  |  | *Average-condition-scenario* | | | | |  | *Dry-summer-scenario* | | | | |
| --- | --- | --- | --- | --- | --- | --- | --- | --- | --- | --- | --- | --- |
|  | *API* | *[0, 0]* | *(0, 0.1]* | *(0.1, 1]* | *(1, 10]* | *(10, +∞)* |  | *[0, 0]* | *(0, 0.1]* | *(0.1, 1]* | *(1, 10]* | *(10, +∞)* |
| Water volume (%) | EE2 | 9 |  |  | 65 | 25 |  | 2 |  | 1 | 9 | 87 |
|  | CBZ | 9 |  | 1 | 89 |  |  | 2 |  |  | 66 | 32 |
|  | CIP | 9 | 91 |  |  |  |  | 3 | 97 |  |  |  |
|  | CYC | 12 | 88 |  |  |  |  | 2 | 98 |  |  |  |
|  | DCF | 9 |  | 23 | 63 | 4 |  | 2 | 37 | 34 | 23 | 3 |
|  | ERY | 9 | 91 |  |  |  |  | 2 | 91 | 7 |  |  |
|  | MET | 9 | 91 |  |  |  |  | 2 | 98 |  |  |  |
|  | MEP | 9 | 91 |  |  |  |  | 2 | 98 |  |  |  |
| Flow length (%) | EE2 | 59 |  | 1 | 27 | 11 |  | 46 |  | 3 | 12 | 39 |
|  | CBZ | 59 |  | 3 | 35 | 2 |  | 46 |  | 1 | 29 | 24 |
|  | CIP | 59 | 40 |  |  |  |  | 48 | 52 |  |  |  |
|  | CYC | 65 | 35 |  |  |  |  | 48 | 52 |  |  |  |
|  | DCF | 59 | 1 | 8 | 26 | 6 |  | 46 | 19 | 11 | 12 | 11 |
|  | ERY | 59 | 40 |  |  |  |  | 46 | 43 | 11 |  |  |
|  | MET | 59 | 40 |  |  |  |  | 46 | 54 |  |  |  |
|  | MEP | 59 | 40 |  |  |  |  | 46 | 54 |  |  |  |
|  | | | | | | | | | | | | |


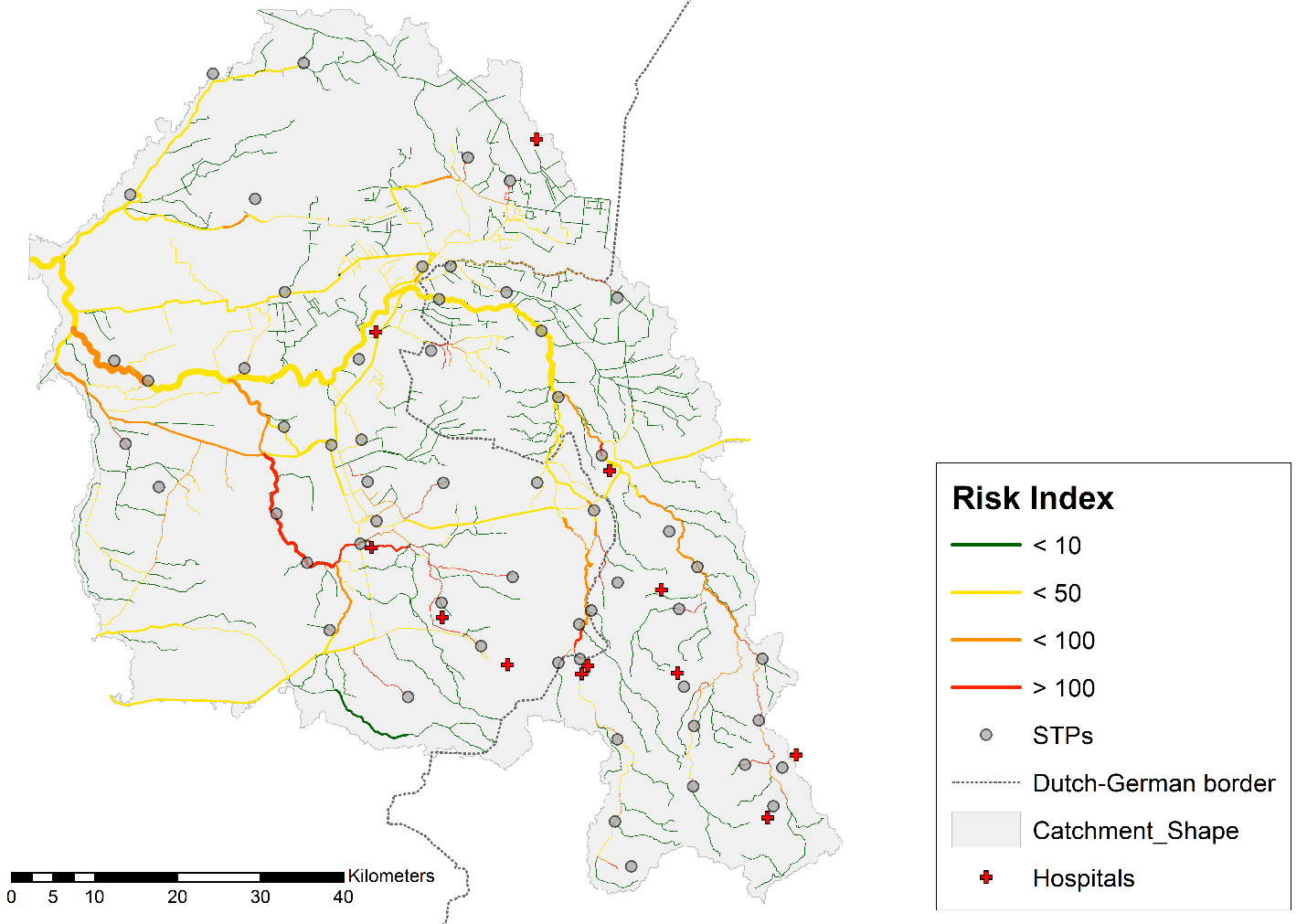


**FIGURE S5:** Risk index map of the Vecht River catchment during a typical dry-summer-scenario. Dashed line demarks the German-Dutch border.


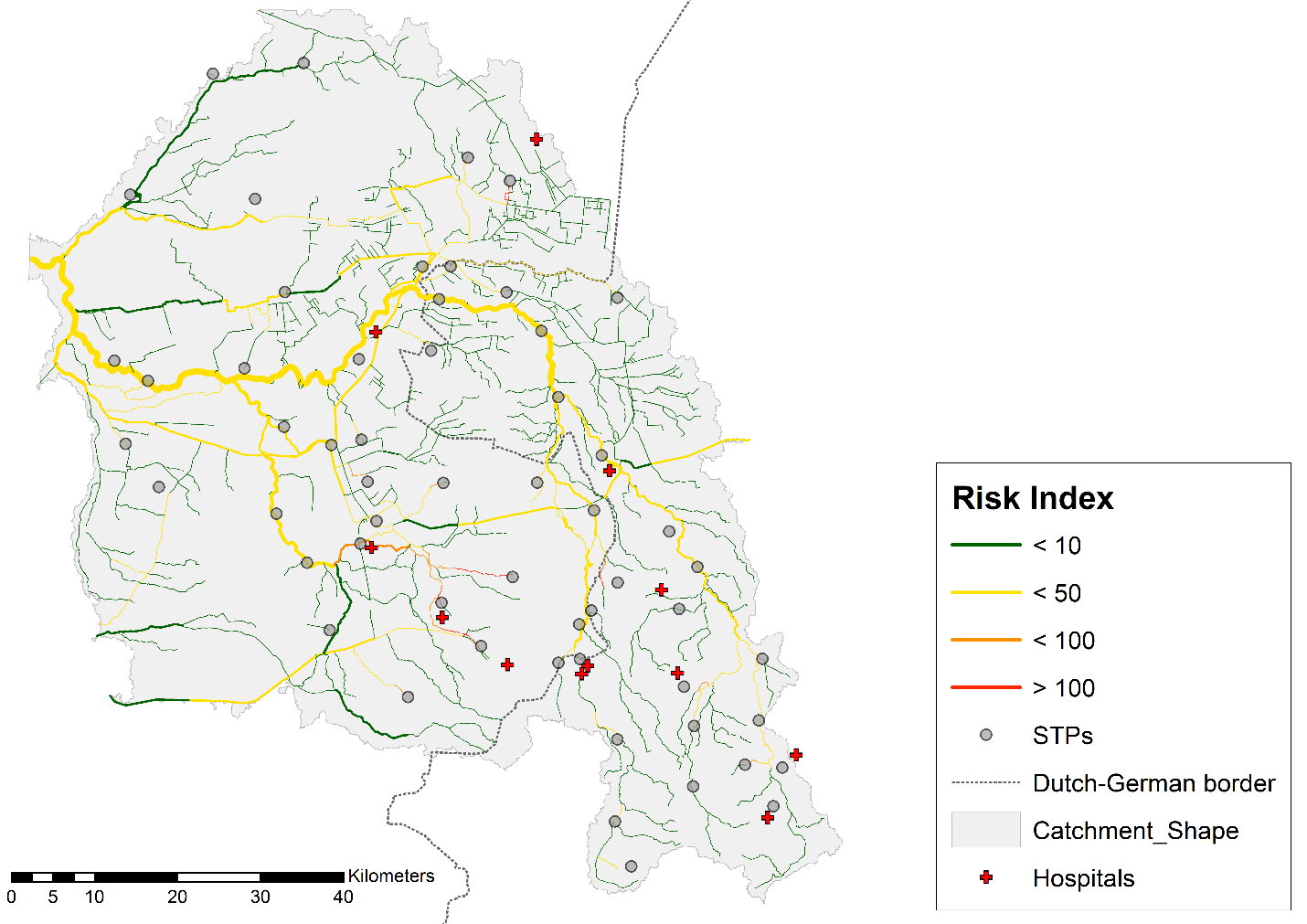


**FIGURE S6:** Risk index map of the Vecht River catchment during a typical average-condition-scenario. Dashed line demarks the German-Dutch border.

**REFERENCES**

Aderemi AO, Novais SC, Lemos MFL, Alves LM, Hunter C, Pahl O. 2018. Oxidative stress responses and cellular energy allocation changes in microalgae following exposure to widely used human antibiotics. Aquatic toxicology (Amsterdam, Netherlands). 203:130–139.

Agerstrand M, Rudén C. 2010. Evaluation of the accuracy and consistency of the Swedish environmental classification and information system for pharmaceuticals. The Science of the total environment. 408(11):2327–2339.

Alder AC, Schaffner C, Majewsky M, Klasmeier J, Fenner K. 2010. Fate of beta-blocker human pharmaceuticals in surface water: comparison of measured and simulated concentrations in the Glatt Valley Watershed, Switzerland. Water research. 44(3):936–948.

Alexy R, Kümpel T, Kümmerer K. 2004. Assessment of degradation of 18 antibiotics in the Closed Bottle Test. Chemosphere. 57(6):505–512.

AMR Industry Alliance. 2018. AMR Industry Alliance Antibiotic Discharge Targets - List of Predicted No-Effect Concentrations (PNECs).

Ando T, Nagase H, Eguchi K, Hirooka T, Nakamura T, Miyamoto K, Hirata K. 2007. A novel method using cyanobacteria for ecotoxicity test of veterinary antimicrobial agents. Environmental toxicology and chemistry. 26(4):601–606.

Andreozzi R, Raffaele M, Nicklas P. 2003. Pharmaceuticals in STP effluents and their solar photodegradation in aquatic environment. Chemosphere. 50(10):1319–1330.

Apell Jennifer N, McNeill K. 2019. Updated and validated solar irradiance reference spectra for estimating environmental photodegradation rates. Environmental Science: Processes & Impacts. 21(3):427-437.

AstraZeneca. 2017a. Environmental Risk Assessment Data – Metformin.

AstraZeneca. 2017b. Environmental Risk Assessment Data – Metoprolol.

Azuma T, Arima N, Tsukada A, Hirami S, Matsuoka R, Moriwake R, Ishiuchi H, Inoyama T, Teranishi Y, Yamaoka M et al. 2017. Distribution of six anticancer drugs and a variety of other pharmaceuticals, and their sorption onto sediments, in an urban Japanese river. Environmental science and pollution research international. 24(23):19021–19030.

Baena-Nogueras RM, González-Mazo E, Lara-Martín PA. 2017. Degradation kinetics of pharmaceuticals and personal care products in surface waters: photolysis vs biodegradation. Sci Total Environ. 590-591:643-654.

Bahlmann A, Brack W, Schneider RJ, Krauss M. 2014. Carbamazepine and its metabolites in wastewater: Analytical pitfalls and occurrence in Germany and Portugal. Water research. 57:104–114.

Batchu SR, Panditi VR, O'Shea KE, Gardinali PR. 2014. Photodegradation of antibiotics under simulated solar radiation: Implications for their environmental fate. Sci Total Environ. 470-471:299-310.

Bayer A, Asner R, Schüssler W, Kopf W, Weiß K, Sengl M, Letzel M. 2014. Behavior of sartans (antihypertensive drugs) in wastewater treatment plants, their occurrence and risk for the aquatic environment. Environmental science and pollution research international. 21(18):10830–10839.

Björlenius B, Ripszám M, Haglund P, Lindberg RH, Tysklind M, Fick J. 2018. Pharmaceutical residues are widespread in Baltic Sea coastal and offshore waters - Screening for pharmaceuticals and modelling of environmental concentrations of carbamazepine. The Science of the total environment. 633:1496–1509.

Boxall ABA, Keller VDJ, Straub JO, Monteiro SC, Fussell R, Williams RJ. 2014. Exploiting monitoring data in environmental exposure modelling and risk assessment of pharmaceuticals. Environ Int. 73:176–185.

Bristol-Myers Squibb Products & Medicines. 2018.

Buerge IJ, Buser H-R, Poiger T, Müller MD. 2006. Occurrence and fate of the cytostatic drugs cyclophosphamide and ifosfamide in wastewater and surface waters. Environmental science & technology. 40(23):7242–7250.

Caldwell DJ, D'Aco V, Davidson T, Kappler K, Murray-Smith RJ, Owen SF, Robinson PF, Simon-Hettich B, Straub JO, Tell J. 2019. Environmental risk assessment of metformin and its transformation product guanylurea: II. Occurrence in surface waters of Europe and the United States and derivation of predicted no-effect concentrations. Chemosphere. 216:855–865.

Calisto V, Domingues MRM, Erny GL, Esteves VI. 2011. Direct photodegradation of carbamazepine followed by micellar electrokinetic chromatography and mass spectrometry. Water research. 45(3):1095–1104.

Carls A, Jedamzik J, Witt L, Hohmann N, Burhenne J, Mikus G. 2014. Systemic exposure of topical erythromycin in comparison to oral administration and the effect on cytochrome P450 3A4 activity. Br J Clin Pharmacol. 78(6):1433-1440.

Castiglioni S, Bagnati R, Fanelli R, Pomati F, Calamari D, Zuccato E. 2006. Removal of pharmaceuticals in sewage treatment plants in Italy. Environmental science & technology. 40(1):357–363.

Česen M, Kosjek T, Laimou-Geraniou M, Kompare B, Širok B, Lambropolou D, Heath E. 2015. Occurrence of cyclophosphamide and ifosfamide in aqueous environment and their removal by biological and abiotic wastewater treatment processes. The Science of the total environment. 527-528:465–473.

Chen H, Gu X, Zeng Q, Mao Z. 2019. Acute and Chronic Toxicity of Carbamazepine on the Release of Chitobiase, Molting, and Reproduction in Daphnia similis. International journal of environmental research and public health. 16(2).

Clara M, Strenn B, Gans O, Martinez E, Kreuzinger N, Kroiss H. 2005. Removal of selected pharmaceuticals, fragrances and endocrine disrupting compounds in a membrane bioreactor and conventional wastewater treatment plants. Water research. 39(19):4797–4807.

Comber S, Gardner M, Sörme P, Leverett D, Ellor B. 2018. Active pharmaceutical ingredients entering the aquatic environment from wastewater treatment works: A cause for concern? The Science of the total environment. 613-614:538–547.

De Liguoro M, Fioretto B, Poltronieri C, Gallina G. 2009. The toxicity of sulfamethazine to Daphnia magna and its additivity to other veterinary sulfonamides and trimethoprim. Chemosphere. 75(11):1519-1524.

Di Poi C, Costil K, Bouchart V, Halm-Lemeille M-P. 2018. Toxicity assessment of five emerging pollutants, alone and in binary or ternary mixtures, towards three aquatic organisms. Environmental science and pollution research international. 25(7):6122–6134.

Dordio AV, Belo M, Martins Teixeira D, Palace Carvalho AJ, Dias CMB, Picó Y, Pinto AP. 2011. Evaluation of carbamazepine uptake and metabolization by Typha spp., a plant with potential use in phytotreatment. Bioresource technology. 102(17):7827–7834.

Durán-Álvarez JC, Prado B, González D, Sánchez Y, Jiménez-Cisneros B. 2015. Environmental fate of naproxen, carbamazepine and triclosan in wastewater, surface water and wastewater irrigated soil - Results of laboratory scale experiments. The Science of the total environment. 538:350–362.

Dutch Foundation for Pharmaceutical Statistics. 2018.

Eguchi K, Nagase H, Ozawa M, Endoh YS, Goto K, Hirata K, Miyamoto K, Yoshimura H. 2004. Evaluation of antimicrobial agents for veterinary use in the ecotoxicity test using microalgae. Chemosphere. 57(11):1733–1738.

European Union. 2011a. Diclofenac EQS dossier.

European Union. 2011b. Ethinylestradiol EQS dossier.

Fabbri R, Montagna M, Balbi T, Raffo E, Palumbo F, Canesi L. 2014. Adaptation of the bivalve embryotoxicity assay for the high throughput screening of emerging contaminants in Mytilus galloprovincialis. Marine Environmental Research. 99:1-8.

Ferrari B, Mons R, Vollat B, Fraysse B, Paxéus N, Lo Giudice R, Pollio A, Garric J. 2004. Environmental risk assessment of six human pharmaceuticals: are the current environmental risk assessment procedures sufficient for the protection of the aquatic environment? Environmental toxicology and chemistry. 23(5):1344–1354.

Frédéric O, Yves P. 2014. Pharmaceuticals in hospital wastewater: their ecotoxicity and contribution to the environmental hazard of the effluent. Chemosphere. 115:31–39.

Gao J, Banks A, Li J, Jiang G, Lai FY, Mueller JF, Thai PK. 2017. Evaluation of in-sewer transformation of selected illicit drugs and pharmaceutical biomarkers. Sci Total Environ. 609:1172-1181.

Gheorghe S, Petre J, Lucaciu I, Stoica C, Nita-Lazar M. 2016. Risk screening of pharmaceutical compounds in Romanian aquatic environment. Environmental monitoring and assessment. 188(6):379.

Girardi C, Greve J, Lamshöft M, Fetzer I, Miltner A, Schäffer A, Kästner M. 2011. Biodegradation of ciprofloxacin in water and soil and its effects on the microbial communities. Journal of hazardous materials. 198:22–30.

Göbel A, McArdell CS, Joss A, Siegrist H, Giger W. 2007. Fate of sulfonamides, macrolides, and trimethoprim in different wastewater treatment technologies. The Science of the total environment. 372(2-3):361–371.

Göbel A, Thomsen A, McArdell CS, Joss A, Giger W. 2005. Occurrence and sorption behavior of sulfonamides, macrolides, and trimethoprim in activated sludge treatment. Environmental science & technology. 39(11):3981–3989.

Godoy AA, Domingues I, Arsénia Nogueira AJ, Kummrow F. 2018. Ecotoxicological effects, water quality standards and risk assessment for the anti-diabetic metformin. Environmental pollution (Barking, Essex : 1987). 243(Pt A):534–542.

González-Pleiter M, Gonzalo S, Rodea-Palomares I, Leganés F, Rosal R, Boltes K, Marco E, Fernández-Piñas F. 2013. Toxicity of five antibiotics and their mixtures towards photosynthetic aquatic organisms: implications for environmental risk assessment. Water research. 47(6):2050–2064.

Grung M, Källqvist T, Sakshaug S, Skurtveit S, Thomas KV. 2008. Environmental assessment of Norwegian priority pharmaceuticals based on the EMEA guideline. Ecotoxicology and environmental safety. 71(2):328–340.

Guerra P, Kim M, Shah A, Alaee M, Smyth SA. 2014. Occurrence and fate of antibiotic, analgesic/anti-inflammatory, and antifungal compounds in five wastewater treatment processes. The Science of the total environment. 473-474:235–243.

Gurke R, Rößler M, Marx C, Diamond S, Schubert S, Oertel R, Fauler J. 2015. Occurrence and removal of frequently prescribed pharmaceuticals and corresponding metabolites in wastewater of a sewage treatment plant. The Science of the total environment. 532:762–770.

Han GH, Hur HG, Kim SD. 2006. Ecotoxicological risk of pharmaceuticals from wastewater treatment plants in Korea: occurrence and toxicity to Daphnia magna. Environmental toxicology and chemistry. 25(1):265–271.

He J-H, Guo S-Y, Zhu F, Zhu J-J, Chen Y-X, Huang C-J, Gao J-M, Dong Q-X, Xuan Y-X, Li C-Q. 2013. A zebrafish phenotypic assay for assessing drug-induced hepatotoxicity. Journal of pharmacological and toxicological methods. 67(1):25–32.

Heberer T, Feldmann D. 2005. Contribution of effluents from hospitals and private households to the total loads of diclofenac and carbamazepine in municipal sewage effluents--modeling versus measurements. Journal of hazardous materials. 122(3):211–218.

Heye K, Wiebusch J, Becker J, Rongstock L, Bröder K, Wick A, Schulte-Oehlmann U, Oehlmann J. 2019. Ecotoxicological characterization of the antiepileptic drug carbamazepine using eight aquatic species: baseline study for future higher tier tests. Journal of environmental science and health Part A, Toxic/hazardous substances & environmental engineering. 54(5):441–451.

Hoeger B, Köllner B, Dietrich DR, Hitzfeld B. 2005. Water-borne diclofenac affects kidney and gill integrity and selected immune parameters in brown trout (Salmo trutta f. fario). Aquatic toxicology (Amsterdam, Netherlands). 75(1):53–64.

Hui X, Hewitt PG, Poblete N, Maibach HI, Shainhouse JZ, Wester RC. 1998. In vivo bioavailability and metabolism of topical diclofenac lotion in human volunteers. Pharmaceutical research. 15(10):1589–1595.

Jarvis AL, Bernot MJ, Bernot RJ. 2014. The effects of the pharmaceutical carbamazepine on life history characteristics of flat-headed mayflies (Heptageniidae) and aquatic resource interactions. Ecotoxicology (London, England). 23(9):1701–1712.

Jesus Gaffney Vd, Cardoso VV, Cardoso E, Teixeira AP, Martins J, Benoliel MJ, Almeida CMM. 2017. Occurrence and behaviour of pharmaceutical compounds in a Portuguese wastewater treatment plant: Removal efficiency through conventional treatment processes. Environmental science and pollution research international. 24(17):14717–14734.

Ji K, Kim S, Han S, Seo J, Lee S, Park Y, Choi K, Kho Y-L, Kim P-G, Park J et al. 2012. Risk assessment of chlortetracycline, oxytetracycline, sulfamethazine, sulfathiazole, and erythromycin in aquatic environment: are the current environmental concentrations safe? Ecotoxicology (London, England). 21(7):2031–2050.

Johnson AC, Keller V, Williams RJ, Young A. 2007. A practical demonstration in modelling diclofenac and propranolol river water concentrations using a GIS hydrology model in a rural UK catchment. Environmental pollution (Barking, Essex : 1987). 146(1):155–165.

Johnson AC, Williams RJ. 2004. A model to estimate influent and effluent concentrations of estradiol, estrone, and ethinylestradiol at sewage treatment works. Environmental science & technology. 38(13):3649–3658.

Jungmann D, Berg K, Dieterich A, Frank M, Gräf T, Scheurer M, Schwarz S, Siewert C, Oetken M. 2017. Health effects of metoprolol in epibenthic and endobenthic invertebrates-A basis to validate future in vitro biotests for effect-based biomonitoring. Journal of environmental science and health Part A, Toxic/hazardous substances & environmental engineering. 52(3):189–200.

Jürgens MD, Holthaus KIE, Johnson AC, Smith JJL, Hetheridge M, Williams RJ. 2002. The potential for estradiol and ethinylestradiol degradation in english rivers. Environmental toxicology and chemistry. 21(3):480–488.

Kasprzyk-Hordern B, Dinsdale RM, Guwy AJ. 2009. The removal of pharmaceuticals, personal care products, endocrine disruptors and illicit drugs during wastewater treatment and its impact on the quality of receiving waters. Water research. 43(2):363–380.

Khan SJ, Ongerth JE. 2004. Modelling of pharmaceutical residues in Australian sewage by quantities of use and fugacity calculations. Chemosphere. 54(3):355–367.

Kumar V, Johnson AC, Nakada N, Yamashita N, Tanaka H. 2012. De-conjugation behavior of conjugated estrogens in the raw sewage, activated sludge and river water. Journal of Hazardous Materials. 227-228:49-54.

Kummerer K, Menz J, Schubert T, Thielemans W. 2011. Biodegradability of organic nanoparticles in the aqueous environment. Chemosphere. 82(10):1387-1392.

Lahti M, Oikari A. 2011. Microbial transformation of pharmaceuticals naproxen, bisoprolol, and diclofenac in aerobic and anaerobic environments. Archives of environmental contamination and toxicology. 61(2):202–210.

Li B, Zhang T. 2011. Mass flows and removal of antibiotics in two municipal wastewater treatment plants. Chemosphere. 83(9):1284–1289.

Li Z-H, Zlabek V, Velisek J, Grabic R, Machova J, Randak T. 2010. Physiological condition status and muscle-based biomarkers in rainbow trout (Oncorhynchus mykiss), after long-term exposure to carbamazepine. Journal of applied toxicology : JAT. 30(3):197–203.

FASS database. 2019. The Trade Association for the Research-Based Pharmaceutical Industry in Sweden; [accessed March 2019]. <https://www.fass.se/>.

Loos R, Marinov D, SANSEVERINO I, Napierska D, Lettieri T. 2018. Review of the 1st Watch List under the Water Framework Directive and recommendations for the 2nd Watch List. Luxembourg: EU-Joint Research Centre.

Lutterbeck CA, Wilde ML, Baginska E, Leder C, Machado ÊL, Kümmerer K. 2016. Degradation of cyclophosphamide and 5-fluorouracil by UV and simulated sunlight treatments: Assessment of the enhancement of the biodegradability and toxicity. Environmental pollution (Barking, Essex : 1987). 208(Pt B):467–476.

Majewska M, Harshkova D, Guściora M, Aksmann A. 2018. Phytotoxic activity of diclofenac: Evaluation using a model green alga Chlamydomonas reinhardtii with atrazine as a reference substance. Chemosphere. 209:989–997.

Martins N, Pereira R, Abrantes N, Pereira J, Gonçalves F, Marques CR. 2012. Ecotoxicological effects of ciprofloxacin on freshwater species: data integration and derivation of toxicity thresholds for risk assessment. Ecotoxicology (London, England). 21(4):1167–1176.

Moermond CTA. 2014. Environmental Risk Limits for Pharmaceuticals: Derivation of WFD Water Quality Standards for Carbamazepine, metoprolol, metformin and Amidotrizoic Acid. Bilthoven, The Netherlands: National Institute for Public Health and the Environment (RIVM). No. 270006002/2014.

Moermond CTA, Smit CE. 2016. Derivation of water quality standards for carbamazepine, metoprolol, and metformin and comparison with monitoring data. Environmental toxicology and chemistry. 35(4):882–888.

Moffat AC, Osselton MD, Widdop B, Watts J. 2011. Clarke's Analysis of Drugs and Poisons. London and Chicago: Pharmaceutical Press.

Monika Z-R, Maria Ł, Affek K, Zarzeczna A. 2011. Environmental risk assessment of selected pharmaceuticals present in surface waters in relation to animals. Archives of Environmental Protection. 37:31-42.

Murray-Smith RJ, Coombe VT, Grönlund MH, Waern F, Baird JA. 2012. Managing emissions of active pharmaceutical ingredients from manufacturing facilities: an environmental quality standard approach. Integrated environmental assessment and management. 8(2):320–330.

Nakada N, Shinohara H, Murata A, Kiri K, Managaki S, Sato N, Takada H. 2007. Removal of selected pharmaceuticals and personal care products (PPCPs) and endocrine-disrupting chemicals (EDCs) during sand filtration and ozonation at a municipal sewage treatment plant. Water research. 41(19):4373–4382.

Neamţu M, Grandjean D, Sienkiewicz A, Le Faucheur S, Slaveykova V, Colmenares JJV, Pulgarín C, Alencastro LFd. 2014. Degradation of eight relevant micropollutants in different water matrices by neutral photo-Fenton process under UV254 and simulated solar light irradiation – A comparative study. Applied Catalysis B: Environmental. 158-159:30–37.

NORMAN Substance Database. 2019. [accessed]. <https://www.norman-network.com>.

Oekotoxzentrum. 2016a. EQS Proposal for Carbamazepine and Main Transformation Products. Dübendorf, Switzerland: Swiss Federal Institute of Aquatic Science and Technology (Eawag).

Oekotoxzentrum. 2016b. EQS Proposal for Metformin and Main Transformation Products. Dübendorf, Switzerland: Swiss Federal Institute of Aquatic Science and Technology (Eawag).

Oekotoxzentrum. 2016c. EQS Proposal for Metoprolol. Dübendorf, Switzerland: Swiss Federal Institute of Aquatic Science and Technology (Eawag).

Ofoegbu PU, Lourenço J, Mendo S, Soares AMVM, Pestana JLT. 2019. Effects of low concentrations of psychiatric drugs (carbamazepine and fluoxetine) on the freshwater planarian, Schmidtea mediterranea. Chemosphere. 217:542–549.

omitted author ea. unpublished manuscript.

Oosterhuis M, Sacher F, ter Laak TL. 2013. Prediction of concentration levels of metformin and other high consumption pharmaceuticals in wastewater and regional surface water based on sales data. The Science of the total environment. 442:380–388.

Perazzolo C, Morasch B, Kohn T, Magnet A, Thonney D, Chèvre N. 2010. Occurrence and fate of micropollutants in the Vidy Bay of Lake Geneva, Switzerland. Part I: priority list for environmental risk assessment of pharmaceuticals. Environmental toxicology and chemistry. 29(8):1649–1657.

Radjenovic J, Petrovic M, Barceló D. 2007. Analysis of pharmaceuticals in wastewater and removal using a membrane bioreactor. Analytical and bioanalytical chemistry. 387(4):1365–1377.

Radjenović J, Petrović M, Barceló D. 2009. Fate and distribution of pharmaceuticals in wastewater and sewage sludge of the conventional activated sludge (CAS) and advanced membrane bioreactor (MBR) treatment. Water research. 43(3):831–841.

Radović TT, Grujić SD, Kovačević SR, Laušević MD, Dimkić MA. 2016. Sorption of selected pharmaceuticals and pesticides on different river sediments. Environmental science and pollution research international. 23(24):25232–25244.

Regårdh CG, Borg KO, Johansson R, Johnsson G, Palmer L. 1974. Pharmacokinetic studies on the selective beta1-receptor antagonist metoprolol in man. Journal of pharmacokinetics and biopharmaceutics. 2(4):347–364.

Robert F, Fendri S, Hary L, Lacroix C, Andréjak M, Lalau JD. 2003. Kinetics of plasma and erythrocyte metformin after acute administration in healthy subjects. Diabetes & Metabolism. 29(3):279–283.

Roberts PH, Thomas KV. 2006. The occurrence of selected pharmaceuticals in wastewater effluent and surface waters of the lower Tyne catchment. The Science of the total environment. 356(1-3):143–153.

Russo C, Lavorgna M, Česen M, Kosjek T, Heath E, Isidori M. 2018. Evaluation of acute and chronic ecotoxicity of cyclophosphamide, ifosfamide, their metabolites/transformation products and UV treated samples. Environmental pollution (Barking, Essex : 1987). 233:356–363.

Sacher F. 2014. Spurenstoffinventar der Fließgewässer in Baden-Württemberg: Ergebnisse der Beprobung von Fließgewässern und Kläranlagen 2012/2013. Karlsruhe: LUBW.

Scheurer M, Michel A, Brauch H-J, Ruck W, Sacher F. 2012. Occurrence and fate of the antidiabetic drug metformin and its metabolite guanylurea in the environment and during drinking water treatment. Water research. 46(15):4790–4802.

Senta I, Kostanjevecki P, Krizman-Matasic I, Terzic S, Ahel M. 2019. Occurrence and Behavior of Macrolide Antibiotics in Municipal Wastewater Treatment: Possible Importance of Metabolites, Synthesis Byproducts, and Transformation Products. Environmental science & technology. 53(13):7463–7472.

Sioufi A, Pommier F, Boschet F, Godbillon J, Lavoignat D, Salliere D. 1994. Percutaneous absorption of diclofenac in healthy volunteers after single and repeated topical application of diclofenac Emulgel. Biopharmaceutics & drug disposition. 15(6):441–449.

Sui Q, Huang J, Deng S, Chen W, Yu G. 2011. Seasonal variation in the occurrence and removal of pharmaceuticals and personal care products in different biological wastewater treatment processes. Environmental science & technology. 45(8):3341–3348.

Suter II GW. 2007. Ecological Risk Assessment. 2nd Edition ed. Boca Raton: CRC Press. p. 674.

Swiss Agency for Therapeutic Products. 2020. [accessed 2020]. <https://www.swissmedicinfo.ch>.

Ternes T, Joss A. 2008. Human pharmaceuticals, hormones and fragrances: The challenge of micropollutants in urban water management. Reprinted. ed. London: IWA Publ. p. 453.

Ternes TA, Bonerz M, Herrmann N, Teiser B, Andersen HR. 2007. Irrigation of treated wastewater in Braunschweig, Germany: an option to remove pharmaceuticals and musk fragrances. Chemosphere. 66(5):894–904.

Ternes TA, Herrmann N, Bonerz M, Knacker T, Siegrist H, Joss A. 2004. A rapid method to measure the solid-water distribution coefficient (Kd) for pharmaceuticals and musk fragrances in sewage sludge. Water research. 38(19):4075–4084.

Thomas KV, Dye C, Schlabach M, Langford KH. 2007. Source to sink tracking of selected human pharmaceuticals from two Oslo city hospitals and a wastewater treatment works. Journal of environmental monitoring : JEM. 9(12):1410–1418.

Tolls J. 2001. Sorption of veterinary pharmaceuticals in soils: a review. Environmental science & technology. 35(17):3397–3406.

Trautwein C, Kümmerer K. 2011. Incomplete aerobic degradation of the antidiabetic drug Metformin and identification of the bacterial dead-end transformation product Guanylurea. Chemosphere. 85(5):765–773.

Triebskorn R, Casper H, Scheil V, Schwaiger J. 2007. Ultrastructural effects of pharmaceuticals (carbamazepine, clofibric acid, metoprolol, diclofenac) in rainbow trout (Oncorhynchus mykiss) and common carp (Cyprinus carpio). Analytical and bioanalytical chemistry. 387(4):1405–1416.

Tucker GT, Casey C, Phillips PJ, Connor H, Ward JD, Woods HF. 1981. Metformin kinetics in healthy subjects and in patients with diabetes mellitus. British journal of clinical pharmacology. 12(2):235–246.

van der Aa NGFM, van Vlaardingen PLA, van Leeuwen LC, Post M. 2011. Assessment of potential risks of11 pharmaceuticals for the environment - Using environmental information from public databases. Bilthoven, The Netherlands: National Institute for Public Health and the Environment (RIVM).

van Vlaardingen PLA, de Poorter LRM, Fleuren RHLJ, Janssen PJCM, Posthuma-Doodeman CJAM, Verbruggen EMJ, Vos JH. 2007. Environmental risk limits for twelve substances, prioritised on the basis of indicative risk limits. Bilthoven, The Netherlands: National Institute for Public Health and the Environment (RIVM).

Vergeynst L, Haeck A, Wispelaere Pd, van Langenhove H, Demeestere K. 2015. Multi-residue analysis of pharmaceuticals in wastewater by liquid chromatography-magnetic sector mass spectrometry: method quality assessment and application in a Belgian case study. Chemosphere. 119 Suppl:S2-8.

Vestel J, Caldwell DJ, Constantine L, D'Aco VJ, Davidson T, Dolan DG, Millard SP, Murray-Smith R, Parke NJ, Ryan JJ et al. 2016. Use of acute and chronic ecotoxicity data in environmental risk assessment of pharmaceuticals. Environmental toxicology and chemistry. 35(5):1201–1212.

Vieno NM, Tuhkanen T, Kronberg L. 2006. Analysis of neutral and basic pharmaceuticals in sewage treatment plants and in recipient rivers using solid phase extraction and liquid chromatography-tandem mass spectrometry detection. Journal of chromatography A. 1134(1-2):101–111.

Wenzel A, Shemotyuk L. 2014. EQS Datasheet: Environmental Quality Standard Carbamazepine. Dessau-Roßlau, Germany: Federal Environmental Agency (UBA).

Wick A, Fink G, Joss A, Siegrist H, Ternes TA. 2009. Fate of beta blockers and psycho-active drugs in conventional wastewater treatment. Water research. 43(4):1060–1074.

Yang L-H, Ying G-G, Su H-C, Stauber JL, Adams MS, Binet MT. 2008. Growth-inhibiting effects of 12 antibacterial agents and their mixtures on the freshwater microalga Pseudokirchneriella subcapitata. Environmental toxicology and chemistry. 27(5):1201–1208.

Yokota H, Taguchi Y, Tanaka Y, Uchiyama M, Kondo M, Tsuruda Y, Suzuki T, Eguchi S. 2018. Chronic exposure to diclofenac induces delayed mandibular defects in medaka (Oryzias latipes) in a sex-dependent manner. Chemosphere. 210:139–146.

Zhang Y, Geissen S-U, Gal C. 2008. Carbamazepine and diclofenac: removal in wastewater treatment plants and occurrence in water bodies. Chemosphere. 73(8):1151–1161.

Zhu J-J, Xu Y-Q, He J-H, Yu H-P, Huang C-J, Gao J-M, Dong Q-X, Xuan Y-X, Li C-Q. 2014. Human cardiotoxic drugs delivered by soaking and microinjection induce cardiovascular toxicity in zebrafish. Journal of applied toxicology : JAT. 34(2):139–148.

Zounková R, Odráska P, Dolezalová L, Hilscherová K, Marsálek B, Bláha L. 2007. Ecotoxicity and genotoxicity assessment of cytostatic pharmaceuticals. Environmental toxicology and chemistry. 26(10):2208–2214.

Zuo Y, Zhang K, Zhou S. 2013. Determination of estrogenic steroids and microbial and photochemical degradation of 17α-ethinylestradiol (EE2) in lake surface water, a case study. Environmental science Processes & impacts. 15(8):1529–1535.
